# The Genome of the “Sea Vomit” *Didemnum vexillum*

**DOI:** 10.3390/life11121377

**Published:** 2021-12-10

**Authors:** Ernesto Parra-Rincón, Cristian A. Velandia-Huerto, Adriaan Gittenberger, Jörg Fallmann, Thomas Gatter, Federico D. Brown, Peter F. Stadler, Clara I. Bermúdez-Santana

**Affiliations:** 1 Biology Department, Universidad Nacional de Colombia, Carrera 45 # 26-85, Edif. Uriel Gutiérrez, Bogotá D.C 111321, Colombia; eparrar@unal.edu.co (E.P.-R.); studla@bioinf.uni-leipzig.de (P.F.S.); 2 Bioinformatics Group, Department of Computer Science, and Interdisciplinary Center for Bioinformatics, Leipzig University, 04107 Leipzig, Germany; fall@bioinf.uni-leipzig.de (J.F.); thomas@bioinf.uni-leipzig.de (T.G.); 3 GiMaRIS, Rijksstraatweg 75, 2171 AK Sassenheim, The Netherlands; Gittenberger@GiMaRIS.com; 4 Institute of Biology, Leiden University, P.O. Box 9505, 2300 RA Leiden, The Netherlands; 5 Naturalis Biodiversity Center, Darwinweg 2, 2333 CR Leiden, The Netherlands; 6 Departamento de Zoologia, Instituto Biociências, Universidade de São Paulo, Rua do Matão, Tr. 14 no. 101, São Paulo 05508-090, Brazil; fdbrown@usp.br; 7 Centro de Biologia Marinha, Universidade de São Paulo, Rod. Manuel Hypólito do Rego km. 131.5, São Sebastião 11612-109, Brazil; 8 Max Planck Institute for Mathematics in the Sciences, 04103 Leipzig, Germany; 9 Institute for Theoretical Chemistry, University of Vienna, 1090 Vienna, Austria; 10 Santa Fe Institute, Santa Fe, NM 87506, USA

**Keywords:** Tunicata, *Didemnum vexillum*, microRNAs, genome annotation

## Abstract

Tunicates are the sister group of vertebrates and thus occupy a key position for investigations into vertebrate innovations as well as into the consequences of the vertebrate-specific genome duplications. Nevertheless, tunicate genomes have not been studied extensively in the past, and comparative studies of tunicate genomes have remained scarce. The carpet sea squirt *Didemnum vexillum*, commonly known as “sea vomit”, is a colonial tunicate considered an invasive species with substantial ecological and economical risk. We report the assembly of the *D. vexillum* genome using a hybrid approach that combines 28.5 Gb Illumina and 12.35 Gb of PacBio data. The new hybrid scaffolded assembly has a total size of 517.55 Mb that increases contig length about eightfold compared to previous, Illumina-only assembly. As a consequence of an unusually high genetic diversity of the colonies and the moderate length of the PacBio reads, presumably caused by the unusually acidic milieu of the tunic, the assembly is highly fragmented (L50 = 25,284, N50 = 6539). It is sufficient, however, for comprehensive annotations of both protein-coding genes and non-coding RNAs. Despite its shortcomings, the draft assembly of the “sea vomit” genome provides a valuable resource for comparative tunicate genomics and for the study of the specific properties of colonial ascidians.

## 1. Introduction

The carpet sea squirt *Didemnum vexillum* [1], commonly called “sea vomit”, “marine vomit”, “pancake batter tunicate”, or “carpet sea squirt”, is a colonial tunicate presumably native to Japan that has appeared as an invasive species in Europe, the Americas, and New Zealand [2]. It negatively affects established benthic species and damages ship hulls as well as the infrastructure in marinas, ports, and shellfish farms.

Rapid colony growth or regression in response to the dynamics of the habitat [3], water temperature [4], colony fragmentation as a reproductive and dispersal strategy [5], fast asexual budding that allows attachment to a variety of living and/or non-living substrata, and relatively few predators [3] have facilitated *D. vexillum* to become a well-recognized worldwide invader. The invasion potential of *D. vexillum* has an important economic impact on the aquaculture industry as it affects the conditions of bivalve and shellfish cultures (see, e.g., in [6] and the references therein), and increases the cost of maintenance to avoid the fouling process on mussel cages and facilities [7].

Despite the economic impact of tunicates and their pivotal phylogenetic position as sister group of the vertebrates, genomic studies and comparative analyses have remained relatively scarce. So far, the genomes of four solitary tunicates have been assembled and annotated in substantial depth. Assemblies of the 14 chromosomes of the closely related sessile ascidians *Ciona savignyi* and *Ciona robusta* (formerly identified as *Ciona intestinalis* type A, refer to the work in  [8] for details) are available  [9,10,11,12]. The genome of *Styela clava* [13] was assembled into 16 chromosomes. For the pelagic larvacean *Oikopleura dioica*, ≤6 chromosomes have been reported [14,15,16] and recently populations from Hyogo [17] and Okinawa [18] in Japan were sequenced. In addition, draft assemblies recently have become available for the pelagic colonial thaliacian *Salpa thompsoni*, which was used to analyze the high mutation rates in the genomes of tunicates [19]. Other genomic studies in tunicates include four solitary ascidians, the genome of *Halocynthia roretzi* was used to predict microRNAs [20], and the genomes of three species of *Molgula* (*Molgula occidentalis*, *Molgula oculata* and *Molgula occulta*) that lead to the study of drift in the developmental system responsible for cardiopharyngeal development [21]. In addition, the genome of the *Corella inflata* [22] provided a significant update of tunicate phylogeny, supporting the paraphyly of Phlebobranchia, and contributed a description of the Hox cluster evolution together with analysis of cardiovascular-associated genes. At the same time, the ANISEED database [23] served as a hub for ongoing sequencing projects for other ascidian species, including *Phallusia mammillata*, *Phallusia fumigata*, and *Halocynthia aurantium*. For colonial ascidians, the genome of *Botryllus schlosseri* has been assembled to 13 incomplete chromosomes (of the 16 chromosomes in total) [24], and a draft assembly comprising 1778 scaffolds has been reported for the related species *Botrylloides leachii* [25]. A very fragmented assembly of the “sea vomit” *D. vexillum* was also recently sequenced by our group to analyze non-coding RNAs (ncRNAs) [26]. Here, we report on a substantial improvement of this assembly after overcoming experimental and computational difficulties.

Comparisons between tunicate and other chordate genomes have identified both expansions of gene families and innovations such as the horizontally transferred genes of cellulose synthase from Actinobacteria [27], but also substantial losses, e.g., of parts of the *homeobox* (HOX) gene cluster [25]. The genomic organization of tunicates, as exemplified by *Ciona* and *Oikopleura*, shows substantial differences compared to both vertebrates and amphioxus, the common outgroup to the Olfactores [28], and has led to the hypothesis that tunicates have undergone major genomic restructuring because of an accelerated rate of evolution that was linked to changes in the organization of the entire gene complements [19,29,30]. In contrast, other chordate lineages have maintained a fairly constant rate of evolution [19,29,30].

In this study we expand the assembly and annotation of tunicate genomic resources, and improve the current genome assembly of the colonial tunicate *D. vexillum* producing a resource to contribute to unravel the origins of chordates, as well as to improve our comprehension of the genomic changes involved in the novel mechanisms of asexual reproduction of colonial animals.

## 2. Materials and Methods

### 2.1. DNA and RNA Sequencing

#### 2.1.1. DNA Extraction

From June 2015 to February 2016, 29 DNA extractions from colonies collected in the marine lake Grevelingen, The Netherlands (coordinates: $51^{\circ }$45.073${}^{\prime }$ N, $3^{\circ }$55.664${}^{\prime }$ E), were conducted. DNA from these extractions, however, was too fragmented for further genomic analyses upon arrival in the laboratories. The material analyzed here belongs to a *Didemnum vexillum* colony collected during the third week of November 2015. Directly after collection, it was stored in ethanol in the −20${}^{\circ }$C freezer and used for 8 separate DNA-extractions in the second week of December 2015.

Eight fragments with a diameter of about 4 mm each, were cut out from this colony with a sterile scalpel for DNA-extraction. A Kingfisherflex robot was used to extract the DNA from these pieces with the Nucleomag Tissue kit from Macherey Nagel. To lyse the cells, 200 μL T1 lysis buffer were added to the wells of a 96-well plate. Eight of these wells were used for the DNA extraction of *D. vexillum*. After adding a small piece of tissue, 25 μL of Proteinase K (20 mg/mL) was added and incubated at 56 ${}^{\circ }$C overnight. After the cells were lysed, 225 μL of the sample was added to the MB2 plate containing 360 μL MB2 binding buffer (35-55% ethanol, 20-40% sodium perchlorate), and 25 μL Magnetic beads. The robot then mixed the mixture and transferred the DNA that was attached to the magnetic beads to a series of wash buffers (20–30% ethanol). The MB3 plate was filled with 600 μL MB3 wash buffer, the MB4 plate with 600 μL MB4 wash buffer, and the MB5 plate with 600 μL MB5 wash buffer. To release the DNA from the magnetic beads, the robot proceeded after the wash buffer to the MB6 plate with 150 μL MB6 elution buffer (5 mM Tris/HCl, pH 8.5). The DNA dilution was stored in the fridge at 4 ${}^{\circ }$C. Quality and quantity was tested with the Nanodrop ND1000 for each of the 8 DNA extractions. Based on these analyses two samples of 100 μL each were selected and sent to University of Washington PacBio Sequencing Services for further analysis. Agilent 2100 Bioanalyzer platform was used for the quantitation and sizing of gDNA. PacBio sequencing started from 386 ng/μL and 192 ng/μL quantified by a Qubit assay for each sample respectively.

#### 2.1.2. Partial Degradation of Genomic DNA

As mentioned above, only the two extractions reporting the least severe partial DNA-degradation (∼30–55% of initially sampled DNA) were used for further analysis and final PacBio sequencing. DNA profiling reported a fragmented sample based on quality control analysis (more details in Table S1) for all other extractions (n=27). For selected extractions E6 and E7, fragment size was quantified using Agilent 2100 Bioanalyzer at the University of Washington PacBio Sequencing Service Facility showing that pick size distribution was 2 Kb with a fraction of longer fragments presented.

#### 2.1.3. RNA Extraction

A ∼10 cm${}^{2}$ large piece of a *D. vexillum* colony was collected on 14 December 2009 from the upside of a settlement plate that was deployed about six months earlier on 25 March 2009 at a depth of 1 meter from the south pier of the islet Hompelvoet (Grevelingen, The Netherlands) in an enclosed marine lake with minimal tidal differences. One piece of this colony was used for the first draft in 2016 [26], while another piece of the same colony was used for transcriptome analyses. This piece was preserved in RNAlater (Ambion) at -20 ${}^{\circ }$C prior to RNA extraction and subsequent sequencing in February 2010. Total RNA was extracted using the RNeasy kit according to manufacturer’s instructions (QIAGEN GmbH, Hilden). A transcriptome library was prepared from 10 mg total RNA, using the Illumina mRNA-Seq Sample Preparation Kit according to the manufacturer’s instructions (Illumina Inc., San Diego, CA, USA). The mRNA-Seq library with a read length of 2 × 76 nucleotides was sequenced using the next generation sequencing apparatus Illumina GAIIx according to the manufacturer’s description at ZF-Screens.

#### 2.1.4. Genome Sequencing

The Illumina data used for the genome assembly are described in more detail in [26]. They comprise a mix of paired end reads of 76 and 151 nt, respectively, with a total coverage of about 30× obtained on an Illumina GAIIx instrument.

PacBio sequencing data were obtained using P6/C4 chemistry in an instrument PacBio RSII at University of Washington PacBio Sequencing Services. SMRT libraries of size 20 kb, 10 kb, and 5 kb were run on eleven SMRT cells and prepared without previous DNA shearing or size-selection [31] due to low DIN of the samples (DIN ≤ 3.8). A total of 12.35 Gb sequence data obtained corresponds to 5 millions of subreads with N50 = 2.3 Kbp. Size distribution of sequenced subreads is shown in Figure S1.

### 2.2. Assembly of *D. vexillum* Genome

#### 2.2.1. Data Preprocessing and Pre-Assembling

We opted for a hybrid, non-conservative *de novo* assembly approach. Therefore, PacBio subreads and high-confidence Illumina paired end reads previously used to draft the genome of *D. vexillum* [26] were collectively employed to provide an improved new genome assembly. Before performing the assembly, PacBio sequence data were error corrected and preprocessed in three independent steps. First, PacBio reads of size ≥250 bp and quality ≥0.83 were pre-assembled using the protocols RS_PreAssembler and RS_ReadsOfInsert implemented on SMRT pipe v.2.3. Spurious contigs with chimerics were detected in this step by RS_PreAssembler. A total of 1.4 Gpb comprised of 823,758 pre-assembled reads (error-corrected reads) with N50 = 1.8 Kbp were obtained. Second, a total of 450 Mbp distributed in 220.514 CCSs with quality ≥99% and N50 = 2.1 Kbp were obtained using RS_ReadsOfInsert by processing PacBio reads of complete sequencing cycles ≥2. Third PacBio subreads of size >150 bp and quality >0.87 were retrieved using dextract [32] to be error corrected by the alignments of Illumina PE reads using Proovread-2.13.13 [33]; the PacBio CCS reads was utilized to improve correction performance.

Then, 2.7 Gpb of sequence corrected data comprising 776,295 of untrimmed error-corrected subreads N50 = 3.4 kbp and 391 Mbp of trimmed error-corrected subreads corresponding to 288,198 N50 = 1.7 kbp were obtained. In this step, a second correction of chimeric data presented in the preassembly was computed by Proovread-2.13.13. Finally, a total of (4.94 Gb) of error-corrected data were assembled. The size of the data used for the new hybrid assembly is shown in Table S2.

#### 2.2.2. Contig-Level Assembly

**Figure 1:**
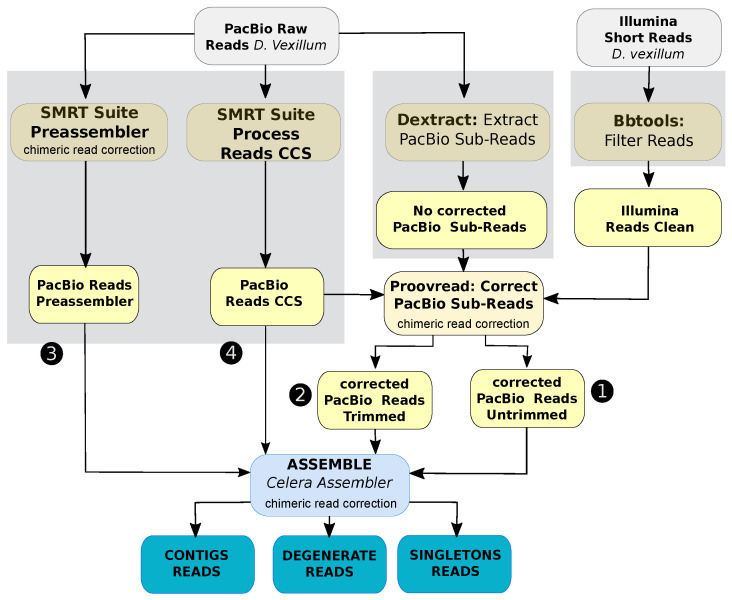
General procedure of the hybrid assembly of *D. vexillum* using error-corrected subreads. Numbers 1–4 correspond to the data size (shown in Table S2). In yellow the steps for data preprocessing and pre-assembling are shown. In blue the contig level assembly of *D. vexillum* genome by Celera Assembler. Three rounds of chimeric corrections are linked to the corresponding step: one run on the PacBio Subreads using of SMRT Suite Preassembler to reduce spurious contigs in the pre-assembled. The second correction was computed under the correction of SMRT reads using the Illumina data as it is implemented in Proovread-2.13.13. Finally, in a third round of chimeric detection was computed using normal doChimeraDetection by Celera Assembler and doOBT=1.

*De novo* hybrid assembly was performed using Celera Assembler Approach [34], Version 8.3rc2, without popping bubbles. Command-line parameters used were utgErrorRate = 0.12, utgErrorLimit = 2.5, ovlErrorRate = 0.15, cgwErrorRate = 0.15, and kmer = 17. Chimeric detection was computed using normal doChimeraDetection by Celera Assembler and doOBT=1. A first version produced an assembly of 566.4 Mpb comprising 130,707 contigs with N50 =5.97 kb and GC=36%. Summary of general steps followed to perform the genome assembly are shown in Figure 1. Redundant contigs were filtered using fasta2homozygous [35]. A total of 16,839 contigs of size ≤500 bp and similarity ≥95% corresponding to 47.4 Mb were removed. Finally, only 519 Mbp were subjected to genome scaffolding.

#### 2.2.3. Genome Scaffolding

LoRDEc [36] was run to correct high-quality CCS by processing together Illumina short reads and CCS subreads. De Bruijn graphs were built with Illumina data using k-mers of size 13, 15, 17, 19, 21, 31, 41, and 51 and guided by 462,447 CCS subreads (985 Mb, N50 = 2.1 kb) retrieved by unanimity v.3.0 [37]. On a further step, error-corrected CCS aligned by daligner [38] were input into daccord [39] to get consensus of CCSs. Those error-corrected consensus CCSs and 519 Mbp of genome data assembled by Celera were used as input into SSPACE-Long [40] to genome scaffolding. The final assembly resulted in a 517.5 Mb genome sequence (109,769 scaffolds with N50 of 6.54 kb).

#### 2.2.4. Assembly Polishing

QUIVER v.2.1 [41] from the BAM_Resequencing Beta.1 SMRT pipe v2.3.0 was used to provide SNPs and high-quality base calling for each scaffold.

#### 2.2.5. Assessment of Genome Assembly Quality

Genome assembly completeness was evaluated by BUSCO [42], using the metazoan lineage data resulting in scores to be comparable with other tunicate species.

### 2.3. Transcriptome Data Assembly

Illumina sequence data (PE reads of size 76 bp) were trimmed using BBtools [43]. After trimming a total of ∼2.6 Mbp comprising 55.1 millions of PE reads of size 50 bp and Phred ≥30 were input to perform a genome-guided Trinity *de novo* transcriptome assembly using Trinity v2.4.0 [44]. Reads were first aligned to the reassembled genome of *D. vexillum* with Gmap (Version 2019-06-10) [45] to get groups of overlapping reads into clusters used for further steps for the *de novo* transcriptome assembly. Finally, 39 Mbp comprising 90,938 transcripts were assembled and processed by TransDecoder [46] to find coding regions within transcripts.

### 2.4. Genome Annotation

Gene structure was predicted using Maker v.3.01.02 [47] in two rounds. First Maker annotation round consisted of runs of Augustus 3.3 [48] and RepeatMasker version open-4.0.5 [49] with *Ciona robusta* models and RepBase (RepBase20.03) [50]. This first draft annotation was further improved in a second round by incorporating transcripts, peptide and filtered RNAseq raw data previously used to assembly *D. vexillum* transcriptome; the gene finder SNAP [51] was trained for *D. vexillum* from results obtained in the first MAKER run.

Besides, Repeatmodeler [52] was used to construct our *de novo* repeat annotation library which was used in combination with RepBase (RepBase20.03) by RepeatMasker to assess for the total repetitive elements content of *D. vexillum*. Semi-HMM-based Nucleic Acid Parser (version 2006-07-28); GeneMark, GeneMark.hmm eukaryotic, version 3.54 [53]; and Nucleotide-Nucleotide BLAST 2.4.0+ [54] were used in the steps of Maker annotations. Finally, eggNOG v.5 was used to identify clusters of orthologous groups as described below.

Protein quality measures were calculated by collecting evidence derived from the following analysis: proteinortho v6.0.28 [55] orthologs detection to the solitary tunicate *C. robusta* non-*ab initio* proteins (http://ghost.zool.kyoto-u.ac.jp/datas/HT.KYGene.nonabmodels.protein.fasta.zip, accessed on 9 February 2021) reported in [12]. In addition, using blastp to the NCBI *non-redundant proteins* set to identify metazoa/non-metazoan homologs. Finally, results from a functional annotation method (as described in Section 2.10) where mapped to eggNOG v.5 [56] using eggNOG-Mapper v.2 [57]. Command line parameters used for this analysis are summarized in Section S12.

### 2.5. Identification of Contamination

A modification of the protocol described on [26] to detect possible contamination was performed (see details in 1: Section S5). A total of 4 scaffolds (∼18.65 kb) were removed from the original genome assembly, resulting in a final genome with: 109,769 scaffolds and 517.55 Mb size.

### 2.6. Annotation of Non-Coding RNAs

Annotated ncRNA candidates from the first assembly of *D. vexillum* were mapped in the new assembly as described in Section S6. At the same time, homology blastn and HMM strategies with their corresponding metazoan-specific CMs and default CMs evaluation have been applied following the methodology proposed in [26], to annotate candidates that have not been detected with the mapping strategy. The tRNAs genes were found using tRNAscan-SE v.2.0.3 with default parameters. A final check of candidates was performed to ensure that reported Rfam families contain at least one Metazoan sequence in their original seed alignment. These last step was performed to report possible false-positive families that could be retrieved applying the default Rfam models directly to the genome.

In order to annotate the position of *mature* sequences from miRNA candidates, MIRfix [58] was used. The miRBase (v.22) mature and hairpin sequences were used as initial sequence resource. Via RNAcentral database [59], the cross-link between miRBase and Rfam (v.14.1) was retrieved, and a list of Rfam families were classified as annotated in both databases. In this case, MIRFix corrected the *mature* position within the corresponding *hairpin* sequence. After that, the remaining *seed* sequences that have not been annotated in miRBase were included to be evaluated by the same methodology, but with the *mature* family-specific sequences. Final correction and annotation of those families, allowed the re-build of multiple sequence and structural alignments from the Rfam defined sequences, as a stockholm alignment. Given those results, the *D. vexillum* miRNA sequences annotated in this study, were processed as subject to annotate their *mature* sequences, based on previously detected matures in the Rfam families. At the end, positions of the most probable *mature* sequences and the corresponding alignments in stockholm format for each miRNA Rfam family were retrieved. Those genome annotations can be assessed via the described genome browser.

### 2.7. Computational Identification of miRNAs

Based on the previously corrected set of Rfam *seed* sequences, an evaluation of *D. vexillum* predicted miRNAs was performed using MIRfix [58]. Precursors that contain mature annotation and are supported by a correct structural alignment, were considered true candidates (for details see Section S6). To retrieve phylogenetic distribution of the Rfam sequences, taxonomic distribution (annotated as *kingdom*, *phylum*, and *subphylum*) was retrieved from NCBI Taxonomy Browser (https://www.ncbi.nlm.nih.gov/taxonomy, accessed on 13 February 2020) in the Rfam stockholm alignments. Details can be found in Figure S11.

### 2.8. Mitochondrial Genes

Mitochondrial complete genome from isolated clade A (NC_026107) and isolated clade B (KM259617.1) of *D. vexillum* were retrieved from GenBank as reported by [60]. Both sets of sequences where mapped with blastn against the new *D. vexillum* genome. The best candidates were retrieved adjusting identity ≤95%, E-value ≤0.001, and coverage 100% cut-offs. Final coordinates files are available in GFF3 format. Filtering of the intergenic coordinates was performed by a Perl script and this output was depicted with LuaT_E_X package pgfmolbio. Annotated Tunicata mitochondrial genomes were collected from NCBI. Multiple mitochondrial genome alignments were calculated using progressiveMauve [61] as referenced in Section S12.

### 2.9. Genome Size and GC Content Estimation

Genome sequences were retrieved from Ensembl v81: *Petromyzon marinus* v7.0, *Danio rerio* vZv9.73, and *Latimeria chalumnae* LatCha1. ANISEED [23]: *Molgula occidentalis* v1.0, *Molgula oculata* v1.0, *Molgula occulta* v1.0, *Botryllus schlosseri* v1.0, *Botrylloides leachii* v1.0, *Halocynthia roretzi* MTP2014, *C. robusta* KH, *Ciona savignyi*. NCBI: *Salpa thompsoni* v1.0, *Patiria miniata* v.2.0, *Strongylocentrotus purpuratus* Spur4.2, and *Saccoglossus kowalevskii* Skow_1.1. Other sources: *Oikopleura dioica* v3.0 (http://www.genoscope.cns.fr/externe/Download/Projets/Projet_HG/data/assembly/unmasked/, accessed on 26 September 2018). *Branchiostoma floridae* (http://genome.jgi-psf.org/Brafl1/Brafl1.download.html, accessed on 26 September 2018) v2.0 and *Branchiostoma belcheri* v.3.0 (http://mosas.sysu.edu.cn/genome/download_data.php, accessed on 26 September 2018). Genome parameters were calculated with bbmap [43] using the stats.sh script.

### 2.10. Functional Annotation of Protein Coding Genes

Protein fasta files were retrieved from Ensembl v81: *C. savignyi*, *P. marinus*, *L. chalumnae*; Aniseed [23]: *C. robusta*, *B. schlosseri*, *M. oculata*, *M. occidentalis*, and *B. leachii*. Proteins from *B. floridae* were retrieved in JGI [62] and for *O. dioica* from Oikoarrays [63].

Functional annotation from all retrieved species were assessed using eggNOG-Mapper v.2 [57], based on the database eggNOG v.5 [56] applying DIAMOND as referred on [64].

#### 2.10.1. Protein Enrichment Analysis

Enrichment analysis was calculated with goatools [65] taking as *background* group the complete set of proteins reported for studied chordata species and the *comparison* group, the list of proteins for each species. The *association* file between proteins and GO was generated based on eggNOG-Mapper results, all the command line methods are described in Section S12. Final results of enrichment were plotted using ggplot2, tidyverse [66,67] and grid [68] R packages. TreeMap plots were performed with REVIGO [69]. Calculated *p-values* from goatools were used as input data to REVIGO webserver against UniProt-to-GO database (ftp://ftp.ebi.ac.uk/pub/databases/GO/goa/UNIPROT/goa_uniprot_gcrp.gaf.gz, accessed on 14 April 2020) and SimRel as semantic similarity measure.

#### 2.10.2. Interaction Analysis of Proteins

Proteins with the same semantic terms in the REVIGO results were clustered and subject to a protein–protein interaction analysis using STRING (v.11) [70]. Proteins from *D. vexillum* were compared against the entire Chordata protein set. As *C. robusta* was the species with the largest number of recognizable homologs, this species was used as reference. Only connected nodes with “high” or “highest” confidence were analyzed and visualized.

#### 2.10.3. Annotation of *Homeobox* Proteins

A collection of reported *homeobox* proteins from human (of the family *Homeoboxes* (516) (https://www.genenames.org/cgi-bin/genegroup/download?id=516&type=branch, accessed on 10 October 2019) from HGNC database [71]), *C. robusta*, *C. savignyi* (both species from Ensembl v100 [72]), *B. leachii* [25], *H. roretzi* [73,74], and a variety of species from the HomeoDB [75] were retrieved from the corresponding references. This set was used to search along the annotated transcriptome and protein sequences from *D. vexillum* using tblastn, and blastp, respectively. The best candidates were obtained with an identity percent of ≥35, E-value $\leq 10^{-5}$ and a query coverage of 70%. For command line details refer to Section S12.

As a complement, pairwise genome alignments with the new assembly from *D. vexillum* and close species that reported annotations of *homeobox* genes: *B. floridae*, *B. leachii*, *B. schlosseri*, *C. savignyi*, *C. robusta*, *H. roretzi*, and *O. dioica*, were performed with LASTZ [76]. References from *homeobox* genes were obtained from Aniseed [23] using the Gene Builder with the term *hox*, except from *B. floridae* where updated annotations (for v.2) were searched and retrieved from LanceletDB [77]. Cross-matching of shared regions and reported genes and homology searches were performed to support the identification of *homeobox* candidates.

#### 2.10.4. Detection of Orthologous Proteins Involved in Skeletogenesis

We searched for RUNX, SOX, and Hh homologs in the output of eggNOG-Mapper for all studied chordate species. The corresponding orthology groups have the accession numbers: KOG3982, KOG0527, and KOG3638, respectively. Due to the lack of true RUNX orthologs on *D. vexillum*, we performed an additional analysis to confirm the presence of some homology signal. We retrieved the *RUNX* sequences reported on [78], from available 16 chordates from NCBI: AN08565.1, AAN08567.1, AAQ88389.1, AAS02047.1, AAS21356.1, BAA03485.1, BAF36001.1, BAF36011.1, EAX04278.1, EDL03777.1, EDL29993.1, ENSCINT00000004611.3, NP_001001890.1, NP_001092121.1, NP_004341.1 and NP_571678.1. Those sequences were searched with blastp in the proteome of *D. vexillum* and the following 10 species: *B. floridae*, *B. leachii*, *B. schlosseri*, *C. robusta*, *C. savignyi*, *M. oculata*, *M. occidentalis*, *O. dioica*, *P. marinus*, and *L. chalumnae*. On the other hand, the PFAM domain *Runt* (PF00853) was searched along all the reported proteomes of the described species using hmmscan (HMMER v.3.1b1) [79]. Filtering was based on the *gathering score* reported by PFAM and a low E-value <0.001.

#### 2.10.5. Gene Phylogenies

Phylogenetic analysis from mentioned proteins was performed on the set of orthologs from the described target species and their corresponding orthologous sequences that have been obtained by the eggNOG-Mapper analysis. As an outgroup, we obtained from NCBI, the following sequences for *RUNX*: NP_999779.1 and XP_781626.2; for *Hh*: FBpp0121221, KDR14772, and XP_008546836.1.

For the analysis of RUNX, we included the reported sequences of lamprey (*Lethenteron camtschaticum*) [80], annotated in NCBI with the following accession numbers: AJM44878.1, AJM44883.1, and AJM44886.1. The complete phylogenetic analyses were performed by ETE 3 Toolkit [81], using Maximum Likelihood (ML) with the JTT+G+I substitution model and a bootstrapping of 100. Specific command line is described in Section S12. Gene IDs were replaced by “human-readable” names in Figure S5. A version with the database IDs is provided in Supplementary File 2.

### 2.11. Genome Browser Construction

GFF3 annotation files for coding genes, ncRNAs, and mtDNA were processed using MakeHub [82] as preprocessing step to generate the input files of the hub. The input files were used to create a genome Hub hosted on the UCSC hub site [83].

## 3. Results

### 3.1. Assembly of the *D. vexillum* Genome

Using the modified preprocessing and assembly procedure described in detail in the Method section above, an improved assembly of the *D. vexillum* genome was obtained by integrating PacBio and Illumina sequencing. The new, scaffold-level assembly comprises approximately 517.55 Mb. This amounts to a reduction in the number of genome fragments by a factor of ∼8× and a corresponding increase in the N50 length from 918 bp to ∼6.5 Kb. The new assembly also decreases the estimated genome size by about 25 Mb. While only about 15% of the contigs in our previous study [26] were longer than 1 kb, this threshold is now exceeded by almost 96% of the scaffolds in the new assembly and thus allows at least a comprehensive gene-level analysis. The newly analyzed nucleotide composition was consistent with our previous study [26].

The quality of the assembly is limited by two major issues. (1) The genetic heterogeneity of the colony and therefore pronounced differences in haplotypes pose a direct problem for mapping steps in classic assemblers. A pooled sequencing protocol, as was chosen for this project, is therefore suboptimal, see Section S4. (2) The PacBio reads fall short of the expected length distribution due to high levels of gRNA degradation. We investigated several alternative assembly strategies to rule out problems with the computational approach. For details we refer to Section S3.

**Figure 2:**
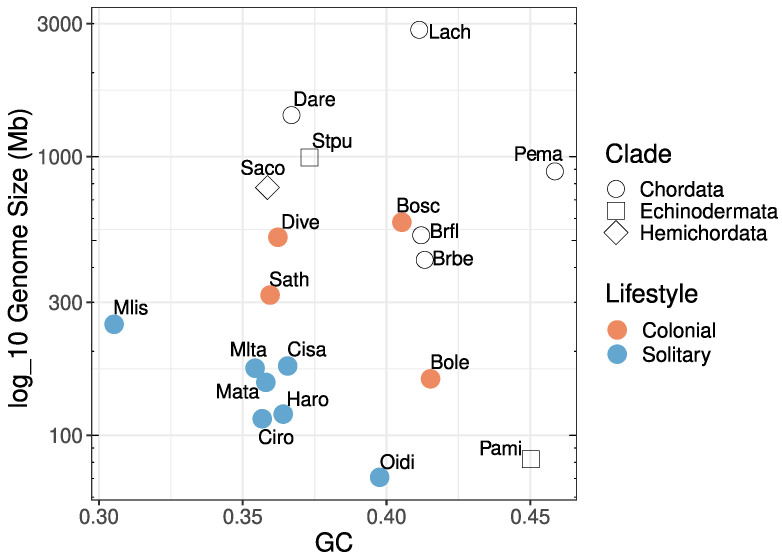
Distribution of estimated genome size and GC content of Deuterostome taxa. We included Hemichordata (*S. kowalevskii*, **Saco**), Echinodermata (*Patiria miniata* **Pami** and *S. purpuratus* **Stpu**) and Chordata species. Filled circles are tunicates and their lifestyle, colonial or solitary, is highlighted in orange or blue accordingly. Species labels: *Branchiostoma floridae* (**Brfl**), *Branchiostoma belcheri* (**Brbe**), *Oikopleura dioica* (**Oidi**), *Molgula occidentalis* (**Mlis**), *Molgula oculata* (**Mata**), *Molgula occulta* (**Mlta**), *Botryllus schlosseri* (**Bosc**), *Halocynthia roretzi* (**Haro**), *S. thompsoni* (**Sath**), *Botrylloides leachii* (**Bole**), *D. vexillum* (**Dive**), *Ciona robusta* (**Ciro**), *Ciona savignyi* (**Cisa**), *Petromyzon marinus* (**Pema**), *D. rerio* (**Dare**) and *Latimeria chalumnae* (**Lach**).

Tunicates have a GC content ≤43%, with the lowest values reported for solitary ascidians, in particular molgulids. *Didemnum vexillum* is similar in GC content to the salp *Salpa thompsoni*, most solitary ascidian species, zebrafish *Danio rerio*, and the two ambulacrarian outgroup species (*Strongylocentrotus purpuratus* and *Saccoglossus kowalevskii*). Although we do not find a clear relationship between GC content and genome size in the deuterostomes, when we compare both factors (i.e., genome size and GC content) together, there is a tendency for tunicates to have both lower GC content and smaller genome sizes when compared to other deuterostomes, and other chordates in particular. Moreover, within tunicates, solitary species show even lower GC content and genome size compared to colonial species (Figure 2). It remains an open question what the biological consequences of this trend are for tunicates in general and colonial tunicates in particular.

### 3.2. Transcriptome Sequencing and Assembly

In order to support the annotation of the Sea vomit genome, the transcriptome was assembled from RNA-seq reads. Trinity assembled a total of 55.1 million paired-end Illumina reads into 90,938 transcripts. After two training rounds of Maker, only 64,424 transcripts were annotated, with a median contig length of 375 nt with a positive skewed, long-tailed distribution. There are transcripts with a length >10 kb, both corresponding to the uncharacterized proteins Dex_pep14095 and Dvex_pep554 (as shown in Tables S4 and S5). Both of which have homologs in *C. robusta*, containing the TILa (PF12714) and von Willebrand factor type C protein domains (PF00093).

### 3.3. Genome Annotation

#### 3.3.1. Detection and Analysis of Repetitive Regions

To identify regions prone to have repetitive elements, a combined strategy using RepeatModeler and RepeatMasker was used to generate a *de novo* library. Additionally, including also the reported repeat’s library from *C. robusta*, the *D. vexillum* genome was soft-masked, as explained in Methods. Approximately 300.66 Mb, i.e., 57.89% of the assembled *D. vexillum* genome, consists of repetitive elements (see Section S7). Most of the repetitive elements are interspersed repeats (56.96%) as well as retroelements (12.86%), DNA transposons (7.65%), leaving about 100 Mb of the repeats as unclassified elements (35.96%). This is similar to the repeat content of *B. schlosseri* (∼59.85%) [25]. The most abundant family of repeats in *D. vexillum* are 100,404 copies of SINE/tRNA-Lys, a class of repetitive elements that have not been reported for other tunicate species. The other highly abundant families (i.e., LINE/L2 and DNA/hAT-Charlie) are also prevalent in other tunicates, see Table S9 for details.

#### 3.3.2. Annotation of Protein-Coding Genes

The computational annotation pipeline based on Maker and Augustus identified 62,194 putative coding genes accounting for 64,424 distinct protein products (Table 1). Approximately 97.5% of coding genes have 0.97 kb in median, generate only one transcript and thus a single predicted protein product. Those genes that reported more than one transcript have minimum and maximum median sizes of 0.44 to 7.07 kb, respectively (Table S4). The largest annotated gene, *Divexi.CG.Dive2019.scaffold1-size56789.g1453*, has a size of 33.74 kb and comprises a single transcript product, which accounts for a protein with domains as Laminin N-terminal (PF00055), Laminin EGF (PF00053), and Carbohydrate-Binding Module 6 (PF03422). At the same time, it presented high homology to the *C. robusta* laminin alpha 5 subunit protein XP_026696566.1. The gene with the largest number of transcripts is a homolog of *Dynein heavy chain* proteins. It covers 16 exons in a region of only 7.07 kb and produces 10 observed isoforms, see Figure S6 and Table S5.

**Figure 3:**
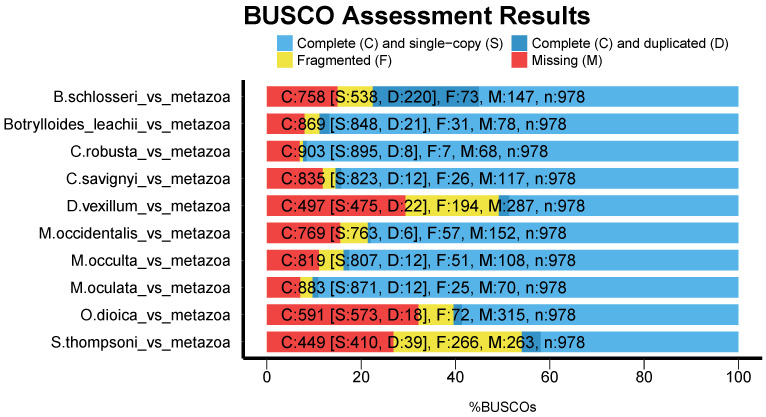
Completeness of tunicate genomes assessed by BUSCO [42] in comparison to metazoan orthologs.

In order to assess the quality of both the genome assembly and the predicted gene set, we used BUSCO to compare them to metazoan orthologous genes (Figure 3). For the *D. vexillum* genome, from the 978 orthologs, 50.8% were found complete. Overall, the BUSCO results are comparable to other, published tunicate genomes (Figure 3), indicating that current assembly of *D. vexillum* is comparable to the *S. thompsoni* assembly in terms of completeness and annotation. In general terms, most of the reported tunicate genomes displayed ≥75.4% of complete BUSCO orthologs.

In order to assess the quality of protein-coding annotations, we compared the predicted protein products in the *D. vexillum* assembly with three sources of annotated proteins: (1) the best-annotated tunicate, *C. robusta*, which features 14,072 proteins; (2) the RefSeq non-redundant protein database from NCBI; and (3) the pre-clustered sets of orthologs obtained from the eggNOG database (described in more detail in Section 3.3.6 below). We obtained plausible homologs in other metazoans for 26,024 (about 40.4%) of the putative *D. vexillum* proteins identified by Maker/Augustus. A small fraction, ∼1.6%, are similar only to non-metazoan proteins (most of them from bacteria and/or fungi). We interpret these as possible contaminations in the assembly, which should be interpreted with case.

The remaining 37,392 putative proteins have no recognizable homologs. For more than one-third of these, none or only an incomplete 3’ or 5’ UTR was reported, and only approximately 16% of these protein models have complete UTRs and at least some experimental support (see details on Section S10). We argue that most of them are computational artifacts and even the set supported by transcripts may largely consist of long non-coding RNAs rather than protein-coding genes. The annotation tracks provided in the accompanying genome browser distinguish between ORFs with metazoan homologs, potential contaminations, and likely false positives from the computational protein annotation pipeline.

#### 3.3.3. Homeobox Transcription Factors

**Figure 4:**
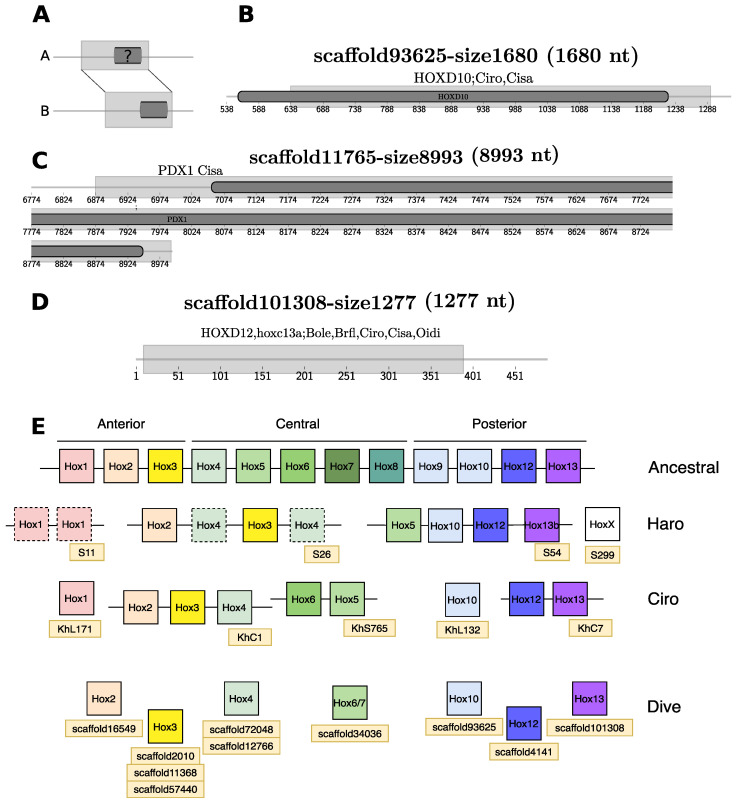
Detection of *Homeobox* genes on *D. vexillum*. (**A**) Model of detection, a shared region between genomes *A* and *B* is detected and referenced as gray boxes. Correspondence is denoted by dotted lines between genomes. The dark gray box in genome *B* represents an annotated gene whereas the dark grey box mark represents the putative orthologous region. (**B–D**) Examples of putative orthologous Hox gene assignment in *D. vexillum*. Specific details are explained in the main text. (**E**) A summary of the complete *Homeobox* genes annotation in *D. vexillum* (**Dive**) in comparison to reported genes on *C. robusta* (**Ciro**) and *H. roretzi* (**Haro**). Genomic locations were retrieved from ANISEED, Hox cluster of the chordate ancestor is depicted [73,74]. Uncertain positions of some genes are represented as a dotted box, e.g., *Hox1* and *Hox4* in *H. roretzi*. For specific genome coordinates see Table S6.

In this initial annotation, we specifically searched for *homeobox* transcription factors using a combined blastp/tblastn strategy (see Methods) that identified 48 coding sequences with their corresponding number of genes located in 47 scaffolds. The most frequent found proteins are homologs from the families: *ZEB2*, *LHX2*, and *Irx* transcription factors. In an alternative approach we used the genome-wide alignments to compare existing annotations of homeobox genes in six tunicate and one cephalochordate genomes to our *D. vexillum* assembly (see Methods). Only one of the 48 homeobox *loci* had annotated homologs in four of the six query species, which corresponds to a *Hox2* gene, located on the *scaffold16549-size8805*. Several other Hox genes, however, were not recognized by the default homology annotation pipeline because of incomplete overlaps, and in some cases, no gene was annotated for *D. vexillum* (Figure 4).

By comparison with *H. roretzi* and *Ciona* spp., we expected to find three anterior, three middle-group, and three posterior Hox genes as in other tunicate genomes [73,74]. Based on the data outline above and a more detailed manual search with genome alignments as support, we found evidence for two anterior genes (*Hox2* and *Hox3*), two central genes (*Hox4* and *Hox6/7*-like), and the three expected posterior genes, as referred on Figure 4. What the consequences of the presumable absence of *Hox1* and *Hox5* are for *D. vexillum* remains to be studied. The assembly of the HOX gene region unfortunately is too fragmented to conclusively rule out the presence of *Hox1* and *Hox5* or to provide any linkage information of the reported Hox genes.

#### 3.3.4. Annotation of Non-Coding RNAs

Noncoding RNAs were annotated using a homology-based strategy combining blastn searches, HMM profiles, and covariance models (CMs) as described in [26] with some modifications detailed in Methods. Not counting tRNAs, we identified 2153 ncRNA *loci* corresponding to 271 distinct ncRNA families. A search with tRNAscan-SE resulted in 18,343 predicted loci, including pseudogenes and undetermined isotype candidates. In addition, we mapped the 206 families of ncRNAs identified in a preliminary draft of the *D. vexillum* genome [26] to the current assembly (see Methods and Section S6). As in other genomes, in particular the pol-III transcribed RNAs including 5S rRNA, tRNAs, and U6 RNA, as well as the snRNAs transcribed by pol-II appear in multiples copies [84]. The data are summarized in Table 2. While most ncRNAs were visible in the automatized annotation pipeline, several additional ncRNAs could be added by manual curation only. RFAM IDs for the RNA families mentioned below can be found in Supplementary File 2.

**Transfer RNAs.** We found 2724 tRNAs and 15,619 tRNA pseudogenes or with undetermined isotype (23). The most abundant tRNA is ${\mathrm{tRNA}}_{\mathrm{Thr}}$ with 1395 copies, while only a single copy of ${\mathrm{tRNA}}_{\mathrm{SeC}}$ was observed. Surprisingly, tRNAscan-SE reports numerous suppressor tRNAs: 153 (${\mathrm{tRNA}}_{\mathrm{Suppressor}-\mathrm{TCA}}$: 145, ${\mathrm{tRNA}}_{\mathrm{Suppressor}-\mathrm{TTA}}$: 7, and ${\mathrm{tRNA}}_{\mathrm{Suppressor}-\mathrm{CTA}}$: 1). Detailed information on the tRNA annotation is compiled in Figures S7 and S8.

**Ribosomal RNAs.** As in most eukaryotes, the small and large subunit (SSU 18S and LSU 28S) rRNAs are organized in repetitive units of the rRNA operon. It also contains the 5.8S rRNAs. In this case, *D. vexillum* reported 6 clusters of rRNAs: two clusters are composed of repetitions of 5S rRNA (*scaffold1545-size16374* and *scaffold22447-size6833*), two clusters contain SSU 18S, 5.8S, and LSU 28S rRNA elements within (*scaffold4839-size12187* and *scaffold9164-size12300*), and one cluster contains repetitions of 5.8 rRNAs with a locus of LSU 28S rRNA (*scaffold4349-size12561*). At the same time for the subunit 5S rRNA 71 loci were detected and from them 52 are located on the *scaffoldUncertain*. For the other rRNAs elements, in total were found 6 5.8S, 3 SSU, and 4 LSU rRNAs.

**Spliceosomal RNAs.** All RNA components of the spliceosome machinery were found in the new genome assembly. As usual, the snRNAs of the major spliceosome appear in multiple copies U6 (46), U5 (9), U1 (21), U2 (27), U4 (3). Among the snRNAs of the minor spliceosome, U12 appear once, while there are 2 loci coding for U4atac, U6atac, and 4 U11 genes.

**Other small nuclear RNAs.** We identified the expected genes for the RNA component of the signal recognition particle as well as the RNase P RNA, RNase MRP RNA, and 7SK RNA. No homologs were found for the telomerase RNA, U7 snRNA, vault RNA, and Y RNAs, although their presence in the genome is expected. These groups are notoriously difficult to be detected by homology search without the benefit of known homologs in closely related species [85]. A thorough search along reported Tunicata genomes successfully reported vault snRNA *loci*, except for *D. vexillum*, other families were not detected, indicating that specific CMs should be redefined with a broad set of sequences to improve the annotation from those families on *D. vexillum* and another tunicate species (Table S8).

**MicroRNAs.** The miRNA annotation pipeline, described in the Methods section, identified 2065 *loci* encoding members of 248 distinct miRNA families. An additional 20 *loci*, which harbor two additional families, correspond to previously reported miRNAs [26] which were successfully mapped into the new assembly. To avoid the annotation of false positives due to the modification of the threshold values (see Figure S16), the position of the *mature* sequence was evaluated using MIRFix [58] which used both, the RFAM database for the miRNA families alignments and miRBase as source for the annotated *mature* sequences (as explained in more detail in Methods and Figure S9). As a result, the definition of a true miRNA candidate relies not only on the homology results given by the sequence/secondary structure comparison, but also in the annotation of their *mature* sequence. In addition, we also require miRNA-specific features, such as a conserved position of the mature products within the defined miRNA family. To this end, candidates that reported homologous *mature* regions were compared against their corrected stockholm alignments, by the calculation of the *tree edit distance* between generated consensus secondary structures, as described on Section S6.

This way, a number of 1582 *loci* were reported, from which 1394 fulfill all the designed filters and reported a set of *mature* sequences harbored at the predicted hairpin structure, the other 188 have broken the conservation block in the defined family alignment, despite having shown a high conservation at hairpin level. Taking into account those detected miRNAs with *mature* annotation, the distribution of *loci* shows that 75% of miRNA families have less than 6 *loci*. The corresponding 25% of miRNA families have a higher median of ∼11.5 *loci*. Within these miRNA families, mir-544 (65), mir-578 (70), and mir-944 (97), had the highest number of *loci*.

We also analyzed the phylogenetic distribution of the miRNAs in the Rfam *seed* alignment, the corresponding species were retrieved along with their annotated *kingdom*, *phylum* and *subphylum*, as described in Methods. The annotated miRNA families and their *loci* in *D. vexillum* were compared as shown in Figure S11. We found 18 miRNA families that were represented in more than 2 *phyla*: **mir-124**, mir-598, mir-7, **let-7**,**mir-1, mir-133**, mir-33, *lin-4*, **mir-137**, **mir-153**, mir-2, **mir-31**, mir-449, **mir-183**, **mir-190, mir-210, mir-219**, and **mir-8**. Families highlighted in bold showed a conserved structure (panel labeled as *VALID_STR*), even when the *D. vexillum* sequences were included into the alignment. In this analysis, we uncovered two additional families: **ciona-mir-92** (RF01117) and **mir-281** (RF00967) to the previously reported **mir-1497** (RF00953) [20], candidate in *D. vexillum*. In contrast, a subset of 13 miRNA family candidates did not fit into the corrected stockholm alignment (classified as *NO_VALID_STR*), despite our previous homology validation.

In a previous study of the miRNA complement in the solitary species *H. roretzi* [20] a more extensive list of tunicate-specific miRNAs was reported (21). From these only one (**mir-1497**, (RF00953)), was detected in our study because of the corresponding covariance model used to validate their secondary structure. From the *conserved* families of miRNAs in Metazoa (25) we identified 21 in *D. vexillum*. Other families, including **mir-9**, **mir-182**, **mir-184**, **mir-200**, and **mir-218**, were not found. These families (except **mir-200**) were also found to be absent in other tunicates such as *C. savignyi* and *O. dioica* [20]. Absence of these families was also reported in a preliminary analysis along bilaterian species [86].

From our previously reported set of miRNAs [26], 16 families were detected only in *D. vexillum* and not in other tunicates. From this set, 10 families were annotated in our new assembly and four were discarded because their mature sequences could not be annotated (**mir-130**, **mir-460**, **mir-185**, and **mir-233**), one does not have a covariance model (**mir-4068**), and another was not found in the new assembly (**mir-9**). From the set of shared families in colonial tunicates, all were annotated and validated by our strategy, except **mir-340** (RF00761). The latter showed a good homology but did not pass the conditions of the current structural alignment strategy, which used only vertebrate sequences to assign homology. In this study, we report **mir-31**, as the sole miRNA candidate that passed all our present filtering criteria to be exclusively found in solitary ascidian species. We also excluded 502 candidates based on the lack of conserved *mature* sequences inside the hairpins.

**Small Nucleolar RNAs.** Conserved snoRNA families were detected by the automatized homology-search strategy. We found 3 U3, 2 copies for SNORD14, SNORD18, snoZ39, and SNORA36, as well as a single copy of SNORD29, SNORD33, SNORD35, SNORD36, SNORD52, SNORD63, and SNORD83.

**LncRNAs and other structured RNA elements.** Two structured lncRNAs were found, corresponding to the *Rhabdomyosarcoma 2-associated transcript conserved region*: RMST8 (1) and RMST 9 (7), the latter one has already been previously annotated [26]. As a result of the iteration and re-building of the correspondent CM with newly detected tunicate sequences, (see Figure S12) we now report the occurrence of the complete RMST family in deuterostomes. RMST 8 and 9 were detected in all deuterostomes. We found two additional RMST families (RMST 6 and 7) in the coelacanth suggesting an initial expansion in the ancestor of lobe finned fishes (Sarcopterygii). The complete set of RMST 1, 2, 3, 4, 5, 6, 7, and 10 were detected in mammals. Because of their relevance in neural development [87], it would be interesting to study the evolution of RMSTs in the tetrapods, and the ancestral role of RMST 8 and 9 in the deuterostomes, the tetrapods and mammals.

Finally, by using a specific search with HMMs and CMs we identified 326 loci carrying the Histone 3’ UTR stem-loop, 6 instances of the Potassium channel RNA editing signal, one for the Iron response element II and 9 loci for Hammerhead ribozyme (type I).

#### 3.3.5. Mitochondrial Genome

The mitochondrial genome of *D. vexillum* maps to a single scaffold *scaffold1656-size16126* and very closely matches the two previously reported mitogenomic sequences [60], known as Clade A and Clade B. The mt-LSU is 99.9% identical to Clade A, and diverges about 3.6% from Clade B, confirming that the collected organisms belongs to clade A, see also [26]. Mapping the currently reported elements from mtDNA, resulted in the gene order depicted on Figure S14. In this case, intergenic distances were reduced, but the size and the order of the genes in the new assembly were conserved. The 37 expected elements of mtDNA were mapped to the new assembly. The gene order of the mitogenome matches that of clade A but differs from other tunicate species, as shown in the multiple alignment of the mitogenomes in Figure S15.

#### 3.3.6. Functional Annotation and Comparison of Proteins across the Tunicates

To obtain functional annotations for the predicted *D. vexillum* proteins we used the pre-clustered orthology groups from the eggNOG database [56] together with the protein annotation of eleven chordates (see Methods). We obtained 8349 orthology groups of which 6279 were represented in at least two of the chordates included in our reference set. Figure S28A, shows that 57.1% (4584) of the orthologs were shared with at least one sequence of each of the major branches of the chordates (Cephalochordata, Tunicata, and Vertebrata). Only 3.63% of orthologs were shared exclusively with at least two species of tunicates, while 15.81% of orthologs were shared only with vertebrates. We note that the quantitative analysis of protein-coding genes may be confounded by the fragmentation of the assembly and thus should be considered with caution.

Along all detected sets of orthologs which are shared exclusively among two or more tunicates (292), 5 were found present in all Tunicata species. From this subset, the *lytic polysaccharide monooxygenase* (ENOG5028N9R) was involved in cellulose fibrillation and degradation. The other orthologs were unknown proteins with sulfotransferase family domains (ENOG502CNPV, ENOG502CXMB), pleckstrin homology domains (ENOG502EA0P), or transmembrane domains with unknown function (ENOG502EQW0).

To reach a more universal understanding of ortholog proteins during the evolution of coloniality, we need to better characterize and assign cellular functions to the conserved proteins found in colonial tunicates that evolved from independent events of coloniality. The mentioned groups of proteins present in *D. vexillum* and the two botryllids (described in more detail in Section S11.1.1) will provide a starting point to address their biological roles for ascidian colonies.

In spite of an absence of 1737 orthology groups of predicted proteins in the *D. vexillum* genome, most of these orthologs became detectable when the Chordata group were analyzed (79.2%). Moreover, in Olfactores and Tunicata (include *D. vexillum*), we found 12.7% and 8.1% of orphan genes, respectively. Although we report the functional profile of orphan genes in *D. vexillum*, which represent the majority of the orthologs recovered, we were not able to uncover a clear functional annotation for many of these genes (see Table S23 for details).

**Figure 5:**
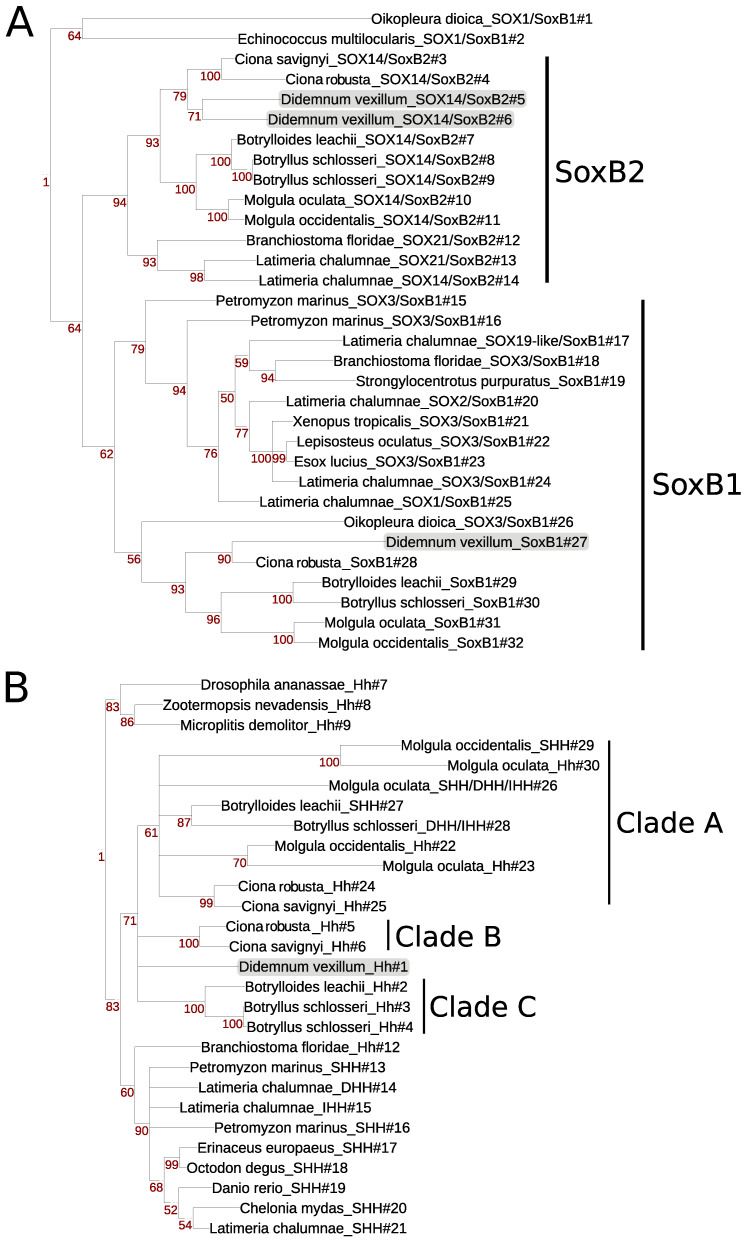
Phylogenetic analysis of skeletogenesis proteins found in *D. vexillum*. (**A**) SoxB1/B2 family, (**B**) Hh family. The sea vomit is highlighted in gray. A tree of the complete SOX family can be found in Figure S25. Trees were built using Maximum Likelihood (ML) with the JTT+G+I substitution model generating 100 bootstrap replicates.

Despite the difficulties in the assignment of ortholog candidates across all genome datasets, comparisons against clustered groups allowed us to detect and annotate orthologs in the *D. vexillum* genome. Because the Didemnidae can mineralize calcium to form spicules in their tunics, we decided to search for key proteins involved in skeletogenesis [88]: *Sox*, *Hedgehog* (Hh), and *RUNX*, which corresponded to the ortholog groups: *KOG0527* (SOX), *KOG3638* (Hh), and *KOG3982* (RUNX) on the eggNOG database. Gene phylogenies for these ortholog groups (including the chordate sequences used as reference and the orthologs annotated in the eggNOG database) are shown in Figure 5. In *D. vexillum*, we found seven members of the SOX family belonging to SoxB1, SoxB2, SoxC, SoxD and SoxE subgroups as defined in [89]. Overall, we found two paralogs for the SOXC (*SOX4/SoxC#32* and *SOX4/SoxC#33*) and SoxB2 (*SOX14/SoxB2#5* and *SOX14/SoxB2#6*) in our annotation of the *D. vexillum* genome, see Figure 5A and Figure S25 for the complete tree.

All tunicates except *O. dioica* reported members of the Hh families (Figure 5B). The basal Hh family, previously reported in Ciona [90] and in amphioxus [91], was detected in all ascidians. In the vertebrates, we confirmed the presence of the three Hh genes: Desert (DHh), Indian (IHh), and Sonic-hedgehog (SHh) [91,92]. In ascidians, we found several clades of Hh genes. There are at least three Hh families in the ascidians: Hh clade A (with medium bootstrap support of 61), Hh clade B (with full bootstrap support in *Ciona*) and Hh clade C (with full bootstrap support in the botryllids). The *D. vexillum* Hh does not group with any of the other clades. Our analysis supports an independent diversification of the Hh family in ascidians.

We did not find the key regulators of skeletogenesis RUNX-related transcription factor (RUNX) proteins in *D. vexillum*. This does not necessarily indicate a true loss, however, because in a detailed domain-based homology search (data not shown), we found parts of the *Runt* domain (PF00853) among 15 proteins from *D. vexillum*, albeit with truncated sequences. The phylogenetic distribution of the orthologs found (Figure S24), shows a defined clade of tunicate sequences that belong to the ancestral RUNX family, which has been detected in this study in amphioxus and is known to be expressed in *Ciona* and *Oikopleura* [80]. This suggests that RUNX proteins may not be truly absent in *D. vexillum*. We note in passing that the RUNX family has undergone additional duplications in the lampreys (Figure S24).

Ortholog groups determined by the eggNOG database were used to transfer the annotation to the corresponding orthologs in *D. vexillum*. General ontology terms (e.g., cellular, metabolic, multi-organismal processes, reproductive processes, regulation, and locomotion) were commonly annotated for *D. vexillum* proteins (Figure S26). Enrichment analysis with REVIGO [69] based on the frequencies of ontology terms (see Methods and Section S11.1.1 for details) detected seven distinct overrepresented semantic clusters in *D. vexillum*: Positive regulation of phospatidylinositol 3-kinase signaling, tRNA catabolism, secondary metabolism, chaperone-mediated protein folding, protein folding, protein autophosphorylation, and phosphorus metabolism, see Figure S28B.

A detailed annotation of the *D. vexillum* genome comparing the GO assignments from selected chordates genomes is provided in Figures S29 and S30. We found a total of 237 tunicate-specific enriched GO terms, when compared to the annotations in *B. floridae*, *P. marinus* and *L. chalumnae*. All tunicates, except *O. dioica* shared 8 assignments. Where related terms were found, we indicate these relationships (→ “is a”, ↦ “part of”) as follows: Oogenesis (GO:0048477) → Germ cell development (GO:0007281) ↦ Gamete generation (GO:0007276) (← Female gamete generation (GO:0007292)), cellular process involved in reproduction in multicellular organism (GO:0022412) →,↦ Multicellular organismal reproductive process (GO:0048609) ↦ Multicellular organism reproduction (GO:0032504) → Reproduction (GO:0000003).

**Figure 6:**
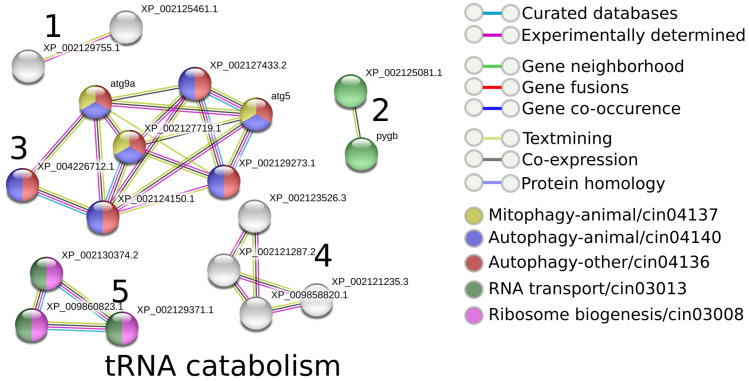
Functional interaction of homologous proteins in *C. robusta* which have shown homology with the functionally annotated proteins on *D. vexillum* with *tRNA catabolism* processes. Nodes correspond to single, protein-coding loci. Edges do not represent physically binding, but functional association determined by STRING [70]. The legend was obtained and modified from STRING web server (https://string-db.org/, accessed on 11 February 2020).

Based on the previously described semantic clusters, the functional interactions of involved *D. vexillum* proteins were inferred using STRING (v.11) [70], comparing them with their homologous proteins annotated in *C. robusta*. As an example, Figure 6 shows the annotations for *C. robusta* that have been detected as homologs of the proteins in *D. vexillum* involved in tRNA catabolism processes. As a result, it was possible to detect 5 protein clusters, each one with a specific interaction, as follows: cluster 3 was related to the autophagy pathways (KEGG pathways cin04136, cin04140, and cin04137), while clusters 2 and 5, are involved to the ribosome biogenesis and RNA transport pathways (cin03008, cin03013), respectively. Clusters 1 and 4 contain proteins without any clear association. For the other ontology clusters (Figure S26) we carried out the same analysis. As expected, for the proteins involved in protein folding, the functional annotation pointed out processes related with the endoplasmic reticulum. Regarding the pathways of secondary metabolism, we detected processes involved in starch, sucrose, and porphyrin metabolism, as well in chlorophyll metabolic pathways. In more detail, 3847 proteins are involved in the positive regulation of phospatidylinositol 3-kinase signaling, and in processes such as: endocytosis (cin04144), autophagy (cin04140), mTOR, FoXO, Wnt, and Inositol signaling pathways (cin04150, cin04068, cin04310 and cin00562) and RNA transport (cin03013). In addition, 1056 and 1053 proteins were found related to phosphorus metabolism and protein autophosphorylation, respectively. These detected proteins reported the same interactions in: metabolic pathways (cin01100), Inositol phospate metabolism (cin00562), phospatidylinositol signaling (cin04070), FoxO signaling (cin04068), purine metabolism (cin00230) and autophagy (cin04140) (see Figure S27).

In summary, we were able to infer several candidate functional networks on the basis of the semantic clusters detected in *D. vexillum* with the help of homologous proteins from the solitary tunicate *C. robusta*.

### 3.4. Genome Browser and Analysis of Genomic Coordinates

We provide a new genome resource: http://tunicatadvexillum.bioinf.uni-leipzig.de/ (accessed on 3 December 2021), derived from our new *D. vexillum* assembly. This resource is linked to the UCSC genome browser hub [83] as described in Methods. Genome coordinates for ncRNAs and annotated genes were concatenated, sorted and intersected by incrementing the starting position for each scaffold and by reporting the genome coordinates. We labeled the ncRNAs as suggested in the guidelines for tunicate elements [93]. Accordingly, we found that a total number of 2378 genes have a ncRNA nearby or within their gene structure. These corresponded to 1832 ncRNAs, where 53.93% were tRNAs, 36.14% (183) miRNA families, 6.66% (3) *cis* regulatory RNAs and 0.27% miscellaneous RNAs (1) and ribozymes (2). Other housekeeping RNAs summed up 3%, including rRNAs (3 families), snoRNAs (10) and snRNAs (6).

Protein-coding gene annotation tracks were reported with their corresponding evidence generated by Maker and the quality assessment described in Section 3.3.2 (metazoan homologs, potential contaminations, and likely false positives).

## 4. Discussion

The genome assembly reported here pertains to a specimen of *Didemnum vexillum* Clade A, determined by the mt-LSU RNA. *D. vexillum* has a similar genome size and GC content as other deuterostome genomes, including ten tunicate genomes. Among tunicates, solitary organisms appear to have smaller genomes (≤250 Mb) than colonial ones (with range from 160 to 723 Mb). The *D. vexillum* genome thus appears in the typical size range for colonial tunicates, and in terms of its size, it is comparable to the amphioxus genome (Figure 2).

At the same time, the contiguity of the assembly still falls short of those available for other ascidians. Despite considerable efforts, a partial degradation of the genomic DNA detected in all field samples, presumably due to the unusually acidic milieu of the tunic bladder cells (restricted to some groups of ascidians, including the Didemnidae). The bulk of their cytoplasm comprises a large vacuole containing sulfuric acid, which accounts for a tunic pH < 3.0 in didemnids [94] that may be involved in chemical defense. In contrast, tunic pH > 6.0 was measured for *Perophora* and *Clavelina* species. The acidic pH may account for the observed gDNA degradation, possibly due to increased deamination rates [95,96]. The partial degradation of gDNA is a confounding factor for genome assembly, particularly limiting the achievable PacBio read lengths. As a consequence, to avoid DNA shearing during extraction for long read sequencing in this species, extraction methods for complex genomes should be considered, including extraction methods based on pulsed field gradient gel electrophoresis [97], or low-melting agarose microbeads or plugs, as well as other agarose based methods used previously for plant tissues and cells for shearing avoidance [98,99]. In addition, long-term EtOH storage of *D. vexillum* tissues should be avoided, and tissues should be deep-frozen with liquid nitrogen immediately after collection. Although we believe that the latter alone may not resolve the problem, it certainly provides an additional step of caution for extractions on this species.

The natural genetic diversity of *D. vexillum*, furthermore, is too large for standard genome assembly tools to produce satisfactory assemblies from pooled sequencing of multiple individuals. We therefore resorted to a strategy that reduces the impact of variation, possibly at the expense of contiguity. This genetic diversity is likely associated with *chimerism* of the sampled colony, a phenomenon reported both for *D. vexillum* [100,101,102] and other colonial tunicates [103]. Chimeric colonies appear to be a natural strategy to potentiate the invasiveness behavior, e.g., enhancing the colony survival having multiple genotypes inside the colony that would respond to a broader set of environmental conditions [101].

As a consequence, the assembly is far from perfect. Its contiguity is sufficient to provide exome-level information supporting detailed insights into the gene content of *D. vexillum*. It can be used for phylogenetic purposes, to study the gene structure of the majority of the coding genes, or the evolution of non-coding RNAs. It is insufficient, however, for investigations that involve large-scale synteny, e.g. an assessment of genome rearrangements, and it likely does not represent accurate copy numbers of repetitive elements.

The construction of a reference genome for *D. vexillum* that is on par with better understood tunicates such as *Ciona robusta* will mostly likely require the creation of an inbred line, as has been the case with other tunicate assemblies [12]. The high level of diversity observed here may also help to shed light on the fast spread and adaptation of *D. vexillum* to diverse biomes around the globe. It is reminiscent of the increased mutation rate observed for *C. robusta* which is linked to high diversity and adaptive evolution [104].

Functional annotation of the predicted *D. vexillum* proteome by comparison with 11 chordates resulted in 8349 orthology groups. The vast majority is shared among chordates. We identified 292 orthology groups in tunicates only (present in more than one tunicate). Among them five functional groups shared by all tunicates, including *lytic polysaccharide monooxygenase and cellulose-degrading processes* (ENOG5028N9R). Other shared orthology groups did not have a specific annotation, however in some cases protein domains (e.g., *sulfotransferase and pleckstrin families* and some transmembrane domains) were recognizable. From all the available chordate orthology groups, 1737 groups were not recovered in our *D. vexillum* assembly. Most notably, we did not find any member of the *RUNX* family, which correspond to key regulators of skeletogenesis together with *HH* and *SOX* family members. We observed that tunicates, except *Oikopleura dioica*, showed a tunicate specific expansion of Hh members. We found seven members of the *SOX* family. A phylogenetic analysis revealed duplication events for SoxC and SoxB2 in *D. vexillum*. We also identified seven of nine tunicate *homeobox* transcription factors of HOX family, the contiguity of the assembly is insufficient to conclusively rule out the absence of the remaining two genes (*Hox1* and *Hox5*) or to determine the genomic organization of the HOX gene cluster. However, a much more extensive annotation effort will be necessary not only for *D. vexillum* but also for tunicate genomes in general, in order to produce a more complete picture of the functional landscape.

The new assembly increased the number of detected ncRNA families to 4877 genomic loci corresponding to 271 families. From these, most of the detected *loci* were *housekeeping* ncRNAs (rRNAs, tRNAs, snRNAs, and snoRNAs) and those *loci* were found in a conserved cluster organization, as seen on tRNAs, rRNAs, and snRNAs. At the same time, a new set of regulatory ncRNAs (miRNAs, Cis-regulatory RNAs and lncRNAs) were detected. As expected, the conserved set of miRNAs were annotated: mir-124, mir-598, mir-7, let-7, mir-1, mir-133, mir-33, lin-4, mir-137, mir-153, mir-2, mir-31, mir-449, mir-183, mir-190, mir-210, mir-219, and mir-8. In comparison to previous miRNA tunicate surveys [20,86], we validated previous reports of tunicate-specific mir-1497 (RF00953), and also reported additional specific families, such as ciona-mir-92 (RF01117) and mir-281 (RF00967), by detecting their mature position and evaluating them along a secondary family specific structural multiple alignment. Further studies will allow us to continue to refine the complete miRNA complement in *D. vexillum* and reconstruct the evolutionary history of miRNAs in the tunicates. We were not able to identify homologs of other expected ncRNA families, as: *vault*, *U7* and *Y* RNA and *Telomerase* RNA.

The new assembly of the *D. vexillum* genome described here provides an integrated effort to contribute to the ongoing Tunicata genome projects and constitutes the first annotation dataset for a species in the Aplousobranchia. We hope that the new *D. vexillum* genome annotation presented here triggers more biological studies in a representative of a highly invasive species with a colonial life history.

## Figures and Tables

**Table 1:** Comparison of first draft [26] and the new draft assembly of the *D. vexillum* genome.

Assembly	Estimated Size (kb)	Number Contigs (c)/ Scaffolds (s)	L50	N50	GC Content	IUPAC	Putative Gene Number	Putative Protein Number
Draft [26]	542,259	882,106 (c)	152,090	918	0.366±0.063	0.000	N/A	N/A
This work	517,553	109,769 (s)	25,281	6539	0.362±0.024	0.0155	62,194	64,424

**Table 2:** Annotated ncRNAs families and *loci* (in parentheses) in the *D. vexillum* genome. *Homology* corresponds to previously reported numbers of ncRNAs by homology [26], *Mapped* corresponds to the number of ncRNAs that were mapped in the first genome draft [26]. *Final* corresponds to the current list of candidate ncRNAs. *NA:* Not available.

ncRNA Family	Homology	Mapped	Final
Cis-Reg	3 (333)	0	3 (333)
miRNAs	248 (2065)	17 (20)	235 (1582)
misc RNAs	1 (1)	1 (1)	2 (2)
lncRNAs	2 (8)	0	2 (8)
Ribozyme	3 (11)	0	3 (11)
rRNAs	4 (84)	0	4 (84)
snoRNAs	6 (9)	6 (9)	12 (18)
snRNAs	9 (87)	2 (34)	9 (115)
tRNAs	23 (2724)	NA	23 (2724)
• mt-tRNAs	0	21	21
• mt-rRNAs	0	2	2
Total	277 (5322)	26 (64)	271 (4877)

## Data Availability

The reported data can be accessed at http://tunicatadvexillum.bioinf.uni-leipzig.de/Home.html, accessed on 3 December 2021.

## Supplementary Materials

The Supplementary Materials are available online at https://www.mdpi.com/article/10.3390/life11121377/s1.

## Abbreviations

   The following abbreviations are used in this manuscript:

BUSCO	Benchmarking Universal Single-Copy Orthologs
CM	covariance model
Hh	*Hedgehog*
HMM	Hidden Markov Models
HOX	homeobox
lncRNA	long non-coding RNA
ML	Maximum Likelihood
miRNA	microRNA
misc RNAs	miscellaneous RNAs
ncRNA	non-coding RNA
RMST	Rhabdomyosarcoma 2-associated transcript
rRNA	ribosomal RNA
snRNA	small nuclear RNA
snoRNA	small nucleolar RNA
tRNA	transfer RNA
