# Supplementary material for: The Genome of the “Sea Vomit” Didemnum vexillum
Source: Life (Basel). 2021 Dec 10;11(12):1377. doi: 10.3390/life11121377 (PMC8704543; doi:10.3390/life11121377)
Supplement: Supplementary file 1 [file life-11-01377-s001.zip › Figures/alignProteins.pdf]

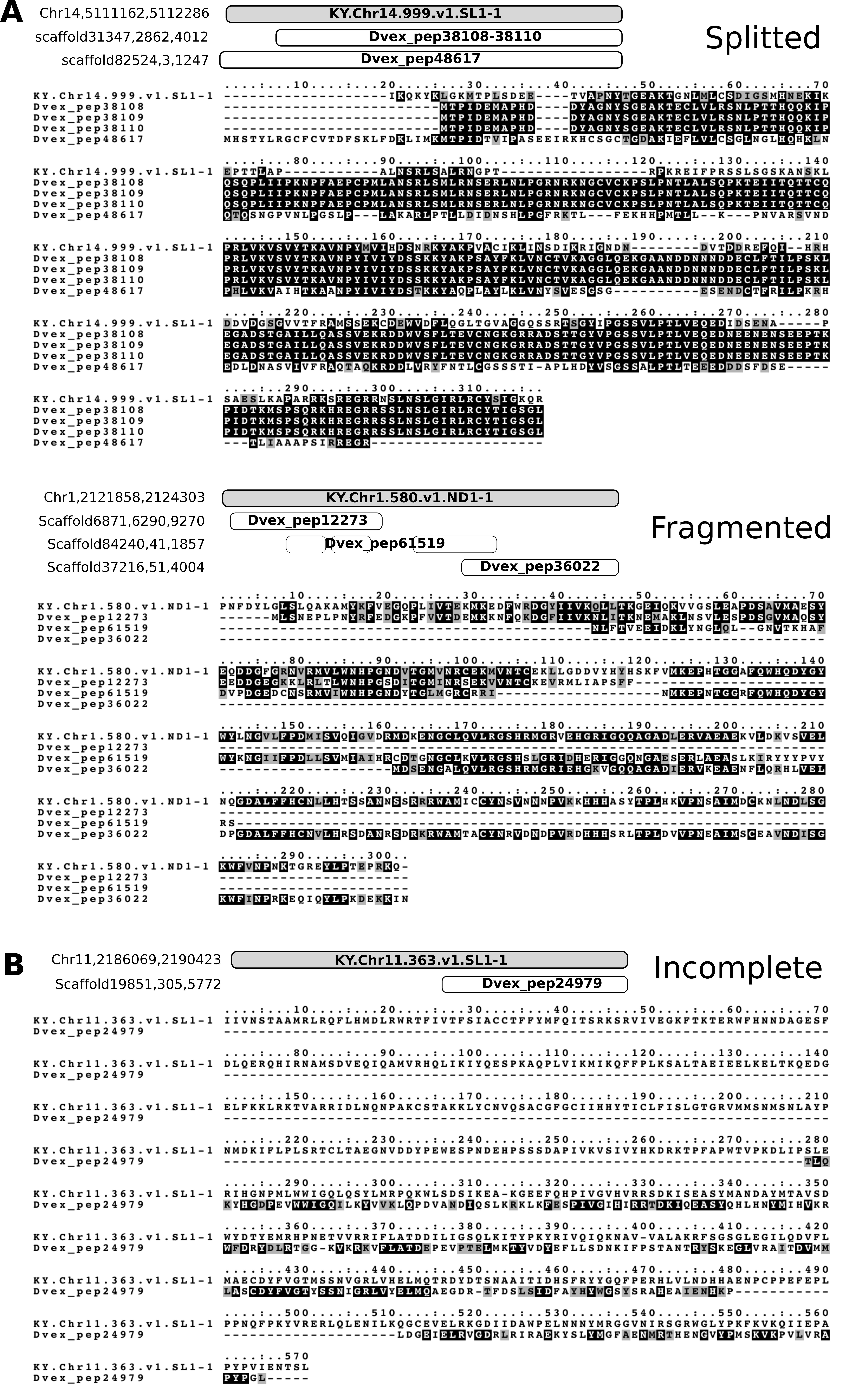
A
Chr14,5111162,5112286
KY.Chr14.999.v1.SL1-1
scaffold31347,2862,4012
Dvex_pep38108-38110
scaffold82524,3,1247
Dvex_pep48617
Splitted
KY.Chr14.999.v1.SL1-1
Dvex_pep38108
Dvex_pep38109
Dvex_pep38110
Dvex_pep48617
Chr1,2121858,2124303
KY.Chr1.580.v1.ND1-1
Scaffold6871,6290,9270
Dvex_pep12273
Scaffold84240,41,1857
Dvex_pep61519
Scaffold37216,51,4004
Dvex_pep36022
Fragmented
KY.Chr1.580.v1.ND1-1
Dvex_pep12273
Dvex_pep61519
Dvex_pep36022
B
Chr11,2186069,2190423
KY.Chr11.363.v1.SL1-1
Scaffold19851,305,5772
Dvex_pep24979
Incomplete
KY.Chr11.363.v1.SL1-1
Dvex_pep24979
