# Supplementary material for: The Genome of the “Sea Vomit” Didemnum vexillum
Source: Life (Basel). 2021 Dec 10;11(12):1377. doi: 10.3390/life11121377 (PMC8704543; doi:10.3390/life11121377)
Supplement: Supplementary file 1 [file life-11-01377-s001.zip › Figures/dive-enrichment-complete.pdf]

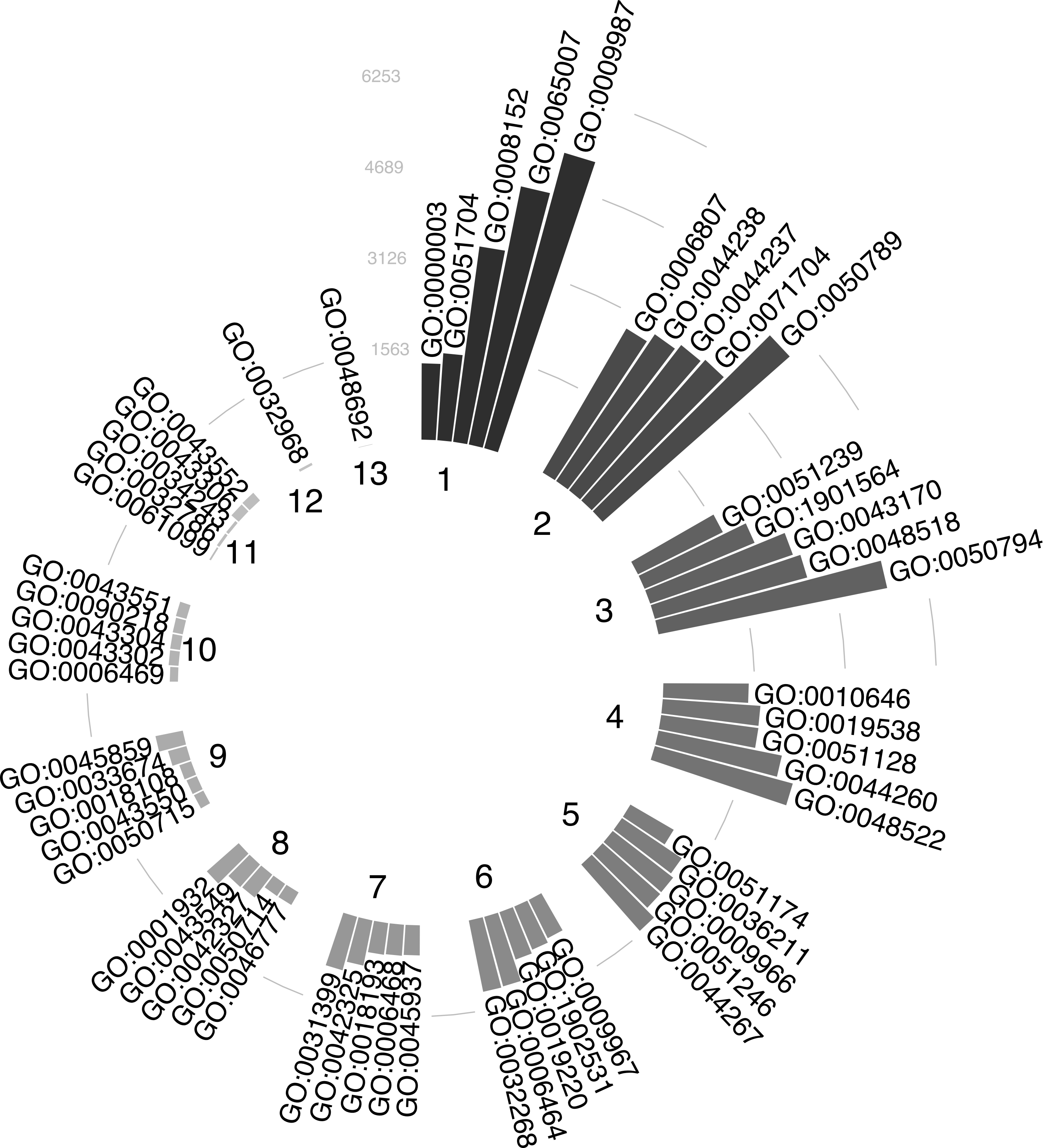

Depth	ID	Term	Count
1	GO:0065007	biological regulation	4520
1	GO:0009987	cellular process	5253
2	GO:0071704	organic substance metabolic process	3219
2	GO:0050789	regulation of biological process	4287
3	GO:0048518	positive regulation of biological process	2711
3	GO:0050794	regulation of cellular process	4007
4	GO:0044260	cellular macromolecule metabolic process	2161
4	GO:0048522	positive regulation of cellular process	2454
5	GO:0051246	regulation of protein metabolic process	1319
5	GO:0044267	cellular protein metabolic process	1519
6	GO:0006464	cellular protein modification process	1237
6	GO:0032268	regulation of cellular protein metabolic process	1249
7	GO:0042325	regulation of phosphorylation	790
7	GO:0031399	regulation of protein modification process	951
8	GO:0043549	regulation of kinase activity	514
8	GO:0001932	regulation of protein phosphorylation	730
9	GO:0033674	positive regulation of kinase activity	327
9	GO:0045859	regulation of protein kinase activity	478
10	GO:0090218	positive regulation of lipid kinase activity	176
10	GO:0043551	regulation of phosphatidylinositol 3−kinase activity	187
11	GO:0043306	positive regulation of mast cell degranulation	166
11	GO:0043552	positive regulation of phosphatidylinositol 3−kinase activity	175
12	GO:0032968	positive regulation of transcription elongation from RNA polymerase II promoter	43
13	GO:0048692	negative regulation of axon extension involved in regeneration	8
