# Supplementary material for: The Genome of the “Sea Vomit” Didemnum vexillum
Source: Life (Basel). 2021 Dec 10;11(12):1377. doi: 10.3390/life11121377 (PMC8704543; doi:10.3390/life11121377)
Supplement: Supplementary file 1 [file life-11-01377-s001.zip › Figures/DiveC-trna-resume.pdf]

Didemnum vexillum

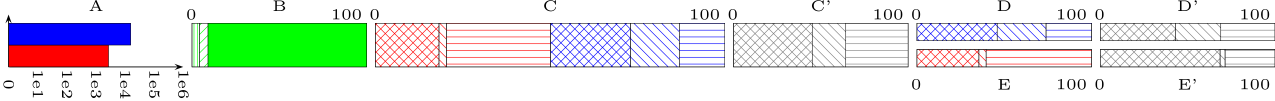

Legend:
A Distribution of tRNA genes (red) and tRNA pseudogenes (blue) on a logarthmic scale
B Fraction of tDNAs located in:
genomic clusters ||| homogeneous clusters // heterogeneous clusters (green) not located in clusters.
C Fraction of homogeneous pairs:
pairs tRNA genes XX →→, \\ →←, ≡ ←→ pairs tRNA pseudogenes XX →→, \\ →←, ≡ ←→
C' Fraction of Heterogeneous pairs: XX →→, \\ →←, ≡ ←→
D, **D'** analogous to **C**, **C'** separately for tRNA genes
E, **E'** analogous to **C**, **C'** separately for tRNA pseudogenes
