# Supplementary material for: The Genome of the “Sea Vomit” Didemnum vexillum
Source: Life (Basel). 2021 Dec 10;11(12):1377. doi: 10.3390/life11121377 (PMC8704543; doi:10.3390/life11121377)
Supplement: Supplementary file 1 [file life-11-01377-s001.zip › Figures/final_sizeGC_end.pdf]

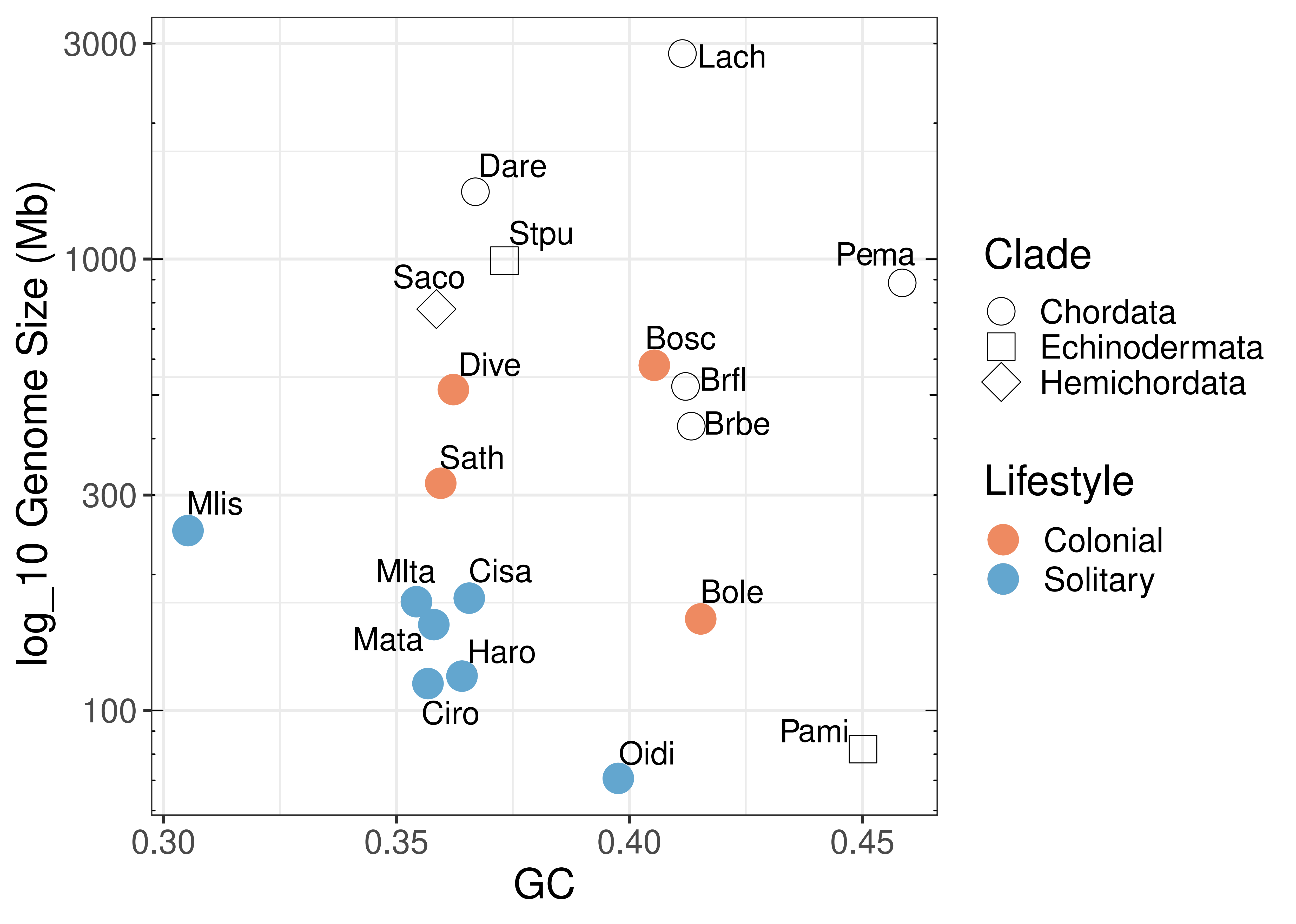

3000
1000
300
100
log_10 Genome Size (Mb)
0.30
0.35
0.40
0.45
GC
Lach
Dare
Stpu
Saco
Pema
Bosc
Dive
Brfl
Brbe
Sath
Mlis
Mlta
Cisa
Bole
Mata
Haro
Ciro
Pami
Oidi
Clade
Chordata
Echinodermata
Hemichordata
Lifestyle
Colonial
Solitary
