# Supplementary material for: The Genome of the “Sea Vomit” Didemnum vexillum
Source: Life (Basel). 2021 Dec 10;11(12):1377. doi: 10.3390/life11121377 (PMC8704543; doi:10.3390/life11121377)
Supplement: Supplementary file 1 [file life-11-01377-s001.zip › Figures/miRNAstructuralgroups.pdf]

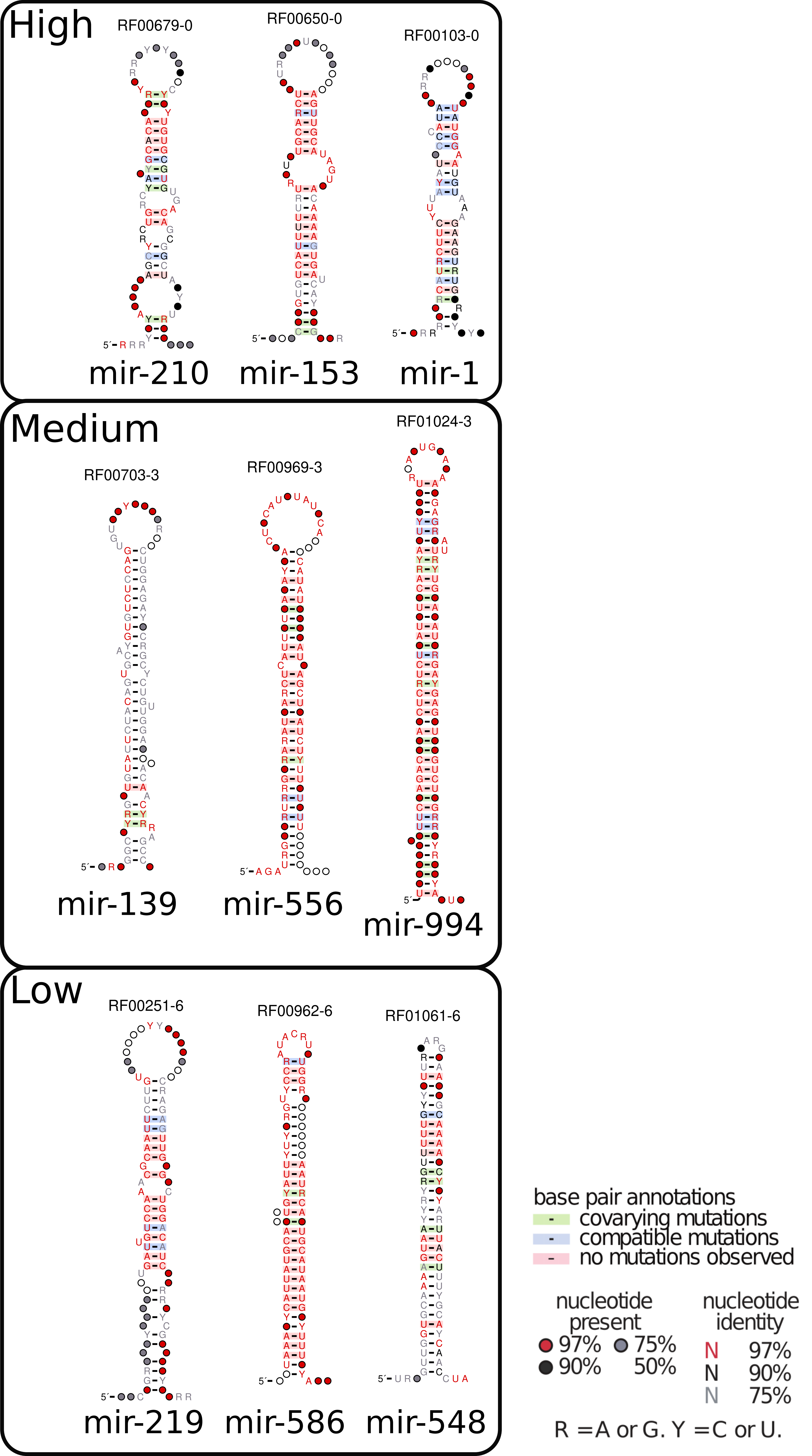
High
RF00679-0
RF00650-0
RF00103-0
mir-210
mir-153
mir-1
Medium
RF00703-3
RF00969-3
RF01024-3
mir-139
mir-556
mir-994
Low
RF00251-6
RF00962-6
RF01061-6
mir-219
mir-586
mir-548
base pair annotations
covarying mutations
compatible mutations
no mutations observed
nucleotide present
97%
75%
90%
50%
nucleotide identity
N 97%
N 90%
N 75%
R =A or G. Y =C or U.
