# Supplementary material for: The Genome of the “Sea Vomit” Didemnum vexillum
Source: Life (Basel). 2021 Dec 10;11(12):1377. doi: 10.3390/life11121377 (PMC8704543; doi:10.3390/life11121377)

Solitary tunicates specific GOs

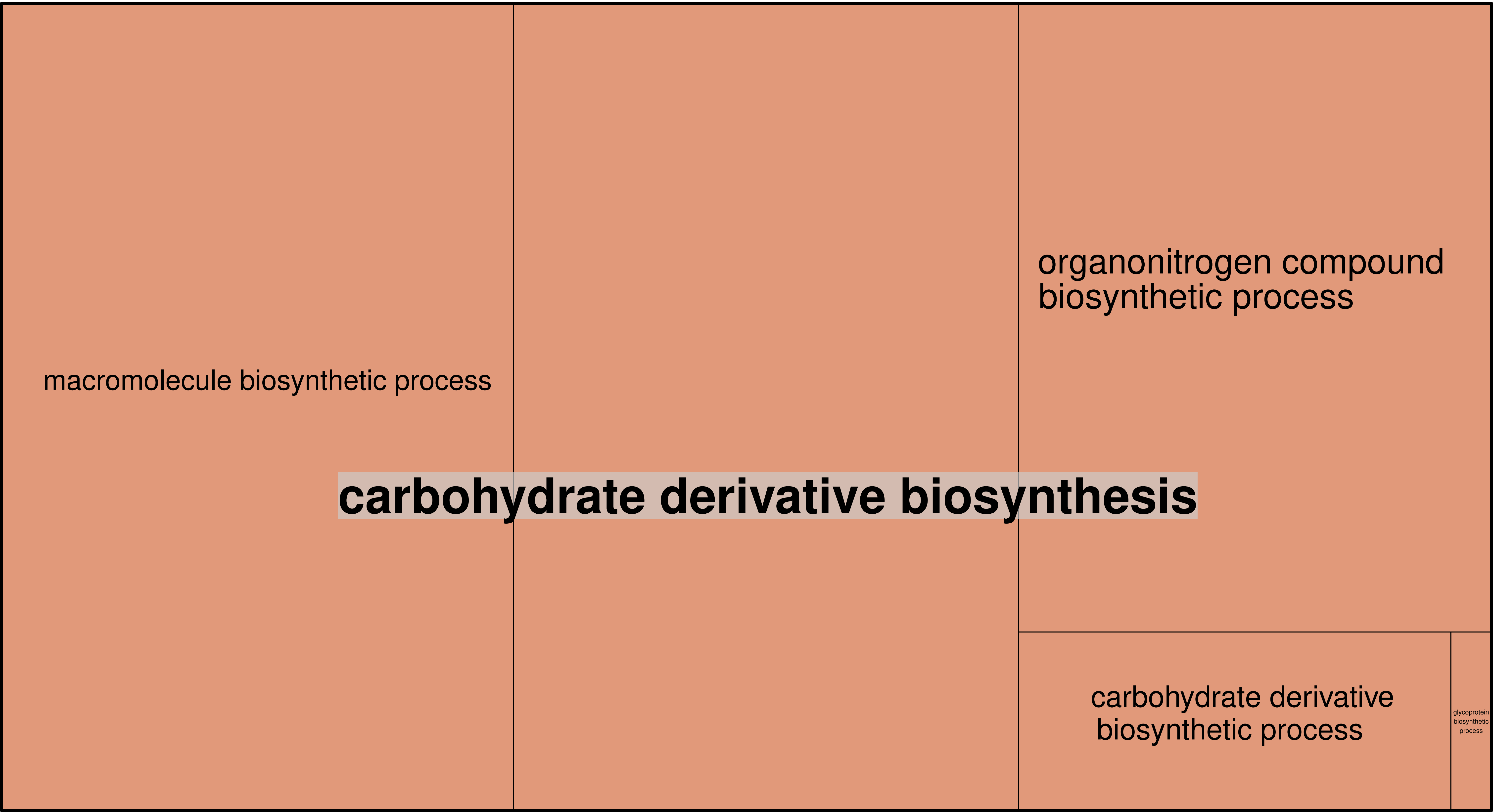

Supplement: Supplementary file 1 [file life-11-01377-s001.zip › Figures/revigo_treemap_solitary.pdf]
