# Supplementary material for: The Genome of the “Sea Vomit” Didemnum vexillum
Source: Life (Basel). 2021 Dec 10;11(12):1377. doi: 10.3390/life11121377 (PMC8704543; doi:10.3390/life11121377)
Supplement: Supplementary file 1 [file life-11-01377-s001.zip › Figures/rmst_distribution.pdf]

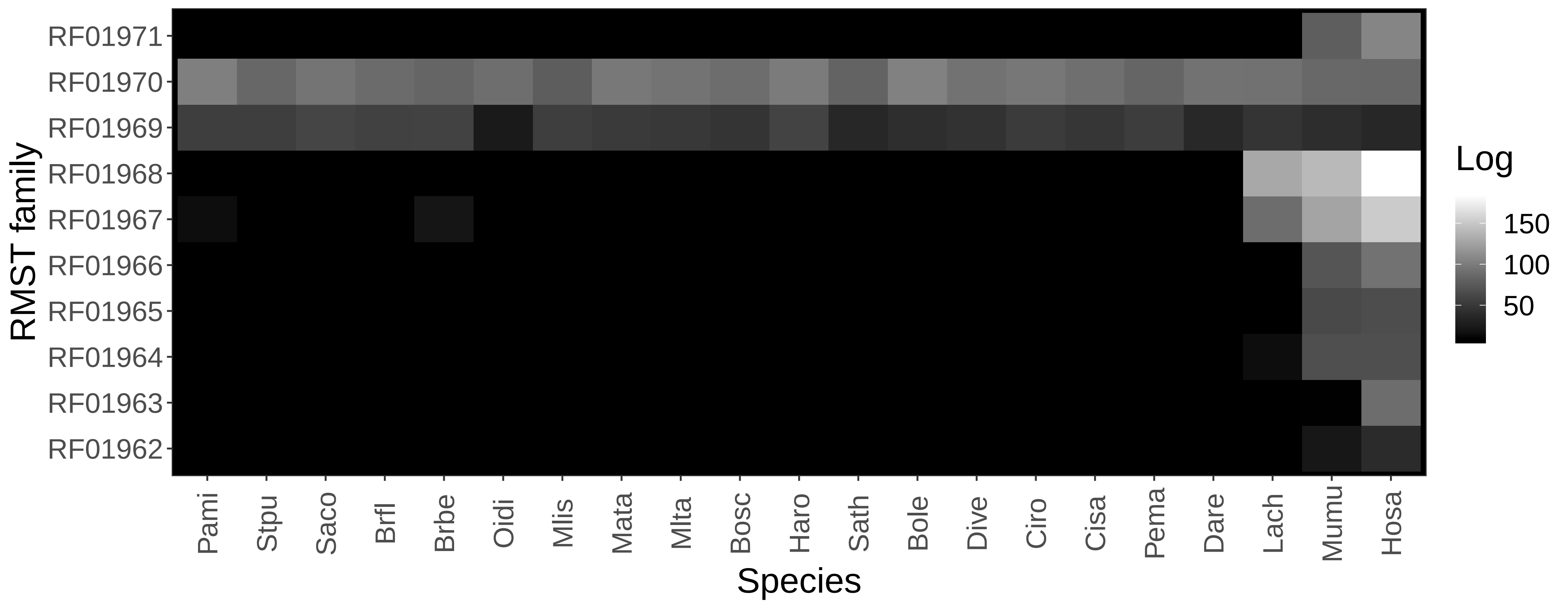
RF01971
RF01970
RF01969
RF01968
RF01967
RF01966
RF01965
RF01964
RF01963
RF01962
RMST family
Pami
Stpu
Saco
Brfl
Brbe
Oidi
Mlis
Mata
Mlta
Bosc
Haro
Sath
Bole
Dive
Ciro
Cisa
Pema
Dare
Lach
Mumu
Hosa
Species
Log
150
100
50
