# Supplementary material for: The Genome of the “Sea Vomit” Didemnum vexillum
Source: Life (Basel). 2021 Dec 10;11(12):1377. doi: 10.3390/life11121377 (PMC8704543; doi:10.3390/life11121377)
Supplement: Supplementary file 1 [file life-11-01377-s001.zip › supp.pdf]

Additional file #1: The genome of the "Sea Vomit" *Didemnum vexillum*

Ernesto Parra-Rincón, Cristian A. Velandia-Huerto, Adriaan Gittenberger,
Jörg Fallmann, Thomas Gatter, Federico D. Brown,
Peter F. Stadler and Clara I. Bermúdez-Santana

1 DNA extraction from *D. vexillum*

Table S 1: Summary of 29 DNA extractions on *D. vexillum*. Extraction dates: [J]: July 2015. [O]: October 2015. [D]: Second week of December, 2015. [F]: February 2016. [a]: `GiMaRIS` collection number AG4844.

Collection Date	Sample	Tissue Preservation	Extraction (N)	Result	Problem
June 10th, 2015	S1[a]	96% ethanol + 4.0°C in fridge.	E1[J] (1)	Little to no DNA (Nanodrop analyses).	-
	S2	−80.0°C freezer	E2[O] (1)	No DNA.	-
September 2015	S3	−20.0°C freezer	E3[O] (1)	19-25 μg sample, sent to Macrogen and Pacbio. E3=359.4 ng/μl.	Contained only 2 ng of DNA after quantity checks, indicating DNA-degradation (54.8 ng/μl).
Third week of November, 2015	S4	Ethanol + −20.0°C freezer	E4,E5[D] (2)	100 μl samples, sent to Macrogen. E4 = 396.2 ng/μl, E5= 222.6 ng/μl	Too fragmented
			**E6,E7[D] (2)**	100 μl samples, sent to PacBio. E6 = 694.4 ng/μl and E7 = 246.3 ng/μl	Usable DNA, partial DNA-degradation. E6 = 386 ng/μl and E7 = 192 ng/μl.
			E8-E12[D] (4)	DNA present (Nanodrop)	Not selected for further analyses as E4 to E7 gave better Nanodrop quantity/quality values.
			E13-E29[P] (16)	Seven extractions sent to Macrogen and PacBio. DNA present in all samples. The best quality and quantity on 7 out 16 extractions, which were used for further analyses.	Pooled longer fragments have shown smears. Not suitable to further analyses.

2 Data preprocessing and pre-assembling

Size distribution of sequenced subreads is shown in Figure S1.

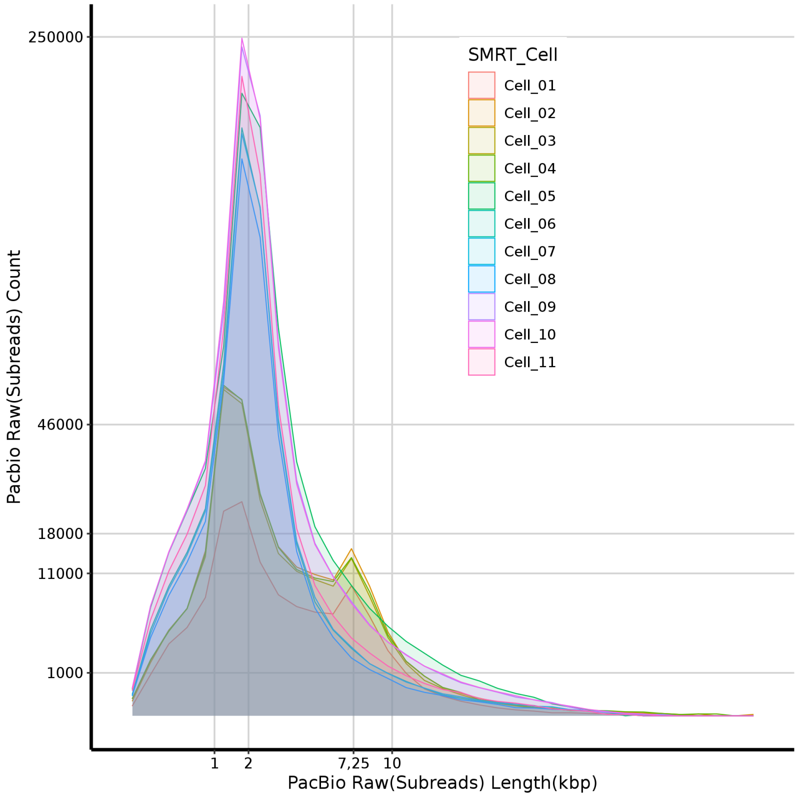

Figure S 1: Size distribution of sequenced PacBio SubReads from eleven SMRT PacBio cells. The output of sequencing for each cell is plotted: PacBio raw (SubReads) counts in X axis and in Y axis the length in kilobase pair (kbp) for the PacBio raw (SubReads).

Table S 2: Data used to be run on the new hybrid assembly.

	**Correction**	**Software**	**Reads**	**N50**	**Size**
1	Hybrid	Proovread	776,295	3.94kbp	2.7Gp
2	Hybrid	Proovread	288,198	1.7kbp	391Mbp
3	Pre-assembled reads	SMRT pipe	823,758	1.8Kbp	1.4Gpb
4	CCS	SMRT pipe	220,514	2.1Kbp	450 Mbp

3 Alternative Assembly Strategies

The assembly quality obtained with the pipeline described in main text (Section 2.2) is below expectations given the available read coverage. In order to ensure that this is not an error, we chose methods based on their reported competitiveness and contrasting assembly strategies, including short read only assembly (ABySS [?]), hybrid assemblers (DBG2OLC [?], Wengan [?]), our in-house development LazyB [?], and a long read only assembly method (wtdbg2 [?]). See Section S12 for details.

Initial approaches to assemble the *Didemnum vexillum* genome

We applied five standard methods to the assembly of the *D. vexillum* genome. The results of these attempts are summarized in Table S3 in terms of commonly used parameters. None of these tools was able to capture a significant portion of the genome, covering 20% or less of our final assembly at a very low rate of disjoint contigs, see Table S3.

Table S 3: Assemblies obtained with alternative strategies. Tools were used as recommended by the respective developers. Genome statistics are given for independent assemblies (Genome Size, Number of Contigs), and in comparison to the assembly described in the previous section (computed with QUAST [?]). We report the number of contigs not matching to the reference (Disjunct Contigs) and the fraction of the reference that is covered by the alternative assembly (Match Fraction). The covered fraction is likely underestimated due to the high level of alignment noise breaking up true matches. Completeness was evaluated by Benchmarking Universal Single-Copy Orthologs (`BUSCO`) using the metazoan lineage, as the percentage of (partially) assembled genes.

Method	Genome Size (kb)	# Contigs	# Disjunct Contigs	Match Fraction (%)	BUSCO %
ABySS	2,289	2,772	76	0.413	0.2
DBG2OLC	135,699	35,393	687	20.851	17.4
LazyB	76,276	30,369	1732	8.404	18.4
Wengan	176	22	1	0.031	0
wtdbg2	4,713	736	19	0.768	0.4

Manual inspection of the genome shows a very high level of genetic variation, which is most likely the root cause of the limited quality of the assemblies, see Figure S2. Indeed, only DBG2OLC and LazyB, i.e., the tools least susceptible to genetic variance within samples, were able to produce even a partially acceptable assembly. Utilizing the Celera Assembler and varied pre-processing techniques allows us to map more dissimilar region and therefore also to assemble more of the genome. We subjected this intuition to more systematic testing, see Section 4 below.

4 Analysis of Genetic Variance

To analyze k-mer statistics, we used jellyfish [?] at k =21 to count all k-mers in the processed Illumina reads and analyzed the distribution with GenomeScope 2.0 [?]. Merqury [?] was used to match the same set of k-mers against our reference assembly.

To directly estimate diversity we randomly selected 100 genes from the catalog of single-copy orthologs identified by `BUSCO` without duplication in our assembly. In this manner we obtained a set of regions without duplication and evolutionary pressures to remain stable. Short reads were then aligned to all genes with BWA at conservative scoring. We used freebayes [?] with options *--pooled-continuous* and *--pooled-discrete* as recommended. Results were filtered for very high quality of 30 and above.

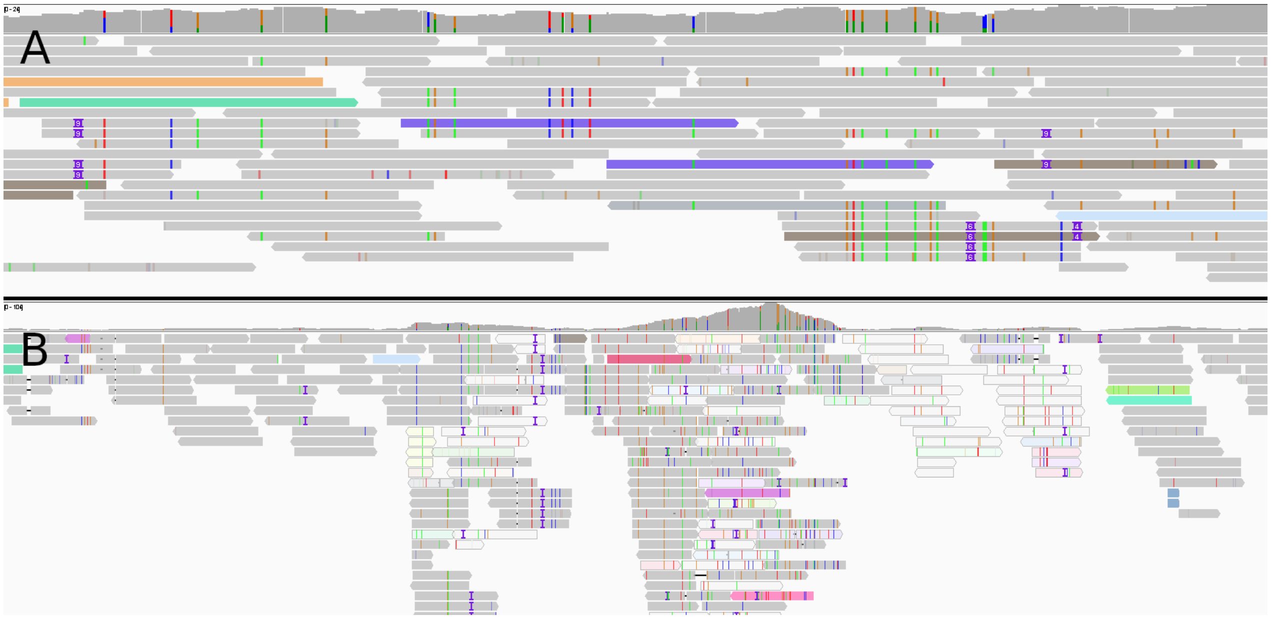

Figure S 2: Short reads mapped to the *D. vexillum* genome assembly at the locus of typical single copy orthologs predicted by `BUSCO` [?] A) EOG091G0VTN ribosomal protein L14 and B) ribosomal protein L37a. The regions show extensive and (partially) systematic genetic variation. The image was generated with IGV [?]. Colored reads indicate unexpected insert sizes or mate-pairs on other scaffolds. Base variants are marked as colored bars. On top DNA-seq coverage is shown for both `BUSCO` genes.

To isolate the cause for the poor performance of all standard assembly pipelines we further investigated the available data. Statistics based on k-mers are routinely utilized to evaluate the genome assemblies in a reference-free setting. `GenomeScope` [?] was developed for pre-assembly quality control by fitting a genome model to the k-mer profile and estimating several key statistics.

Although `GenomeScope` was able to fit a model, the k-mer spectra do not show the distinct peaks that are expected for a diploid organism, even in the transformed model to enhance observable peaks (Figure S3). Instead, the k-mer profile exhibits a strong peak of very low abundant k-mers that is modeled as noise. Since it is highly unlikely that this profile is caused is by extreme error levels in the Illumina reads, in particular given the otherwise encouraging quality control parameters, the signal is presumably caused by a very high level of genetic diversity.

`GenomeScope` estimates a genome size of 219 Mbp, i.e., more than 2 times smaller than our assembly. Although we cannot unambiguously eliminate the possibility of duplications in our assembly, highly variable regions are likely discarded and therefore missing in the `GenomeScope` model. The strong divergence from an expected distribution also renders this estimation highly unreliable. To corroborate this interpretation we ran `Merqury` [?] to match the same set of k-mers against our reference assembly. We observe that the "genomic peak" again is overlaid by unused k-mers deriving from either noise or genetic diversity (Figure S4). According to this assessment, our assembly exhibits a remarkably low level of duplication. This unfortunately does not guarantee actual correctness. As k-mers lack in precision at highly divers sites, highly divergent haplotypes may simply not be recognized as such and thus also not marked as duplication.

Although the sequencing protocol was not well suited to compute accurate polymorphism and divergence statistics, we can nevertheless obtain plausible approximations by considering reads mapping to single-copy genes as identified by `BUSCO`. Variant sites were detected on average every 363 basepairs, predicting alternatives for every 246th base. Up to 11 variants were called for a single site, averaging at 2. Variants made up on average 41.5% of reads in each site.

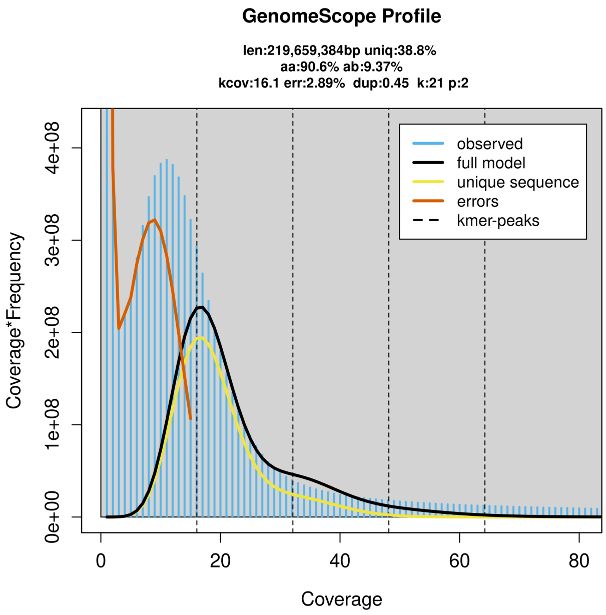

Figure S 3: K-mer spectra and fitted models for Illumina short reads ($k = 21$). Designated erroneous k-mers largely overlap the predicted genome. The distance between unique sequence and full model, at a "read error rate" of 3%, which cannot realistically be explained by technical error rate of the Illumina reads, but rather signifies a high diversity in the predicted genome. X-axis: k-mer frequency, Y axis: k-mer multiplicity (i.e. coverage).

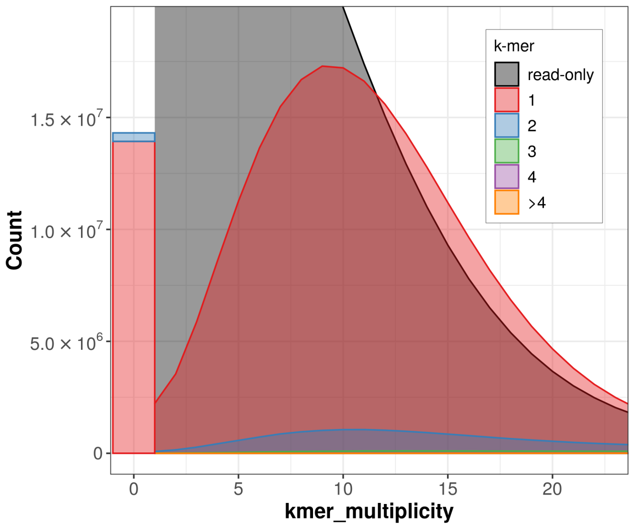

Figure S 4: Copy number spectrum plot generated by Merqury for k-mers ($k = 21$ as recommended by the developers) of Illumina short reads plotted as stacked histograms coloured by the copy numbers in our newly assembled genome. Only a low level of duplication is detected. Noisy k-mer overlay genome peak. X-axis: k-mer count, Y axis: k-mer multiplicity (i.e. coverage).

5 Post-processing genome polishing

Detection of sources of contamination on *D. vexillum* genome

Following the described methodology on main text, 17439 query genomes were obtained from NCBI RefSeq[1]. Homologous high scoring pairs were searched using `blastn`, as follows:

```
blastn -db <DB> -query <QUERY_SEQ> -num_threads 8 -evalue 1e-10 -word_size 30 -gapopen 1
    -gapextend 2 -penalty -1 -reward 1 -dust yes -outfmt 6 -out <OUT>
```

The best hits were retrieved if they reported an identity $\geq$ 80% and did not present gaps in the alignment. With this strategy was detected a number of 526 species that reported high scoring pairs and the crossing with the current RefSeq annotation found only 3 contamination sources: *Acinetobacter baumannii* (NZ_CP020583.1), *Weissella cibaria* (NZ_CP013938.1) and a *Adelie penguin polyomavirus isolate AdPyV_Crozier_2012* (NC_026141.2).

By other way, specific covariance models from bacteria (807) obtained from RFAM v.12 were searched on *D. vexillum* genome with `cmsearch` as described on Section S12. Only one locus from one ncRNA family were found, related with the *cis*- regulatory ALIL pseudoknot (RF01497).

At the end, following the described strategies 4 scaffolds were removed from the original genome assembly, it means $\sim$ 18.65 kb. Those sequences were removed from the final available genome.

Detection of repeated contigs on *D. vexillum* genome

Along all the assembled scaffolds on *D. vexillum* that reported at least one ncRNA *loci*, an evaluation of repeated-blocks was performed using `lastz` with *self-alignment* parameters (as referred on Section S12), to detect those contigs that were constituted with repeated blocks along all the reported sequences. A complete analysis of the output files from `lastz` allowed to survey the features from the detected alignment blocks: length and number of repetitions along the same contig. Based on the distribution of both variables, threshold values were defined (as detailed on Figure S5). In this case, a scaffold is classified as repeated if reported in at least one of the detected repeated-blocks length $\geq$ 100 nt or number of repetitions $\geq$ 10, it is reflected on Figure S5C, *Max* label. At the end, 540 contigs were classified as repetitive contigs and discarded from the final assembly.

6 Re-annotation of the *Didemnum vexillum* genome

Features of annotated genes

The number of the genes with the median of size in kb ($\bar{L}$) and their number of transcripts are described on Table S4. Additionally, more detailed view of the complete data could be addressed on the Figure S6, which the complete range of sizes discriminated by number of transcripts are depicted. In this case, most of the *D. vexillum* genes reported only one transcript and their sizes reported wider range in comparison to those genes that reported $>$ 1 transcripts.

In more detail, taking into account the length of the transcription products, their reported median size is $\sim$ 0.375 kb along a positive skewed, long-tailed distribution, where 2 transcripts have been reported as outliers with $\geq$ 10 kb: *Divexi.CG.Dive2019.scaffold1420-size16704.g13695-mRNA-1.t* ($\sim$ 12.3 kb) and *Divexi.CG.Dive2019.scaffoldUncertain.g539-mRNA-1.t* ($\sim$ 12.11) kb. For the first one, the corresponding protein is `Dex_pep14095`, that accounts for a homologous candidate on the solitary tunicate *C. robusta*:

[1]ftp://ftp.ncbi.nlm.nih.gov/genomes/refseq/assembly_summary_refseq.txt

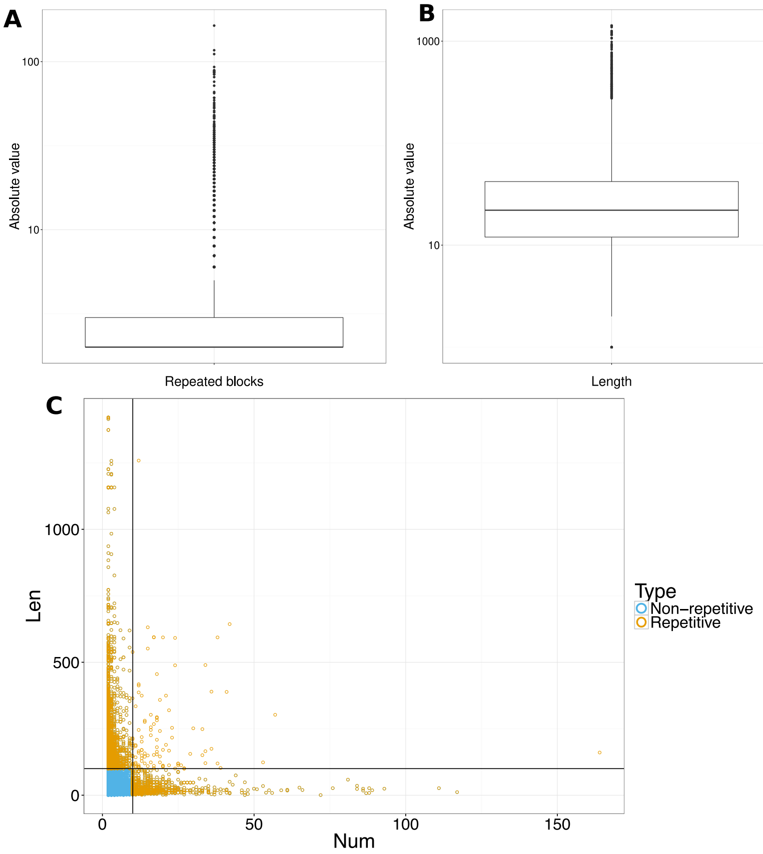

Figure S 5: Features from repeated-blocks on *D. vexillum* contigs that reported ncRNA candidates. **A.** Distribution of repeated blocks along all the contigs. **B.** Distribution of lengths of all repeated-blocks. **C.** Number of repeated-blocks (*Num*) against their lengths, defined threshold values are shown as intersecting lines on axis, based on this classification, the *Repetitive* blocks help to discriminate between repeated and non-repeated contigs (see main text).

`A0A3Q0K5T4_CIOIN` which has annotations of VWFD, C8 and TIL protein domains on the UniProtKB database (version 2019_08) and has the status as an uncharacterised protein. For the second one, the `Dvex_pep554` protein, another homologous protein was found in *C. robusta* which corresponds to the `A0A1W5BP75_CIOIN`, but its annotation corresponds to an uncharacterised protein, too. Last results were achieved using `jackhmmer` against `Uniprotkb (v.2019_08)`.

Analysis of protein products from: `Divexi.CG.Dive2019.scaffold15233-size8129.g20706`

As indicated on the main text, gene `Divexi.CG.Dive2019.scaffold15233-size8129.g20706` reported the highest number of transcripts (10) along the *D. vexillum* annotation. Here, 3 longer protein products were analyzed with `phmmer` against the `UniProtKB` database (v.2019_08) using `HmmerWeb` v2.40, as indicated in Table S5.

Detection and annotation of Hox genes

Based on the described methodology in the main text to detect the homologous candidates from the Hox genes. The final list of candidates annotated on the current *D. vexillum* assembly is described on Table S6.

Table S 4: Transcript number summary from *D. vexillum* annotated genes.

**Number of transcripts**	$\bar{L}$ **(kb)**	**Number of genes**
1	0.9730	60668
2	1.8020	1131
3	0.6730	253
4	0.4780	53
5	0.5155	40
6	0.5445	30
7	0.5450	13
8	0.4390	3
9	2.5540	2
10	7.0670	1

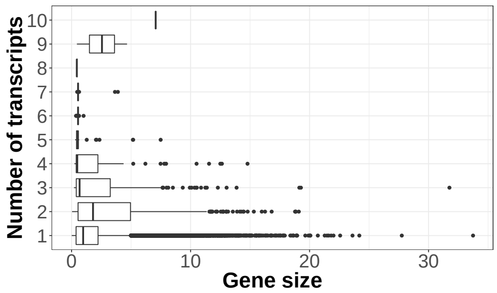

Figure S 6: Frequency of the number of transcripts along genes from *D. vexillum.*

Table S 5: Homologs of the biggest protein products of `Divexi.CG.Dive2019.scaffold15233-size8129.g20706` which reported the largest number of transcripts on the *D. vexillum* annotation. Data obtained by `phmmer` against UniprotKB database.

**Protein product name**	$\bar{L}$ **(kb)**	**Annotation**	**Found Homolog**	**E-value**
`Dvex_pep21349`	259	Dynein heavy chain	A0A210Q030_MIZYE	$2.8e^{-18}$
`Dvex_pep21351`	217			$6.9e^{-15}$
`Dvex_pep21350`	217			$5.3e^{-13}$

Mapping previous ncRNA annotation on new assembly

Previous ncRNA annotation was retrieved [?] in fasta format. All the contigs which reported a ncRNA annotation have been obtained from the reported draft assembly of the *D. vexillum* genome[2]. The resulting was mapped onto the new genome with `lastz`:

```
lastz_32 <NEW_GENOME>[multiple] <OLD_GENOME> --rdotplot=<OUT_DOT-PLOT_FILE>
--ambiguous=iupac --chain C=0 E=150 H=0 K=4500 L=3000 M=254 O=600
Q=human_chimp.v2.q T=2 Y=15000 --format=maf+ > <OUTPUT_FILE>
```

Alignment files were retrieved in `maf` format and were parsed with `Bio::AlignIO Bioperl` library. The criteria to obtain the best genome coordinates was chosen based on the relation between the length of the mapped region into the new genome (m) and the original size of the query contig in the old genome (s). The relation was defined as $R = \frac{m}{s}$, in this case, the best mapping candidates are those which reported $R = 1$, but to retrieve the maximum number of mapping between the two genome versions, $R \geq 0.90$ was also considered. From 247 contigs, was possible to map 213 in the raw results after the mapping stage with *lastz*, which generated 153892 relations. After considering the R relation, those results were parsed, resulting in: 1.09% ($R = 1$), 1.37% ($0.95 \leq R < 1$), 0.35% ($0.90 \leq R < 0.95$) and 0.27% ($0.85 \leq R < 0.90$), the remaining percentage of candidates (96.15%) reported $R < 0.85$, which in this strategy were considered as low mapping score. In the other hand, 111 contigs reported at least one high mapping score ($R \geq 0.85$).

At the same time, previously ncRNAs were obtained and mapped against the new *D. vexillum* assembly with `blastn`, as follows:

[2] `http://tunicata.bioinf.uni-leipzig.de/Download.html`

Table S 6: Final results from annotation of candidates of Hox gene family in the *D. vexillum* genome. **A**: Anterior, **C**: Central, **AP**: Ancestral Posterior. NA: not available. Species labels: **Brla**: *Branchiostoma lanceolatum*, **Ciin**: *C. intestinalis*, **Haro**: *H. roretzi*, **Hefr**: *Heterodontus francisci*, **Lame**: *Latimeria menadoensis*, **Bosc**: *B. schlosseri*, **Ciro**: *C. robusta*, **Cisa**: *C. savignyi*, **Bole**:*B. leachii*, **Oidi**: *O. dioica.*

Class	Hox Gene	Manual Curation	Homology Support	Alignment Support
A	Hox2	scaffold16549-size8805, 5363-5551,-	Dvex_pep49845,2876-6340,-, Brla, Ciin, Haro, Hefr, Lame	4226-4827, +, Bole, Bosc, Ciro, Cisa
		scaffold2010-size15357, 9838-9972, -	NA	9755-10025, +, Bole, Cisa, Haro
A	Hox3	scaffold11368-size9116, 1079-1225, -	NA 1072-1253, +, Bosc, Ciro, Cisa	
		scaffold57440-size3324, 880-1005, -	NA	877-1131, +, Ciro, Cisa, Haro
C	Hox4	scaffold72048-size2554, 2108-2254, +	NA	1980-2259, +, Bole, Bosc, Ciro, Haro
		scaffold12766-size8723, 1763-1909, -	NA	1267-2089, +, Ciro, Cisa
C	Hox6/7-like	scaffold34036-size5256, 585-779, -	NA	633-807, +, Haro
AP	Hox10	scaffold93625-size1680, 1125-1223, -	(Dvex_pep30911, Dvex_pep30912), 556-1226, -, Ciro, Cisa[a]	638-1292, +, Ciro,Cisa
AP	Hox12	scaffold4141-size12757, 8233-8496, +	NA	8146-8486, +, Bole, Ciro, Cisa, Haro
AP	Hox11/12/13.a	scaffold101308-size1277, 4-243, +	NA	10-389, +, Bole, Brfl, Ciro, Cisa, Oidi

[a]Not found by standard homology searches. Reported region has shown a high homology with a corresponding harbouring-Hox gene region in *Ciona* sp. Reported gene (Divexi.CG.Dive2019.scaffold93625-size1680.g29940) in *D. vexillum* was annotated on that region.

```
blastall -p blastn -d <DB> -i <QUERY> -F F -e 10e-5 -m 8 -o <OUT>
```

If one contig reported more than one candidate in the new genome, we chose the one with the highest `bitscore`. After mapping all the candidates with `blastn`, the true locations were obtained after applying the following filters:

- Identity have to be $\geq 85\%$.
- E-value $\leq 10^{-10}$.
- Relation of sizes between the homology region of the query (r_h) and their calculated size (r_s) have to be $\frac{r_h}{r_s} \geq 0.9$

An additional confirmation step was performed using the covariance models from `RFAM`v.14.1 onto the retrieved fasta sequences, using `infernal` package:

```
cmsearch -g -Z <NT number (Mb)> --toponly --tblout <OUT_TABULAR> -o <OUT_FILE> <FASTA> <
    CM>
```

After getting the merged coordinates, some candidates reported more than one position on the genome or even, the same positions shared with another candidate (s). In this case, the final reported mapped candidates were those that mapped 1 : 1 to the new assembly and does not share the same positions with an overlapping candidate. In this way, 77 loci were retrieved and had reported additional support from genome alignments, 36 have been identified on the new genome in another location that is different to the correspondent new region of the old contig. At the end it was possible to map 105 previously annotated candidates by this strategy which were included in the final set of candidates with the tag `MAPPED`, from those candidates, 8 reported an additional mapping position which were also included in the final results, due those candidates in the new assembly reported high homology scores, description of those families are in Table S7.

Table S 7: Annotated *loci* in the draft version of *D. vexillum* that reported more than one mapping position on the new alignment. Old coordinates are reported as: Name, Chromosome, Strand, Start, End. New coordinates are reported as: Chromosome, Start-End, Strand, (Bitscore, E-Value)

**Candidate**	**Supported by Alignment**	**Other position**
mir-276.dvex159218.+.706.786	scaffold9268-size9828, 4294-4374, Reverse, (38.8, 2.5e-10)	scaffold7042-size10846, 1855-1935, Forward, (34.0, 3e-09)
SNORD18.dvex152227.+.1372.1432	scaffold225-size23884, 4646-4706, Reverse, (26.8, 9.4e-07)	scaffold76090-size2378, 1649-1710, Reverse, (18.7, 2.8e-05)
U4atac.dvex622135.-.311.415	scaffold23895-size6623, 2192-2306, Forward, (37.5, 2.5e-08)	scaffold78418-size2279, 1339-1453, Forward, (59.1, 8.7e-12)
U6.dvex134697.+.19.112	scaffold66798-size2802, 2097-2193, Reverse, (40.5, 3.8e-09)	scaffold68538-size2716, 1005-1098, Forward, (41.1, 3e-09)
U6.dvex152032.+.83.175	scaffold30047-size5748, 717-809, Forward, (35.2, 3.2e-08)	scaffold97836-size1478, 229-325, Forward, (29.7, 3e-07)
U6.dvex435452.+.1786.1884	scaffold925-size18266, 4693-4791, Forward, (43.2, 1.3e-09)	scaffold17919-size7584, 1862-1964, Forward, (33.8, 5.8e-08)
U6.dvex595726.-.285.383	scaffold7916-size11956, 1129-1227, Forward, (26.8, 9.6e-07)	scaffold85789-size1993, 494-591, Forward, (31.7, 1.4e-07)
U6.dvex619958.+.139.235	scaffold28367-size5973, 674-770, Reverse, (36.4, 2e-08)	scaffold60312-size3152, 619-715, Forward, (36.4, 2e-08)

Searching missing rRNA and snRNA families

The housekeeping ncRNAs that could not be found on *D. vexillum* were searched on another tunicate genomes. Covariance models from small nuclear RNAs: U7 RNA (RF00066), vault RNA (RF00006) and Y RNA (RF00019) were applied on the reported genomes from *B. leachii*, *B. schlosseri*, *C. robusta*, *C. savignyi*, *H. roretzi*, *M. oculata*, *M. occulta*, *M. occidentalis*, *O. dioica* and *S. thompsoni* and with `cmsearch` as described on Command Line Methods (Section S12), the final results are described on Table S8, where positive candidates were found for *vault* snRNA along 9 species, but not in *O. dioica* and *D. vexillum.*

Table S 8: Presence/absence of housekeeping snRNA candidates on tunicates genomes. Reported tags are used to report the obtained values after evaluation by `cmsearch`: **B**: bitscore, **E**: E-value and **N**:Number of true candidates, if more than one candidate was reported the average of the bitscore and E-value are shown.

**Species**	**snRNAs**			
	**U7**	**vault**	**Y**	**Telomerase**
B. leachii	NA	B:57.6, E:3^{-10}, N:3	NA	NA
B. schlosseri	NA	B:46.9, E:9.8^{-7}, N:1	NA	NA
C. robusta	NA	B:48.6, E:7^{-8}, N:3	NA	NA
C. savignyi	NA	B:47.1, E:3.49^{-8}, N:4	NA	NA
H. roretzi	NA	B:57.85, E:1.65^{-10}, N:2	NA	NA
M. oculata	NA	B:54.9, E:1.75^{-9}, N:4	NA	NA
M. occulta	NA	B:59.8, E:8.30^{-11}, N:3	NA	NA
M. occidentalis	NA	B:59.8, E:3.05^{-8}, N:4	NA	NA
O. dioica	NA	NA	NA	NA
S. thompsoni	NA	B:44.7, E:3^{-8}, N:7	NA	NA
D. vexillum	NA	NA	NA	NA

tRNA supporting data

Comparison of tRNA predictions are described on Figure S7, which OLD and NEW labels corresponds to the reported draft assembly [**?**] and the new assembled version of *D. vexillum* genome, respectively. As a complement, as described in Figure S8, the distribution of tRNA genes has been updated and analyzed for the new assembly. As noted earlier and in the main text, there are a higher number of tRNA pseudogenes in comparison to tRNA genes. In general, most of the *D. vexillum* tRNAs are not taking part of a cluster, (based on the definition from [**?**]). The specific position are described as *pair* configuration, which in *D. vexillum* the most common is tail-tail along the tRNA genes for homogeneous clusters, while for heterogeneous ones, is tail-head.

Identification of mature position on RFAM families

Public `MySQL` Database from `RFAM` v.14.1 was used to obtain both, accession numbers and annotations of miRNA sequences. `RNAcentral` v.13 [**?**] was accessed to retrieve the stable identifiers between annotated sequences from `RFAM` and `miRBase` v.22. Based on these identifiers, the designated *seed* sequences by `RFAM` were classified as: *one to one* if they have available annotated *mature* sequences on `miRBase`. Those sequences that did not have any *mature* annotation were assigned as *one to many* group. For the first group, a validation of the reported mature positions were performed by `MIRfix` [**?**]. Based on those corrected sequences and positions, a new iteration of mature validation were performed including the second group of sequences, which did not report *mature* annotation. From those CMs that all

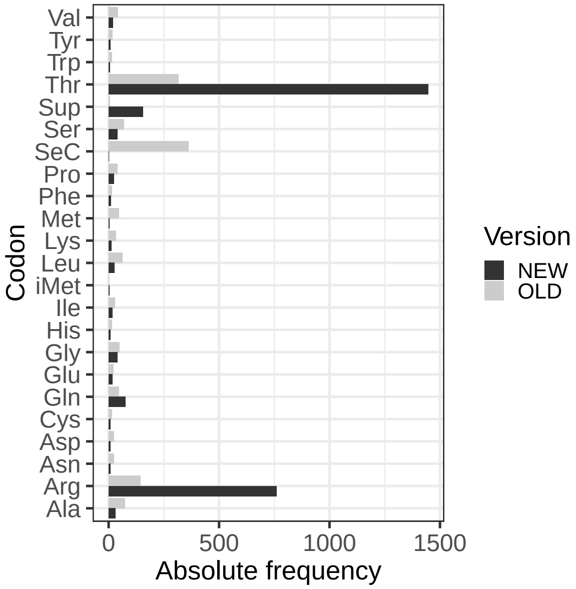

Figure S 7: Update of tRNA genes annotation on the new assembly from *D. vexillum* genome. Labels are: **OLD** for the reported assembly in [?] and **NEW** for this study.

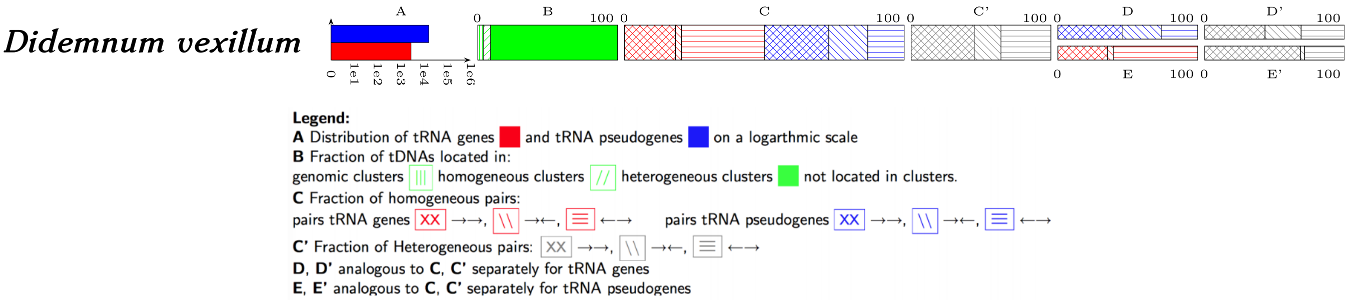

Figure S 8: tRNA clusters on *D. vexillum*

sequences were classified in the *one to many* group, the missing of specific *mature* annotations has been solved inferring the *mature* position based on the reported family-specific `stockholm` alignment, by the identification of conserved correspondent blocks along the alignment, which along the miRNA structure model would correspond to the *stem* region. Based on the previous results, the default `stockholm` alignments were corrected based on the position of the predicted/annotated *mature* sequences. Then, each previously detected miRNA from *D. vexillum* was corrected again with `MIRfix`, based on its previously inferred RFAM family. In cases where exists *loci* from the same family, each sequence was compared independently against the corrected set of *seed* sequences from RFAM. As a result, those miRNAs from *D. vexillum* which reported mature regions inside their predicted precursor and fit into the structural alignment, were considered as true candidates. The general methodology is explained on Figure S9.

From the 2084 miRNA *loci* detected by the described homology strategy, 1583 reported mature sequences harbored on their detected hairpin. Additionally, by including these sequences in their corresponding `RFAM` multiple alignment, 1395 have not shown a huge variation of the consensus secondary structure reported by the family in comparison to the corrected alignment, generated in this methodology (see Figure S9). The *tree edit distance* ($e_{distance}$) calculated with the `Vienna RNA Package` [?], was used to measure this variation. The complement of this group is composed by a set of 255 *loci* that have mature

annotation, but impaired the alignment conserved blocks. Due to the last classification, the final list of homologous miRNA on *D. vexillum* were categorized based on the secondary structure variation respect to the correspondent stockholm alignment family as follows: *high* ($e_{distance} = 0, 2$), *medium* ($e_{distance} = 3, 5$), and *low* ($e_{distance} = 6, 7$) and *no fitting* ($e_{distance} > 7$). As an example, Figure S10 shows examples for each category to illustrate the conservation degree respect to the current corrected multiple alignments from RFAM.

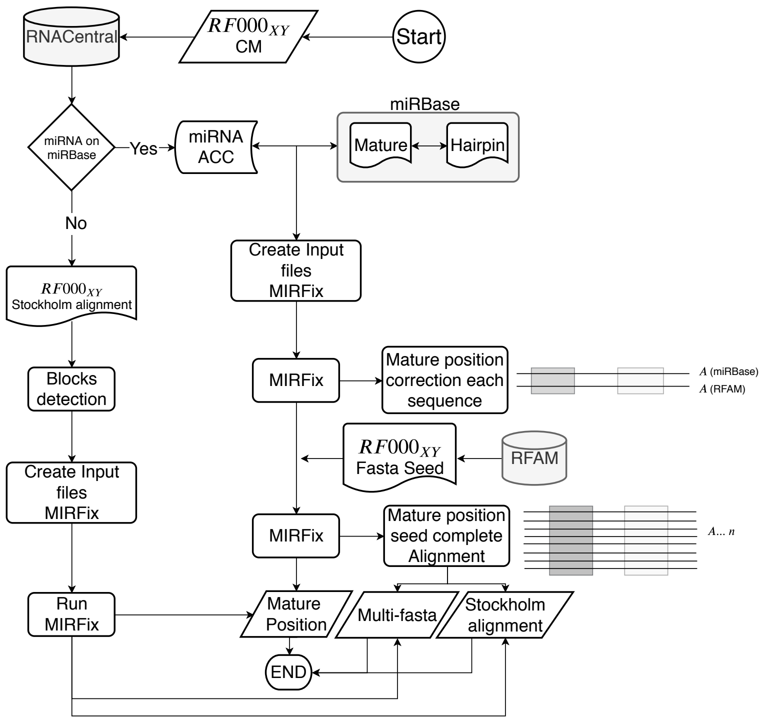

Figure S 9: Summary of annotation of mature sequences methods. Starting with CM seed sequences from `RFAM` and based on their databases annotation (on `RNACentral` and `miRBase`), hairpins are classified and analyzed, as described earlier. After some processing, reported mature positions are corrected using `MIRfix`. Final output consists on the position of the mature inside its harboring hairpin, the corrected fasta sequences and a structural alignment of the miRNA family. Inside the example A represents a sequence inside the structural alignment.

RMST annotation

Ten RMST covariance models (RF01962-RF01971) were retrieved from `RFAM` v.14 with `cmfetch`. Using `cmsearch` as described on command line methods on Section S12, on selected genomes of chordates (*B. floridae* (Brfl), *B. belcheri* (Brbe), *O. dioica* (Oidi), *M. occidentalis* (Mlis), *M. oculata* (Mata), *M. occulta* (Mlta), *B. schlosseri* (Bosc), *H. roretzi* (Haro), *S. thompsoni* (Sath), *B. leachii* (Bole), *D. vexillum* (Dive), *C. robusta* (Ciro), *C. savignyi* (Cisa), *P. marinus* (Pema), *D. rerio* (Dare), *L. chalumnae* (Lach), *M. musculus* (Mumu) and *H. sapiens* (Hosa)), echinoderms (*S. purpuratus* (Stpu) and *P. miniata* (Pami)) and hemichordata (*S. kowalevskii* (Sako)). True candidates were retrieved if reported an E-value $\leq 10^{-3}$ and the 32% of the *gathering score*. The final result is show in Figure S12, the distribution of RMST families cover all the evaluated species.

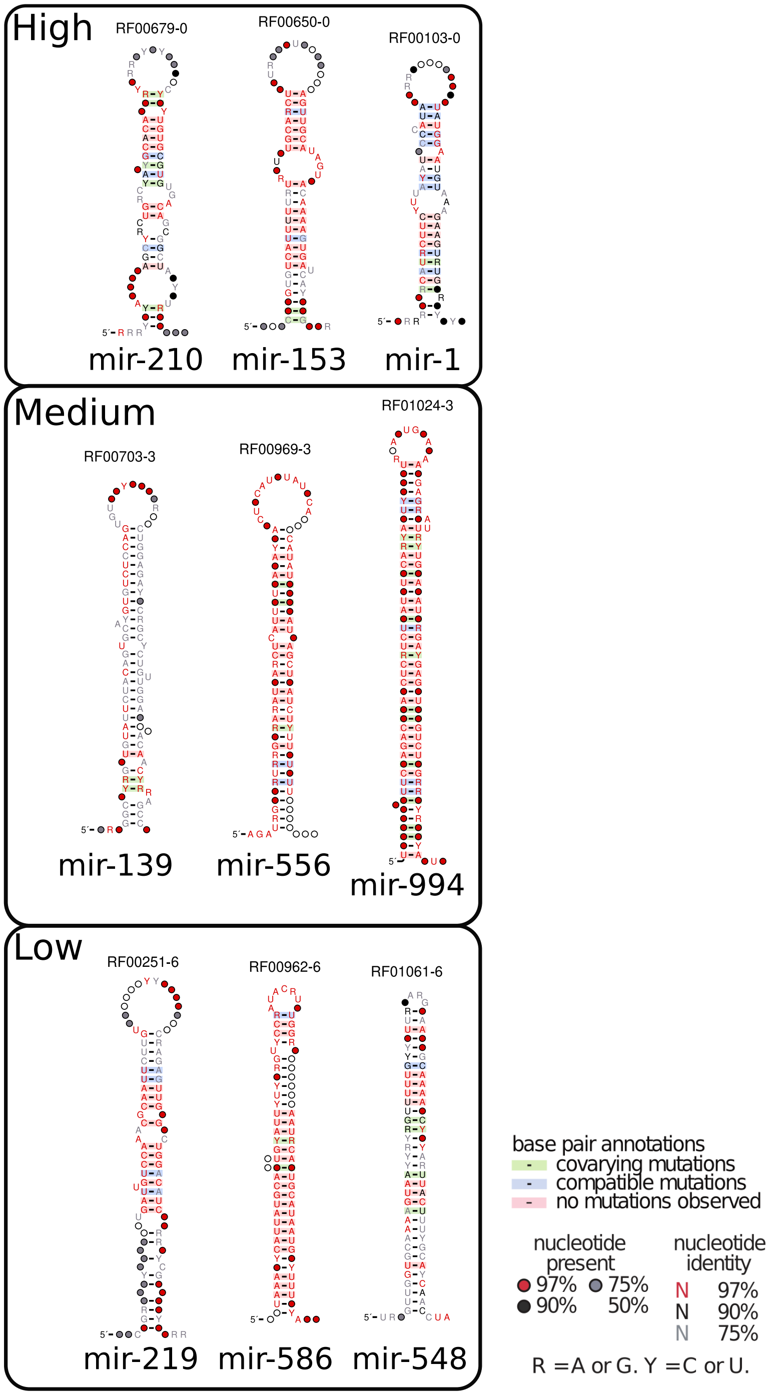

Figure S 10: Examples of miRNA candidates detected and validated on *D. vexillum*. Labels (*High*, *Medium* and *Low*) refer to the *tree edit distance* calculated for the reported secondary structure from reported `RFAM` alignments and the same structure including the detected miRNA of the same family as explained on Figure S9. Secondary structures were generated with `R2R` [**?**], which provides annotations for sequence and structure conservation, as detailed in the provided legend.

7 Detection and analysis of repeat elements

Detection of repeated sequences on the *D. vexillum* genome was completed based on the work flow

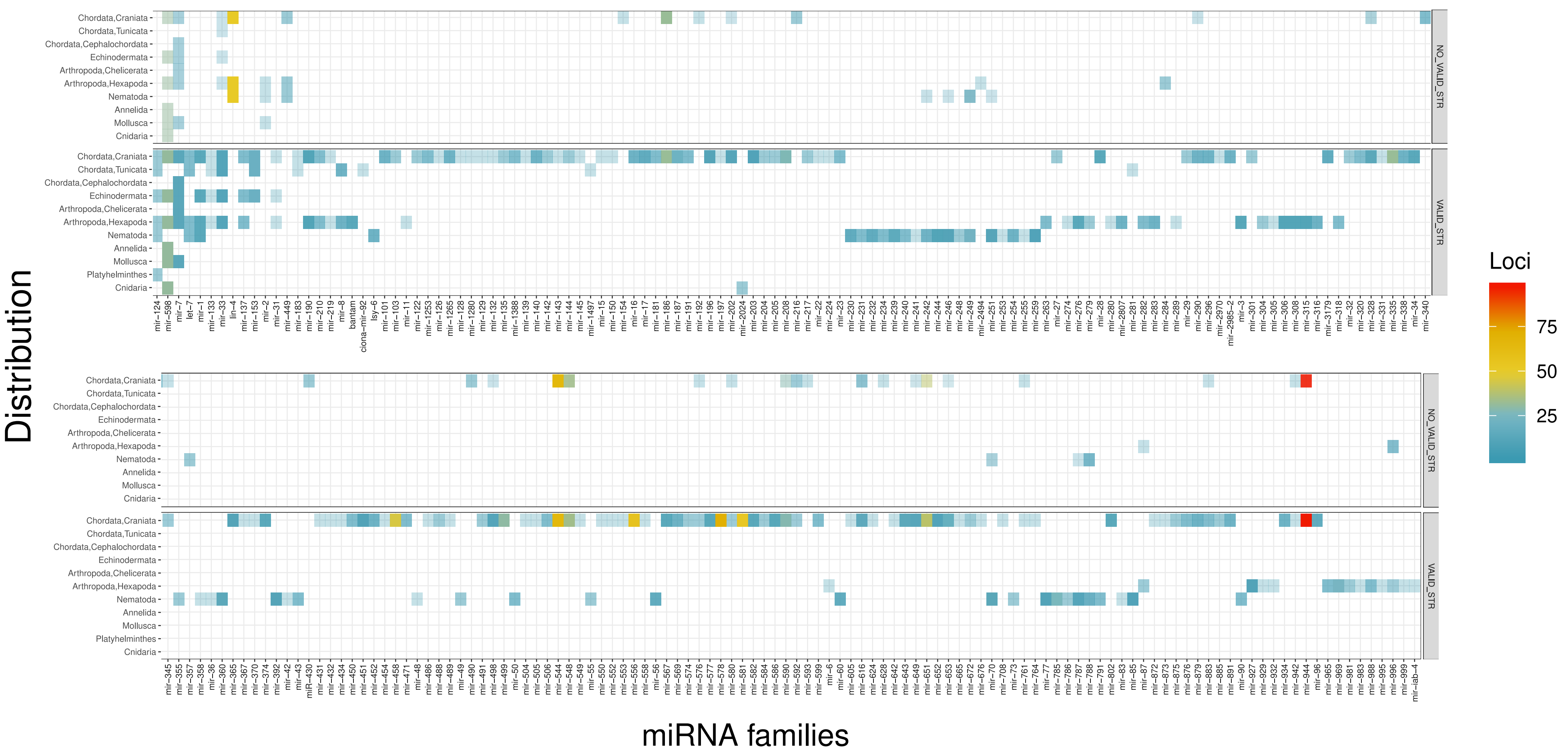

Figure S 11: Distribution of final set of miRNA families annotated on *D. vexillum*. Distribution axis corresponds to the taxonomic classification for the species that were reported on the `stockholm` seed alignment for each miRNA family by `RFAM`. Each family has two groups, represented by the divided panels, *VALID_STR* and *NO_VALID_STR* which contains those *loci* that fit into the alignment or not, respectively. The *loci* count were performed for each family and for each panel. As a consequence, *loci* numbers are accounted by columns without considering rows.

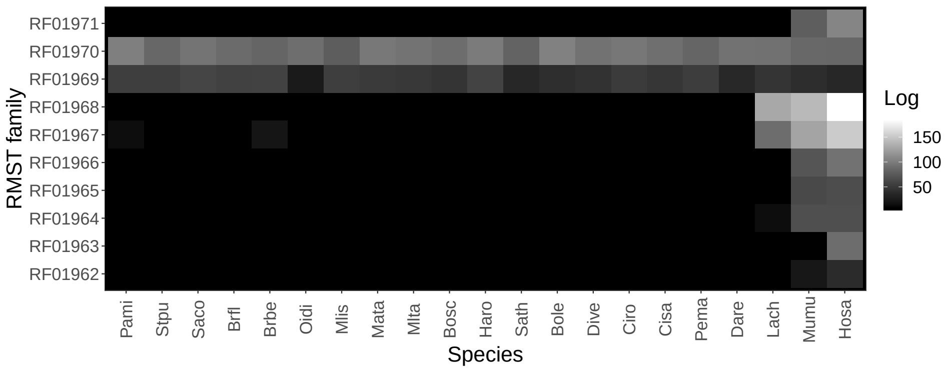

Figure S 12: Distribution of RMST families along hemichordates, echinoderms and chordates. E-value are represented as Log = $\max(-\log_2(E_{value}))$, white colors corresponds to lower E-values, black squares indicate values close to 0. Name tags are described on main text. Genomes from *M. musculus* (GRCm38) and *H. sapiens* (GRCh38) were retrieved from Ensembl v.96.

described in Figure S13. As explained on the main text, this automated strategy comprises the construction of a *de novo* repeat library. With this new specie specific repeat library, were performed the masking and searching steps. The final output of this strategy is composed by *de novo* library and a GFF3 file, which describe the genome coordinates of detected repeated elements and could be accessed on the genome browser from *D. vexillum*, described in this study.

In detail, most abundant families are: Unknown, Simple repeat, SINE/tRNA-Lys, LINE/L2, DNA/hAT-Charlie, Low complexity and LINE/Penelope, which have been compared against current available reported number of repetitive elements on tunicates (retrieved from [**?**]). The complete comparative comparison is described on Table S9.

Table S 9: Comparison of the percentage of the 7 most common repetitive elements detected in *D. vexillum* and other 8 tunicate species reported in [**?**]. Species tags are: **Bole**: *B. leachii* **Bosc**: *B. schlosseri*, **Ciro**: *C. robusta*, **Cisa**: *C. savignyi*, **Mlis**: *M. occidentalis*, **Mlta**: *M. oculata*, **Mata**: *M. oculata*, **Oidi**: *O. dioica*, **Dive**: *D. vexillum*.

**Repeat Family**	**Bole**	**Bosc**	**Ciro**	**Cisa**	**Mlis**	**Mlta**	**Mata**	**Oidi**	**Dive**
Unknown	17.39	45.58	20.57	20.69	23.19	17.75	20.20	11.57	35.96
Simple Repeat	1.52	4.12	0.64	0.56	0.98	0.70	0.92	1.22	0.82
SINE/tRNA-Lys	NA	NA	NA	NA	NA	NA	NA	NA	4.89
LINE/L2	0.07	1.49	0.01	0.36	0.21	0.28	0.63	NA	3.98
DNA/hAT-Charlie	0.04	2.46	0.01	0.10	NA	NA	0.01	NA	3.8
Low complexity	0.02	0.01	0.15	0.11	0.28	0.16	0.13	0.14	0.25
LINE/Penelope	0.13	0.31	< 0.001	NA	0.18	0.02	0.12	0.21	0.98

By coordinates analysis were detected some repetitions that overlap their reported coordinates with ncRNA elements, that have been detected in this study. In this case, from the total number of repeated elements, 0.746% (10,325) were found with overlapping regions. Inside this set of regions, more than 88.98% are composed by masked tRNAs, 9.06% miRNAs and the 1.96% remaining are represented by *cis*-regulatory, ribozymes, rRNAs, snoRNAs and snRNAs. For all ncRNAs families previously mentioned, the most common repeat family is *Unknown*, except for rRNAs. Apart from this repetitive set of elements, for *cis*-regulatory sequences are simple repeats and DNA/PIF-Harbinger, for miRNAs LINE/Penelope and for tRNAs SINE/tRNA-Lys. For rRNAs and snRNAs were detected common regions that are identified

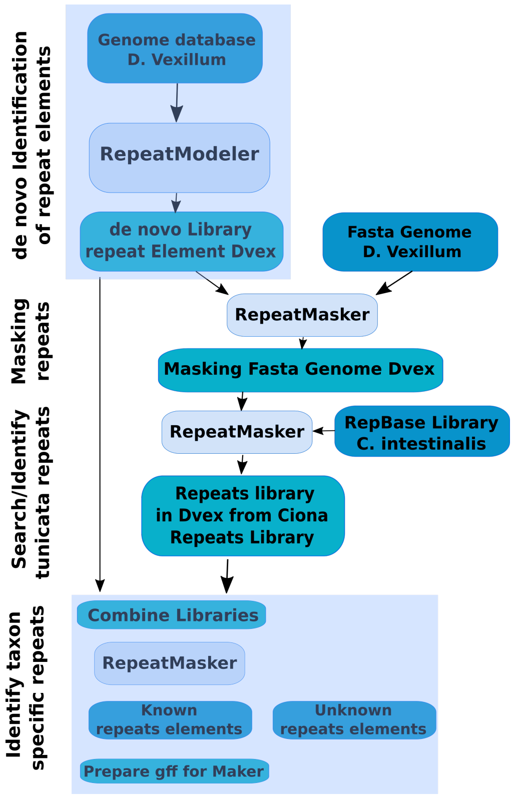

Figure S 13: Repeat detection work flow on *D. vexillum* genome.

as a ncRNAs by the repeat database. As a summary, Table S10, details the annotated ncRNAs that share an overlapping location with a detected repetitive element.

Table S 10: Annotated ncRNAs families and *loci* (in parentheses) on *D. vexillum* genome. The final number of ncRNAs are addressed in the *Final* column and the corresponding annotation that shared genomic region with repetitive elements are shown in the *Masked* column.

**ncRNA Family**	**Final**	**Masked**
Cis-Reg	3 (333)	1 (161)
miRNAs	265 (2084)	192 (935)
misc RNAs	2 (2)	0 (0)
lncRNAs	2 (8)	0 (0)
Ribozyme	3 (11)	1 (2)
rRNAs	4 (84)	3 (11)
snoRNAs	12 (18)	2 (2)
snRNAs	11 (121)	6 (27)
tRNAs	23 (2701)	20 (1293)
Total	303 (5362)	205 (2431)

8 Mitochondrial DNA annotation

Based on the mapping methodology explained in Materials and Methods, mitochondrial DNA from *D. vexillum* was identified on the scaffold: `scaffold1656-size16126`. Final annotation is described on Figure S15.

In order to test if exists possible mt-DNA arrangements or a possible assembly error, 8 Tunicata reported mt-DNA genomes were retrieved (accession numbers: NC_017929.1, NC_034372.1, NC_004570.1, NC_009833.1, NC_009834.1, NC_012887.1, NC_024105.1, NC_026107.1; which corresponds to the species: *C. intestinalis*, *C. robusta*, *C. savignyi*, *P. mammillata*, *P. fumigata*, *C. lepadiformis*), *C. phlegraea*, *D. vexillum* isolate clade A, respectively, from `NCBI` by a `Perl` script using the `Bio::DB::GenBank` library. Multiple genome alignment was performed with the described mt-DNA genomes with `progressiveMauve` [?], command line described on Section S12. Resulting alignment blocks along all the subject species is depicted on Figure S14.

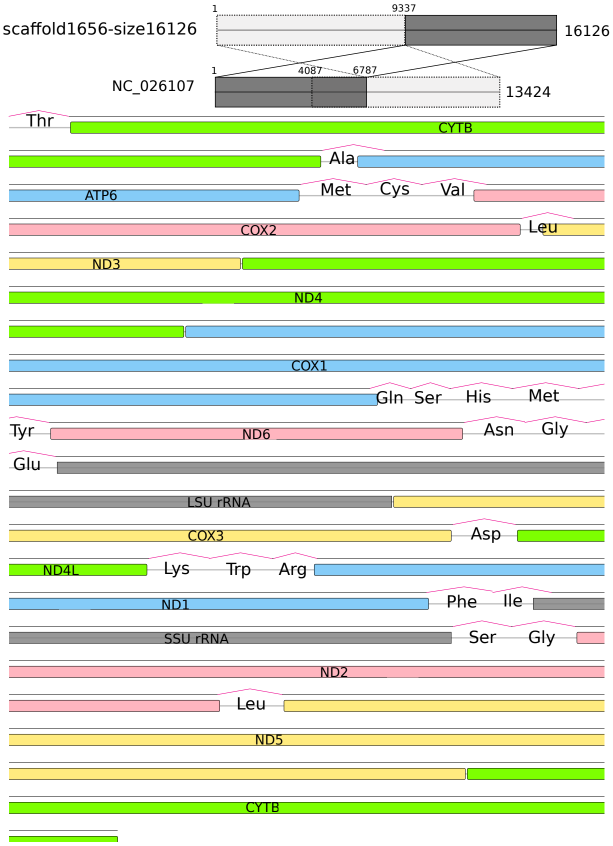

Figure S 14: Mitochondrial genome from *D. vexillum*. **A** Pairwise alignment between the newly assembled mtDNA (located on scaffold scaffold1656-size16126) and the reported mtDNA (Accession number:NC_026107). **B** Distribution of reported sequences on the newly assembled mtDNA. For practical means, inter-genic regions were not considered. Genes sizes and order is shown, including the tRNA elements (red bends) and rRNA (gray boxes).

9 Determination of covariance models thresholds

The proposed methodology on [?], allowed to detect in this study for the most conserved miRNAs families along the *D. vexillum* genome. In order to validate this methodology, 37 miRNAs reported for *Halocynthia roretzi* genome [?] were validated using the reported CM for miRNAs. At the end,

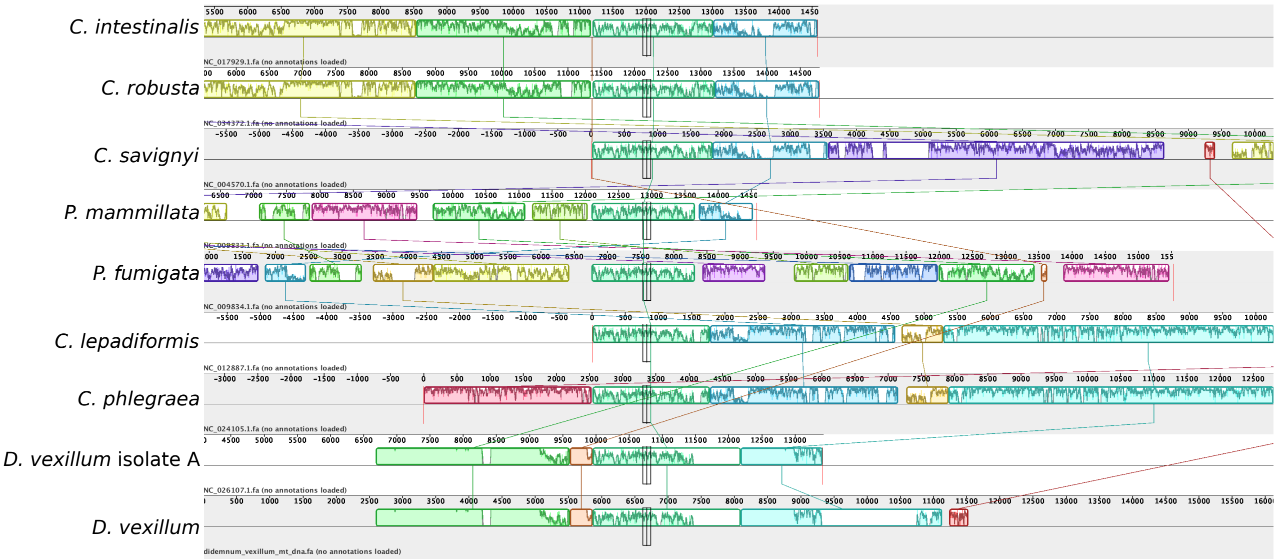

Figure S 15: Graphic representation of a mitochondrial genome multiple alignment. Tunicata mt-DNA is represented along a coordinate system, which start from 0 to the length of the mt-DNA. The resulting alignment is centered on the conserved block along all genomes, which overlaps with the position of mt-LSU. Other conserved blocks have been detected and are highlighted by the same color and the same corresponding joining line. Negative numbers along the coordinates are useless in terms of distances, otherwise these refer to a translocated region (i.e. *Cionas* comparison).

11 miRNAs *loci* were identified, next the comparison with the reported miRNAs results in 9 *loci*, the remaining ones (2) were not identified in [**?**]. Conserved identified candidates belong from let-7, mir-33, mir-124, mir-133 and mir-219, and the missing ones belongs from mir-10 family. Given the last results, a manual inspection methodology allowed to determine the origin of this mis-annotation. In this case, the missing candidates were classified in the applied methodology as false candidates because their `bitscore` values are not greater than default covariance model `gathering score` *GA* provided by `RFAM`. This is a sensitivity bias that could occur in some structural alignments and could be detected applying some specific parameters in `cmsearch/cmscan` in order to identify those candidates, as described by [**?**]. In this case, reported fasta sequences from precursor miRNAs were retrieved from the *H. roretzi* genome (v.1), organized in a multifasta file. This input file was subject to structural evaluations with `cmsearch`, as described in command line methods (Section S12). At the same time, to compare those results, it was necessary to create Positive and Negative control sets, as described below.

Control positive sequences were retrieved from `MirGeneDB` [**?**] and it was composed by all the reported sequences in the database with additional 30 flanking nucleotides[3], obtaining 8656 from 8847 sequences that reported a miRNA family annotation (discarding 'novel' families). Control false sequences were generated from the reported CDS sequences from human genome (v.GRCh38) retrieved from `Ensembl` [4]. The sequences lengths were calculated and with sequence identifiers was generated a table, which was selected those sequences that reported lengths between 80 and 150 nucleotides. Next, those selected candidates were sampled randomly (with replacement, 95% of confidence, 5% of confidence interval and a total of 4694 sequences) to create random seed groups; this sampling methodology was replicated 10 times. In order to shuffle the nucleotides inside those random seed groups, `shuffleseq` from `EMBOSS:6.6.0.0` [**?**] was applied, generating 100 shuffling steps on the query sequences[5]. In order to analyze the distribution patterns of bitscore, it was necessary to normalize those values because GA scores are covariance model

[3] `http://mirgenedb.org/static/data/ALL/ALL--pri-30-30.fas`

[4] `http://ftp.ensembl.org/pub/current_gtf/homo_sapiens/Homo_sapiens.GRCh38.cds.all.fa`

[5] `shuffleseq -sequence input.fa -out output.fa -shuffle 100`

specific. In this case, normalization of `bitscores` (nGA) was performed as referenced in Equation 1.

$$nGA = b/GA \tag{1}$$

Where, b corresponds to reported `bitscore` from `cmsearch` result and GA is the provided GA score from RFAM.

The reported distribution for all the proposed treatments is described in Figure S16. Those results correspond to the density distribution of nGA scores, which x intercept ($x = 1.0$) specify the threshold value that had been selected on [**?**] as bitscore filter or as defined by RFAM the GA score. Then, following those filters for miRNA sequences a lot of true validated sequences were discarded as seen in the multimodal distribution of the positive control group with one peak in ~ 0.25 and another in ~ 1.835. Specifically for this positive control group, Figure S17, the distribution density of nGA in shows a range from 0 to 2.72 and to identify the best strategy to filter those results, it is necessary to define a threshold value based on the nGA. Suggested threshold, as indicated early, $nGA = 1.0$ ensure the identification of most similar structural folds in respect to the CM. This works mostly for the most conserved sequences, but it is not enough to get the complete spectra of true candidates. For that reason, the classified 'Same' candidates (candidates that shown a successful annotation with the correct CM, in Figure S17A) were depicted on Figure S17B. The suggested criteria from `infernal` based on the $E - value$ threshold, still shows a possible subset of good candidates that are reported in a range of $nGA = (0.298, 2.516)$.

The negative control reported a leptokurtic distribution ($kurtosis = 5.24$) and positive skewed ($skewness = 1.054$) were the only reported peak is about ~ 0.15 and also the maximum reported value is 0.438. When the discrimination criteria from `cmsearch` is considered, two different distributions are evident: those candidates that reported an $E - value \leq 0.01$ (4539) distribute with a $\mu = 0.1492 \pm 0.054$. Few candidates (5), reported greater E-values, but those candidates did not exceed the default threshold value in $nGA = 1.0$, as shown in Figure S18.

Based on the last results, it is necessary to modify the nGA threshold in order to consider a broader range of possible true candidates in a classification process, specifically from miRNAs. As noted earlier, nGA threshold should be reduced to maximize the number of identified true candidates. In this way, it is required that true candidates report both scores: lower $E - value \leq 0.01$ and at the same time nGA than true negative control distribution as shown in Figure S18 as '?'. According to the distribution of this negative control data set, the true negative ones reported a confidence Interval ($CI = 0.012$, $\alpha = 0.05$, $\mu = 0.367$) and in general, the Control Negative ($CI = 0.002$, $\alpha = 0.05$, $\mu = 0.149$). Assuming that the reported miRNAs on *H. roretzi* are effectively miRNAs, the value of nGA was evaluated in comparison to the absolute frequency of true candidates. The threshold was defined based on the last negative distribution, and the number of successful candidates annotated. In conclusion, as shown in Figure S19, the selected value for this threshold was defined as $nGA = 0.32$.

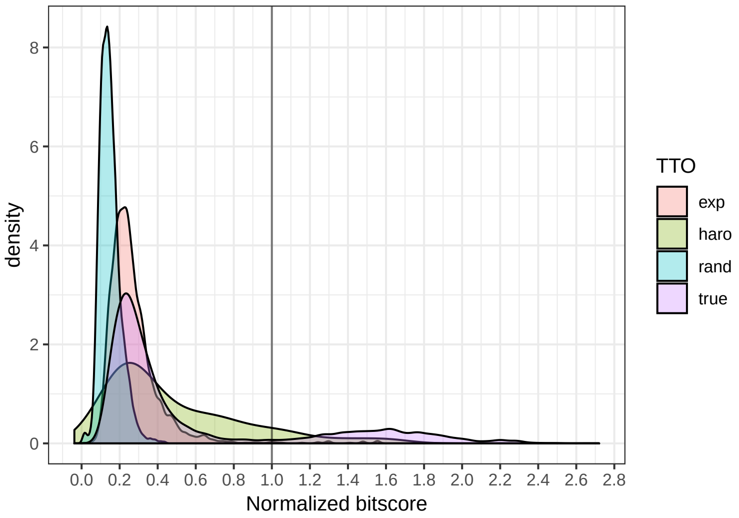

Figure S 16: Density distribution of normalized bitscore (nGA). **exp** corresponds to results from structural alignments on *C. robusta* candidates with the `blastn` number 4, described in this study, it constitutes an 'external' group of candidates, **haro** represents evaluation of annotated candidates on *H. roretzi* genome [**?**], **rand** is the generated negative control and **true** the results from sequence retrieved from `MirGeneDB` [**?**].

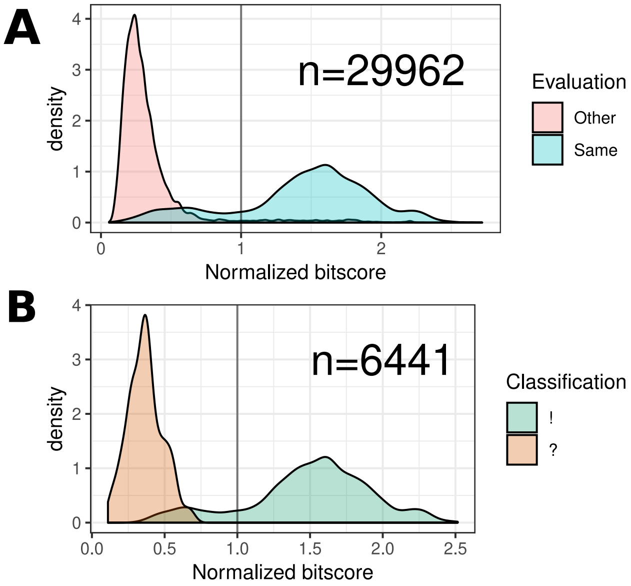

Figure S 17: Density distribution of Control Positive sequences. **A.** Depicts the distribution of *nGA* from control positive sequences. Previous structural evaluation in this control set was possible to know the annotated miRNA family, in this case 'Same' corresponds to the evaluation with the same covariance model with the annotated family. The final evaluated number of results was 29,962, where 6441 were evaluated with the same miRNA covariance model and the rest 23,521 not. From the 'Same' group, in **B**, was classified this density distribution by the criteria from `cmsearch`, based on the default 'inclusion threshold' (E-value ≤ 0.01).

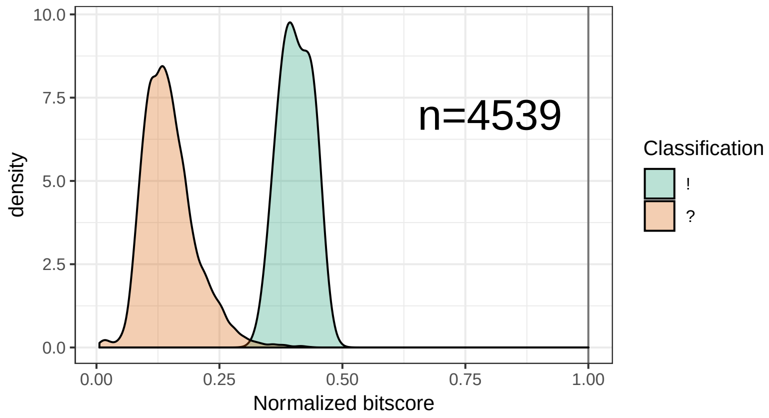

Figure S 18: Density distribution of Negative control sequences.

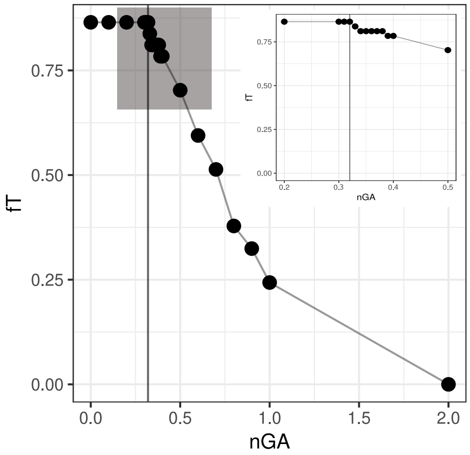

Figure S 19: Evaluation of nGA on reported candidates from *H. roretzi*[**?**]. fT corresponds to the absolute frequency of the classified true candidates with the same annotated miRNA family and covariance model. nGA represents the threshold nGA value applied to classify the candidates. The frequency was calculated on all the reported conserved candidates from *H. roretzi* (n=37). From this set, 4 families does not have a correspondent name on RFAM miRNA families (miR-3876, miR-3182, miR-3598 and miR-1502). Selected new threshold (= 0.32) is depicted as an intercept on x axis.

10 Close homology relations from *D. vexillum* proteins

The annotated protein dataset from *D. vexillum* (64,424 proteins) was compared to multiple protein reference datasets to quantify the number of proteins that could be supported using direct sources of close homology, as follows:

- Comparison to *C. robusta* proteins: Taking as a reference the collapsed the largest protein product for each gene to obtain 14,072 non-*ab-initio* dataset from the solitary tunicate *C. robusta* [?][6]. `proteinortho` v.6.0.28 [?] was used to detect the orthologs between both species.

- Comparison to ortholog clusters from `eggNOG` database [?] v.5, calculated in this study as described in Supp. File 3 using `eggNOG-Mapper` [?].

- Detection of close homologs in comparison to non-redundant database from NCBI (`nr`): using `blastp`. Taxonomy annotation of the best candidate was obtained using `ete3` [?].

The ∼ 42% (27,032) of *D. vexillum* proteins were detected with a valid homology relation with at least one of the described comparisons. As referred in Figure S20, most of the proteins reported a match with metazoan proteins 26,005 (all metazoa matches including the orthologs with *C. robusta*), another set of 19 proteins overlapped with functional annotation related with orthologs groups annotated in a few metazoan species as the tunicate *C. savignyi* or the sea urchin (*S. purpuratus*). The remaining 1008 proteins had close homologs with a non-metazoan species, mostly from Bacteria (Bacterioidetes/Chlorobi group and Gammaproteobacteria) and Fungi (see Figure S21).

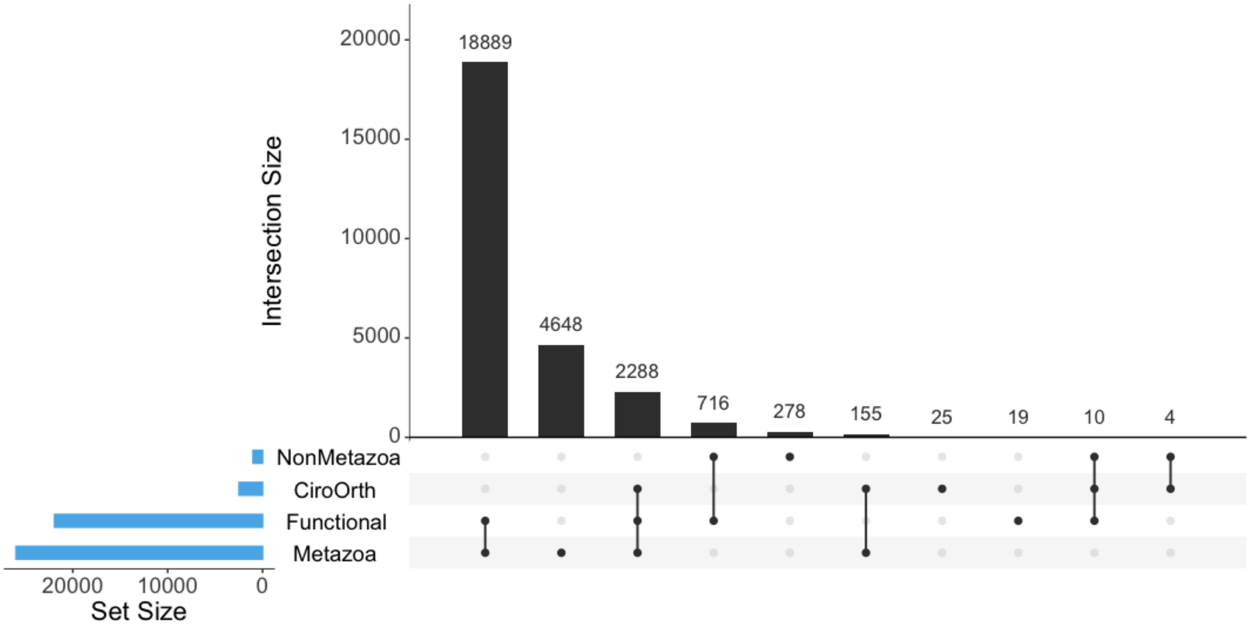

Figure S 20: Distribution of homology relations of *D. vexillum* proteins.

The remaining 58% (37392 proteins) did not report a valid candidate through evaluated strategies, even with relaxed `blastp` parameters. Only 235 proteins reported complete valid annotations of `Pfam` protein domains (through a homology strategy using `Pfam-A` database and `hmmsearch`). As a complement, `Maker` evidence to build gene models were considered to improve the evaluation of annotated proteins, as suggested in [?]. We identified that 28,431 protein-coding mRNAs did not have annotation/have an

[6] `http://ghost.zool.kyoto-u.ac.jp/datas/HT.KYGene.nonabmodels.protein.fasta.zip`

incomplete annotation at UTR level and at the same time, the $\sim 10.6\%$ of the corresponding annotated genes in this group were annotated as isolated, with coverage $> 50\%$ in comparison to their annotated scaffold. Looking for the annotation model's support data, 24,762 mRNAs were annotated using annotated proteins and/or with *ab initio* models, but not by the transcriptome data generated in this study. Despite 5977 protein-coding transcripts were supported by mRNA-seq data and their UTR were annotated, was not possible to identify clear homologs.

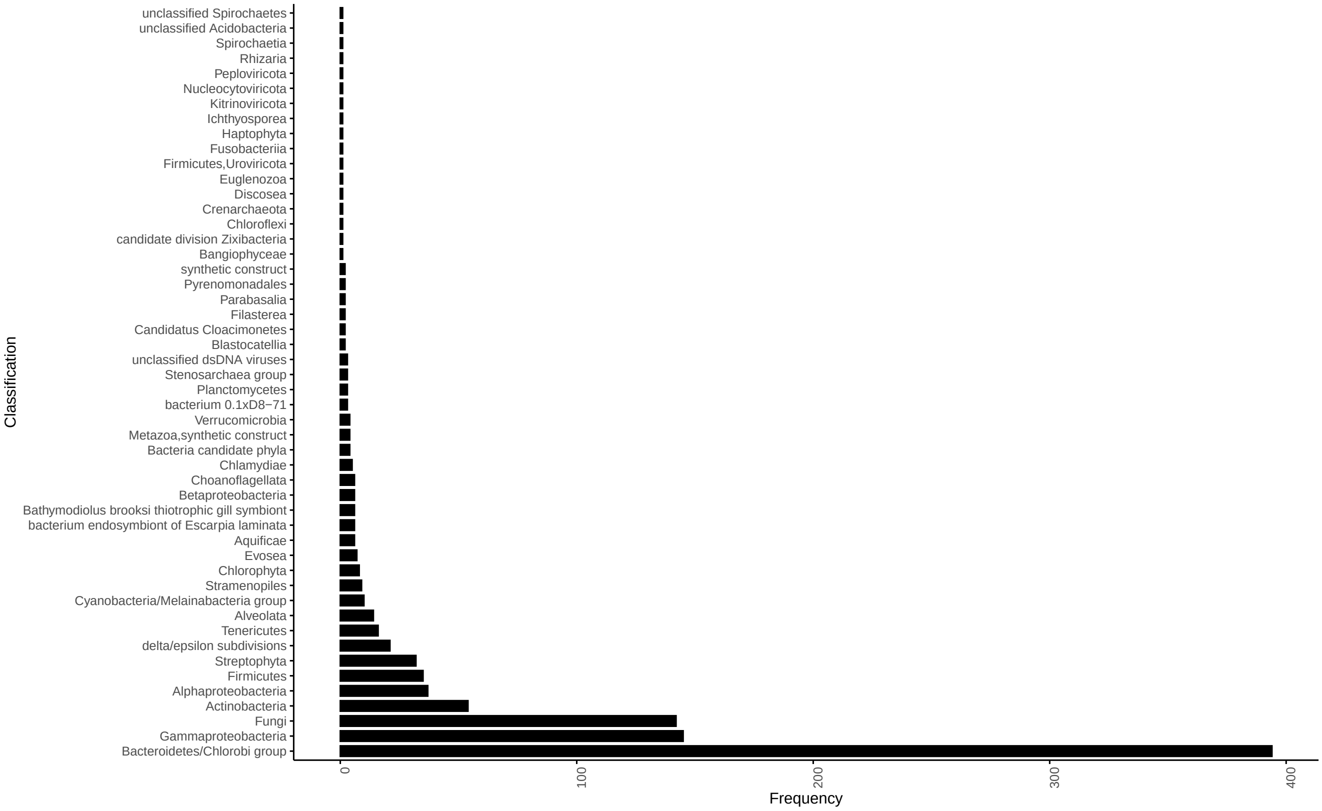

Figure S 21: Number of *D. vexillum* proteins with close homologs in non-metazoan species. $N = 1008$.

Evidence of gene fragmentation

Figure S22A shows the protein `KY.Chr14.999.v1.SL1-1`, split in 4 *D. vexillum* proteins (`Dvex_pep38108`, `Dvex_38109`, `Dvex_pep38110`, and `Dvex_48617`), as protein products from genes annotated in different scaffolds. Additionally, the protein `KY.Chr1.580.v1.ND1-1`, was recognized as *fragmented* and dispersed on 3 *D. vexillum* proteins (`Dvex_pep12273`, `Dvex_61519`, and `Dvex_pep36022`). Finally, in Figure S22B the protein `KY.Chr11.363.v1.SL1-1` was identified as a *1:1* on *D. vexillum*, but incomplete.

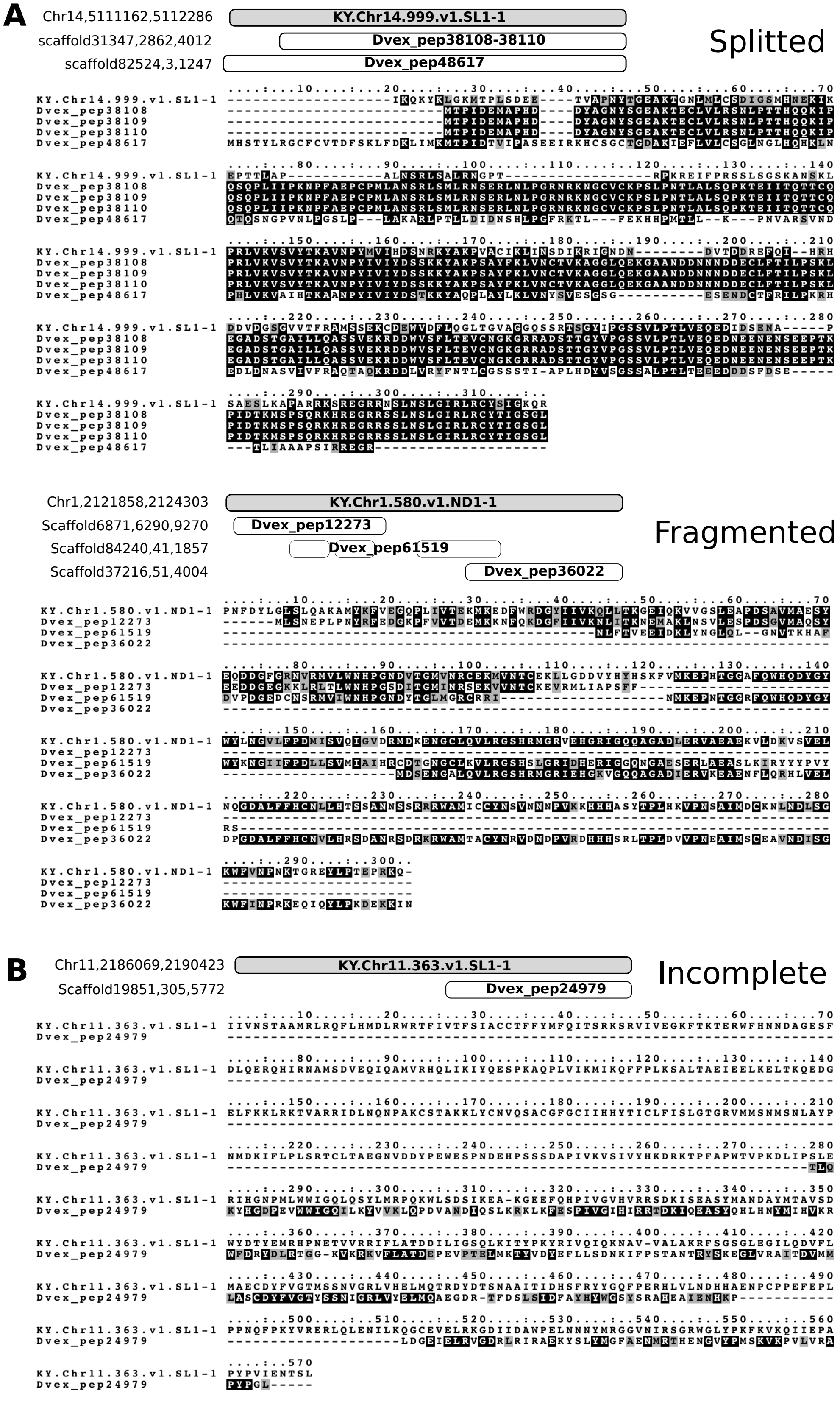

Figure S 22: Multiple protein alignments showing found relations between *C. robusta* and *D. vexillum* proteins: **A.** Complete (Fragmented and Splitted) and **B.** Incomplete. Alignment representation for each case is represented, *Ciona* protein colored in gray. Protein-coding gene coordinates are described on figure left side.

11 Ontology annotation and enrichment analysis on protein ortholog groups

The annotated set of proteins reported on *D. vexillum*, *B. floridae*, *B. leachii*, *B. schlosseri*, *C. robusta*, *C. savignyi*, *L. chalumnae*, *M. oculata*, *M. occidentalis*, *O. dioica* and *P. marinus*, were mapped to the database `eggNOG` v.5 [?] against all the pre-calculated orthology groups, using `eggNOG-Mapper` [?], as referred in Section S12. Only 1 : 1 orthologs were considered to posterior analysis. Consequently, the annotation and the ontologies assignments were transferred to the detected ortholog protein in the reference species. Absence/presence analysis for those orthology groups requires that for the defined set of reference species, some subsets of species had to be defined, based on the phylogenetic classification (i.e. Tunicata) or specific losses on a clade (i.e. Loss Tunicata) as follows:

- Cephalochordata (C): *B. floridae.*
- Tunicata (T): *C. savignyi*, *C. robusta*, *M. oculata*, *M. occidentalis*, *O. dioica*, *B. schlosseri*, *B. leachii* and *D. vexillum.*
- Vertebrata (V): *P. marinus* and *L. chalumnae.*
- Loss Vertebrata: $\exists x \in (C \vee T)$.
- Olfactores: $\exists x \in (T \vee V)$.
- Loss Tunicata: $\exists! x \in T \vee \exists x \in (C \vee V)$.
- Chordata: $\exists x \in (C \vee T \vee V)$.

Enrichment analysis using Fisher's exact test with Benjamini-Hochberg multiple test correction on p-values was calculated using `goatools` [?] based on protein counts. For details, please refer to the command line methods on Section S12.

11.1 Results

The total number of detected orthology groups was 8033. As depicted on Figure S23A, the absolute frequency of groups of clusters are compared against the number of species involved in these earlier defined groups. The highest frequencies are reported on orthology groups that mapped proteins from one species, in this case these results belong from Cephalochordata or Vertebrata. The number of orthologous proteins clearly increases with the number of species as show on Figure S23B. Based on the last results, is possible to define a conserved set of proteins that are part of the Chordata subset that reported a high number of proteins inside the identified orthology groups.

11.1.1 Additional comparisons of shared ortholog groups

Additional shared set, composed by 5 orthology groups, was found when *O. dioica* was excluded. From those groups, only one did not have functional annotation and the other 4 revealed functional annotations related to *dopamine monooxygenase activity* (ENOG502C4CE, 33), *regulatory subunits of protein phosphatases* (ENOG502CZS6, 17), the *reducing fluoride concentration levels in the cell* (COG0239, 15) and *AMP binding* (COG0589, 71).

Five orthology groups were found exclusively in all three available colonial tunicate genomes (*B. schlosseri*, *B. leachii* and *D. vexillum*), and 21 orthology groups were shared between *D. vexillum* and at least one other colonial botryllid. The set of orthology groups shared by all colonial ascidian genomes (5) contain proteins that present the following shared domains: Methyltransferase domains

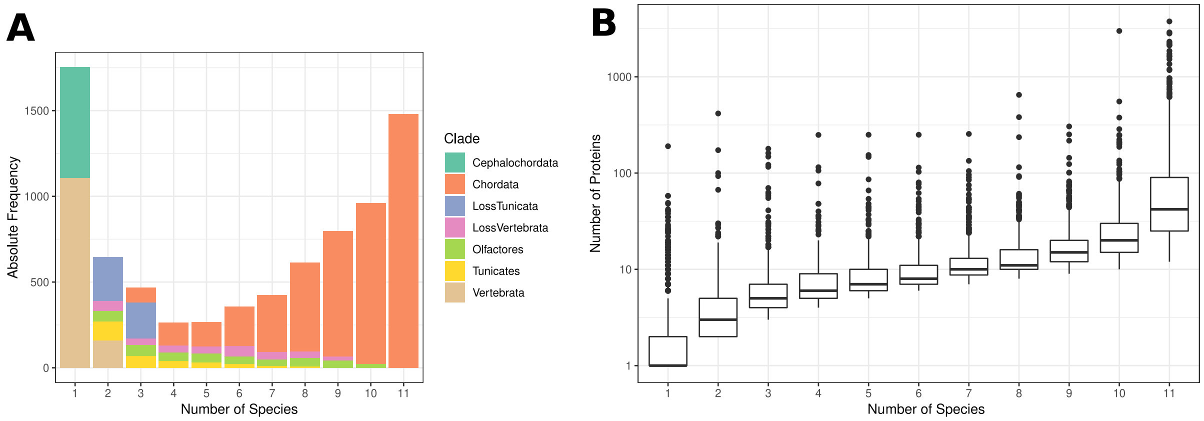

Figure S 23: Description of detected orthologs along chordate species. **A.** The number of detected proteins were classified according to the last defined clades. At the same time, the number of species that have an orthology candidates was considered. **B.** Number of proteins that have been found along `eggNOG` orthology groups. The results were discriminated based on the number of studied species that have reported orthologous sequences.

Table S 11: Orthology groups with the highest number of proteins that were missing on *D. vexillum*. Ortholog accession numbers correspond to the database `eggNOG`. Species labels are described on Section: RMST annotation.

**Group**	**Species**	**Ortholog group**	**No. Proteins**	**Annotation**	**Protein domains**
Loss Vertebrata	bole, bosc, brfl, ciro, cisa, mata, mlis	ENOG502EBW9	84	Unknown	Ferritin-like, TSP 1
Olfactores	bole, bosc, ciro, cisa, lach, mata, mlis	ENOG502CMYU	101	Unknown	DUF4371, Dimer Tnp hAT, zf-FCS
Chordata	bole, bosc, brfl, ciro, cisa, lach, mata, mlis, oidi, pema	ENOG5028MEB	168	galactosylceramide sulfotransferase activity	Galactose-3-O-sulfotransferase, Sulfotransfer 2

(ENOG502AI1Z), GIY-YIG endonucleases (ENOG502D72K), THAP DNA binding domains, DDE endonucleases (ENOG502E46Z), Antistasin domains (ENOG502FAER) and domains that lack annotation as the DUF3605 domain (ENOG502E7AU).

In contrast, no orthology groups were shared between all solitary tunicates (*C. robusta*, *C. savignyi*, *O. dioica*, *M. oculata* and *M. occidentalis*), but three were shared among the four solitary ascidians, excluding the larvacean. The orthology groups shared by all solitary ascidians included proteins and presented the following shared domains: (i) *Tumor necrosis factor receptor / nerve growth factor receptor repeats* (ENOG502D0E2); (ii) a group of proteins with the *BESS motif* (ENOG502DZH1), usually associated with DNA binding as molecular function (GO:0003677); and (iii) ARM-like, ARM-type fold and Rotatin domains of the Rotatin gene family members that have functions related to cilium organization.

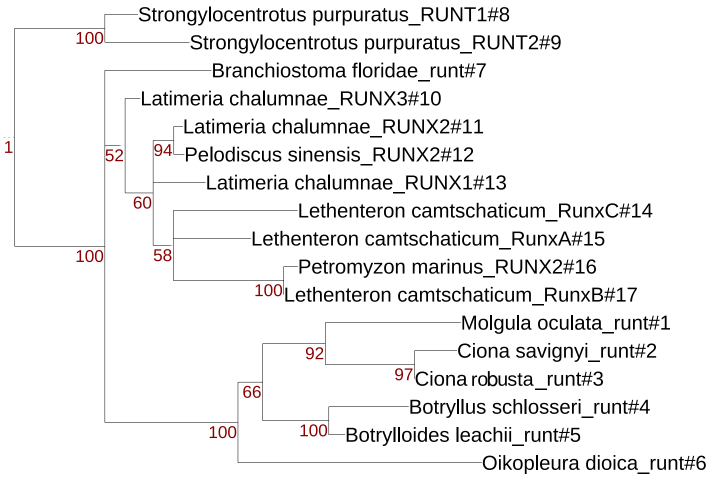

Figure S 24: Phylogenetic analisis of RUNX family.

11.1.2 Complete specific GO annotation on *D. vexillum*

The final list of GO terms specific to *D. vexillum* are depicted as a tree map in Figure S26. The final area is defined as $\log_{10} p-value_{adjusted}$ for each GO term after enrichment. The $p-value_{adjusted}$ was obtained from the `goatools`[?] results. As a result, enrichment analysis reported higher values on terms like *regulation* (positive regulation of phosphatidylinositol 3-kinase signaling, protein autophosphorylation, chaperone mediated protein folding) and *metabolism processes related with phosphorus.*

11.1.3 Shared enriched GO on chordata species

According to the previous grouping species definitions, common enriched GOs were depicted based on the resulting analysis from `REVIGO` [?]. For tunicates, colonial and solitary ones (Figure S29), Olfactores and vertebrates (Figure S30). Inside tunicates proteins, a lot of the enrichment referred to proteins that contribute into the *metabolic processes* (organic substances, macromolecules and nitrogen compounds). Other categories are specific to housekeeping process as: *transport, organization and regulation* (gene expression and maintenance of cell polarity). Specific processes have been identified for colonial tunicates related with *modifications of aminoacids, phosphorylation and response to stress.* In contrast, along solitary species the common GO are related with *biosynthesis of carbohydrate derivates.*

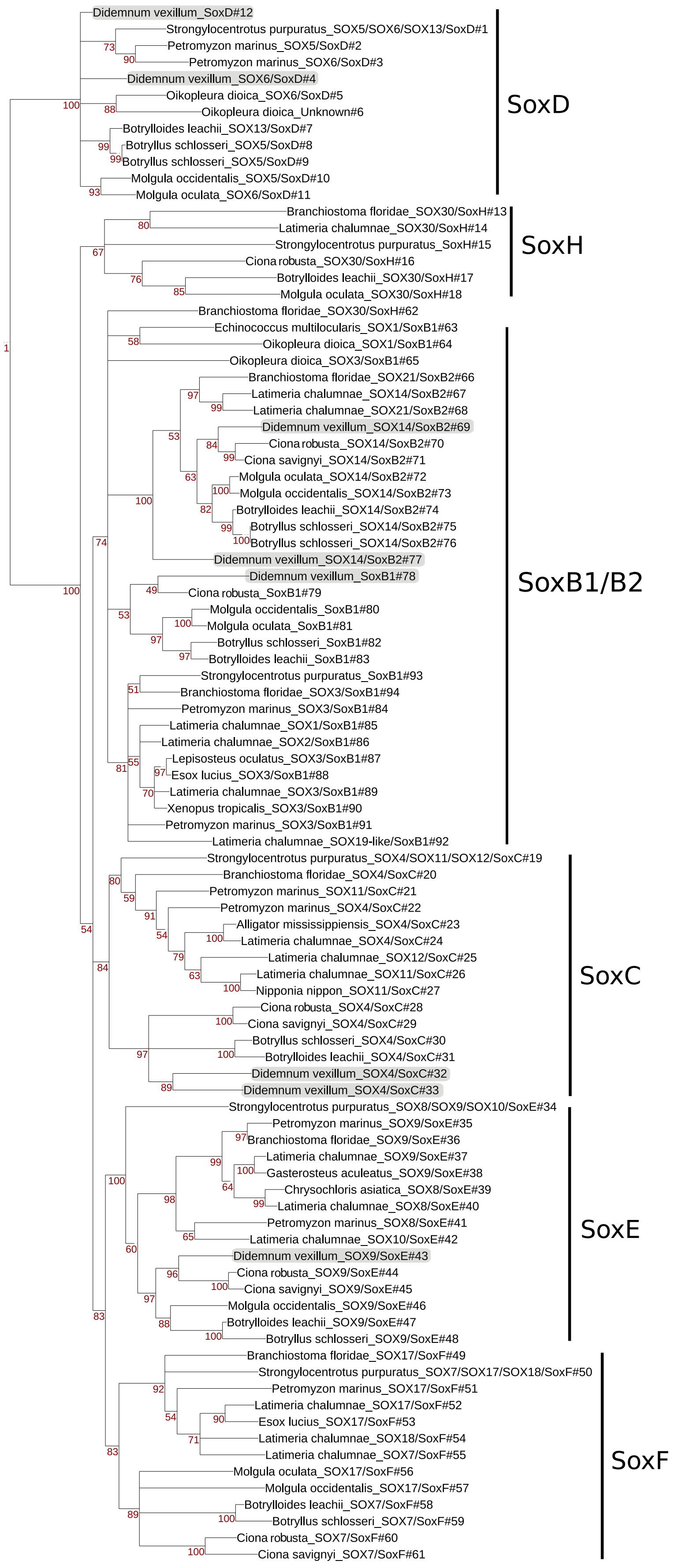

Figure S 25: Complete phylogenetic tree of the SOX family.

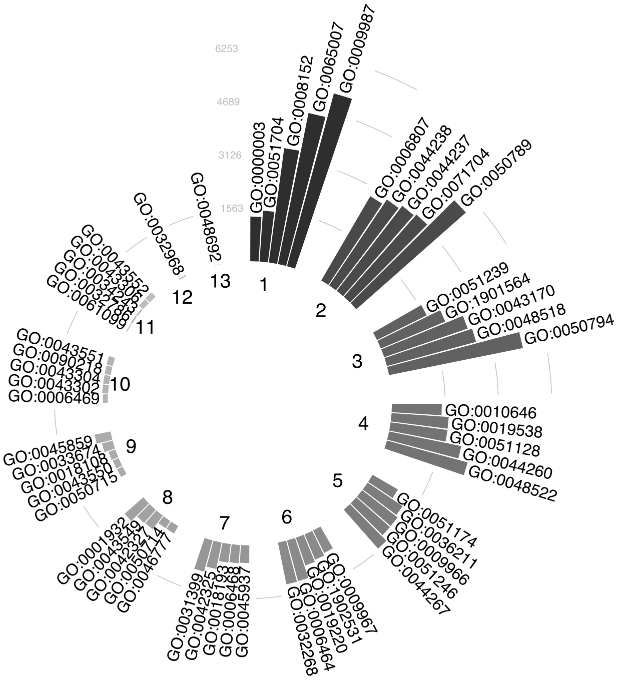

Depth	ID	Term	Count
1	GO:0065007	biological regulation	4520
1	GO:0009987	cellular process	5253
2	GO:0071704	organic substance metabolic process	3219
2	GO:0050789	regulation of biological process	4287
3	GO:0048518	positive regulation of biological process	2711
3	GO:0050794	regulation of cellular process	4007
4	GO:0044260	cellular macromolecule metabolic process	2161
4	GO:0048522	positive regulation of cellular process	2454
5	GO:0051246	regulation of protein metabolic process	1319
5	GO:0044267	cellular protein metabolic process	1519
6	GO:0006464	cellular protein modification process	1237
6	GO:0032268	regulation of cellular protein metabolic process	1249
7	GO:0042325	regulation of phosphorylation	790
7	GO:0031399	regulation of protein modification process	951
8	GO:0043549	regulation of kinase activity	514
8	GO:0001932	regulation of protein phosphorylation	730
9	GO:0033674	positive regulation of kinase activity	327
9	GO:0045859	regulation of protein kinase activity	478
10	GO:0090218	positive regulation of lipid kinase activity	176
10	GO:0043551	regulation of phosphatidylinositol 3−kinase activity	187
11	GO:0043306	positive regulation of mast cell degranulation	166
11	GO:0043552	positive regulation of phosphatidylinositol 3−kinase activity	175
12	GO:0032968	positive regulation of transcription elongation from RNA polymerase II promoter	43
13	GO:0048692	negative regulation of axon extension involved in regeneration	8

Figure S 26: Most frequent ontology terms in *D. vexillum* enriched orthology clusters. General categories were labeled with lower numbers whereas more specific categories with higher numbers.

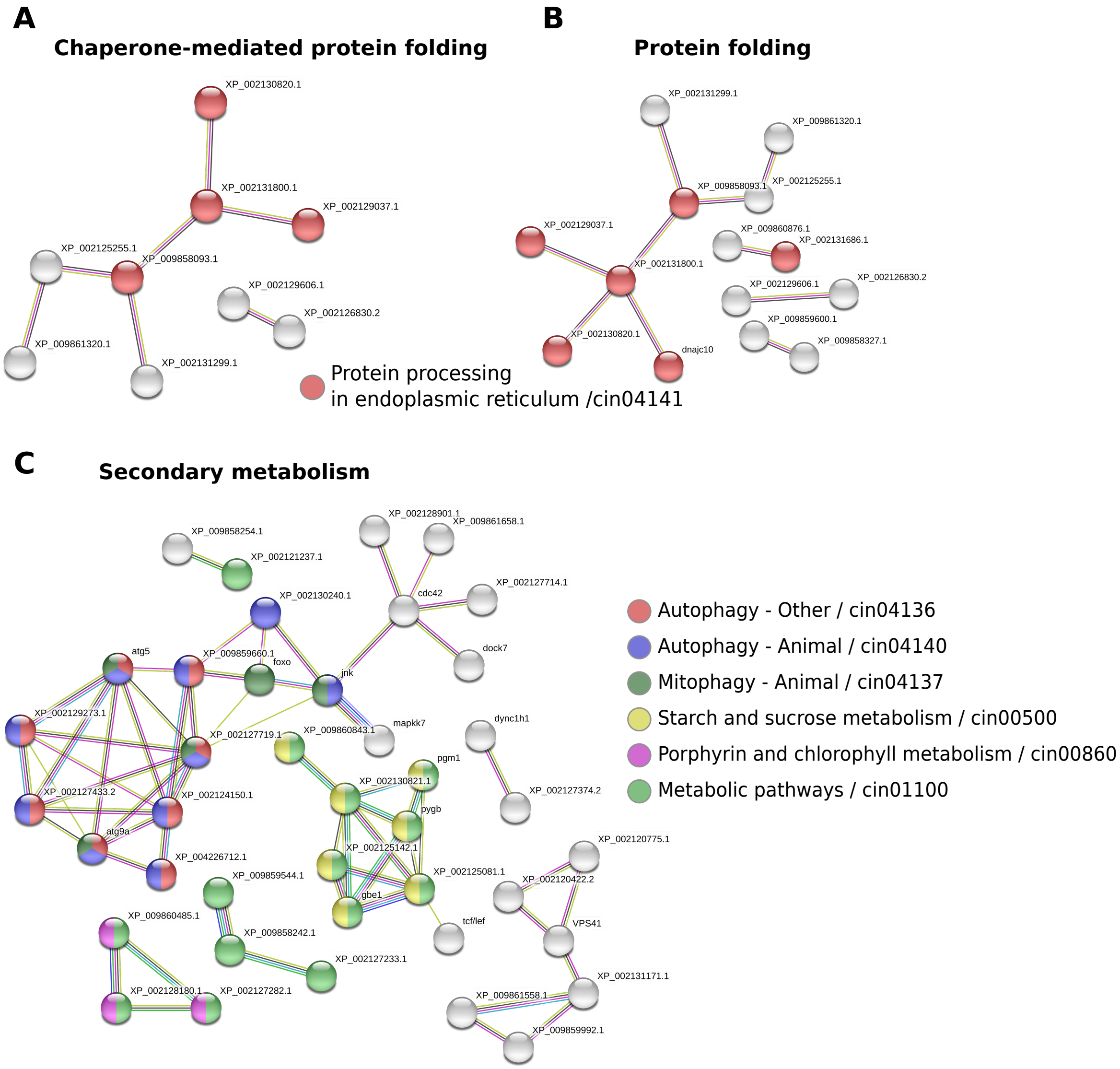

Figure S 27: Functional clusters inferred with `STRING` [?]. Nodes without relations discarded. Cluster labels correspond to obtained clusters depicted on Figure 6, main text. Clusters from categories: *positive regulation of phosphatidylinositol 3-kinase signaling* and *Phosphorus metabolism* were not shown due high number of involved proteins.

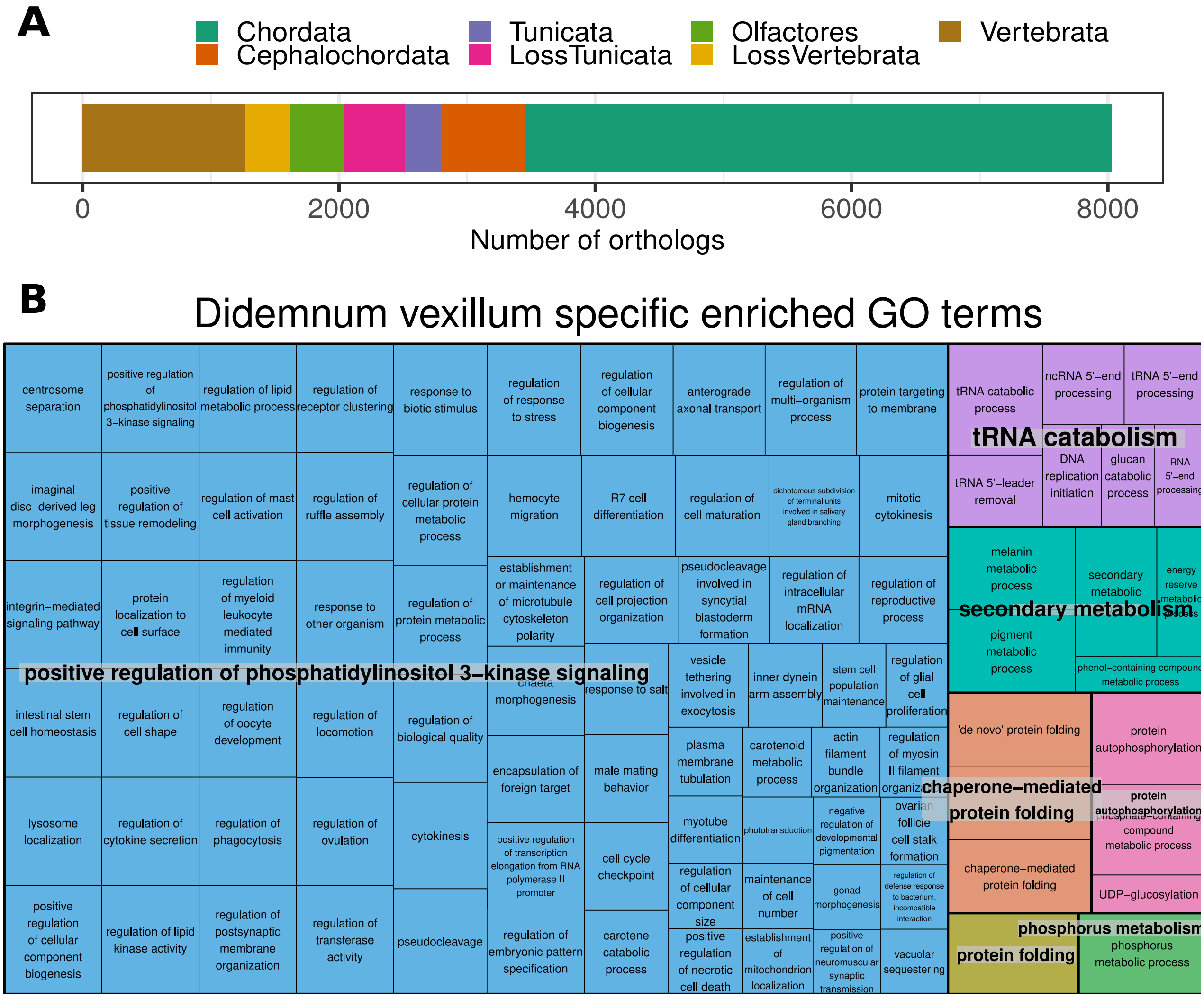

Figure S 28: Comparative genome analyses of *D. vexillum* predicted proteins. **A.** `EggNOG` functional annotation of orthologous groups across chordate genomes. **B.** TreeMap representation from REVIGO [**?**] *D. vexillum* of the enriched GO specific terms. Boxes' area correspond to the adjusted p-value, calculated with `goatools` [**?**].

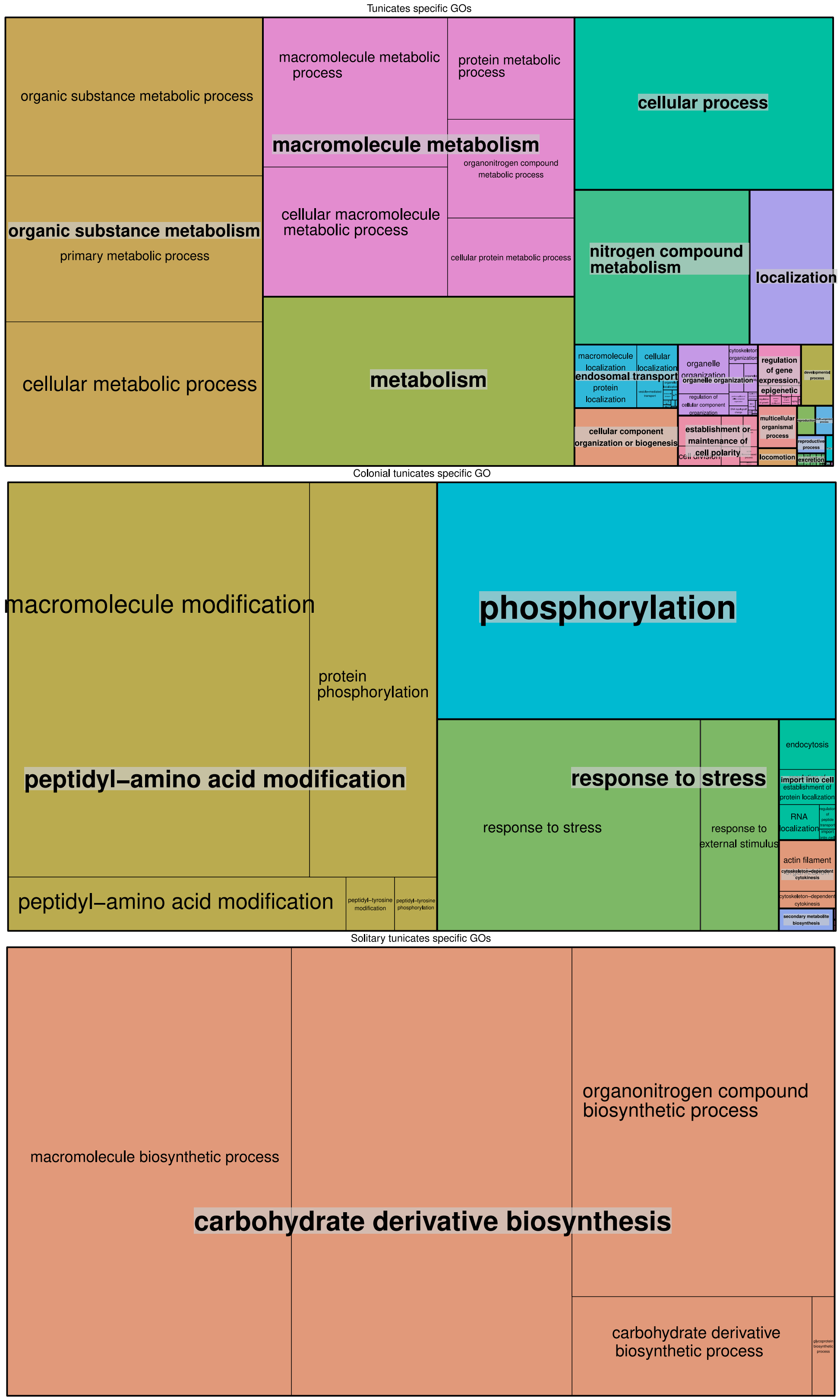

Figure S 29: TreeMap plots of the shared enriched GOs in Tunicata, and more specifically of colonial and solitary tunicates independently. Area of the rectangles is defined based on the frequency of the ontology term over the complete GO list of the studied groups (i.e. Tunicata, colonial Tunicata and solitary Tunicata).

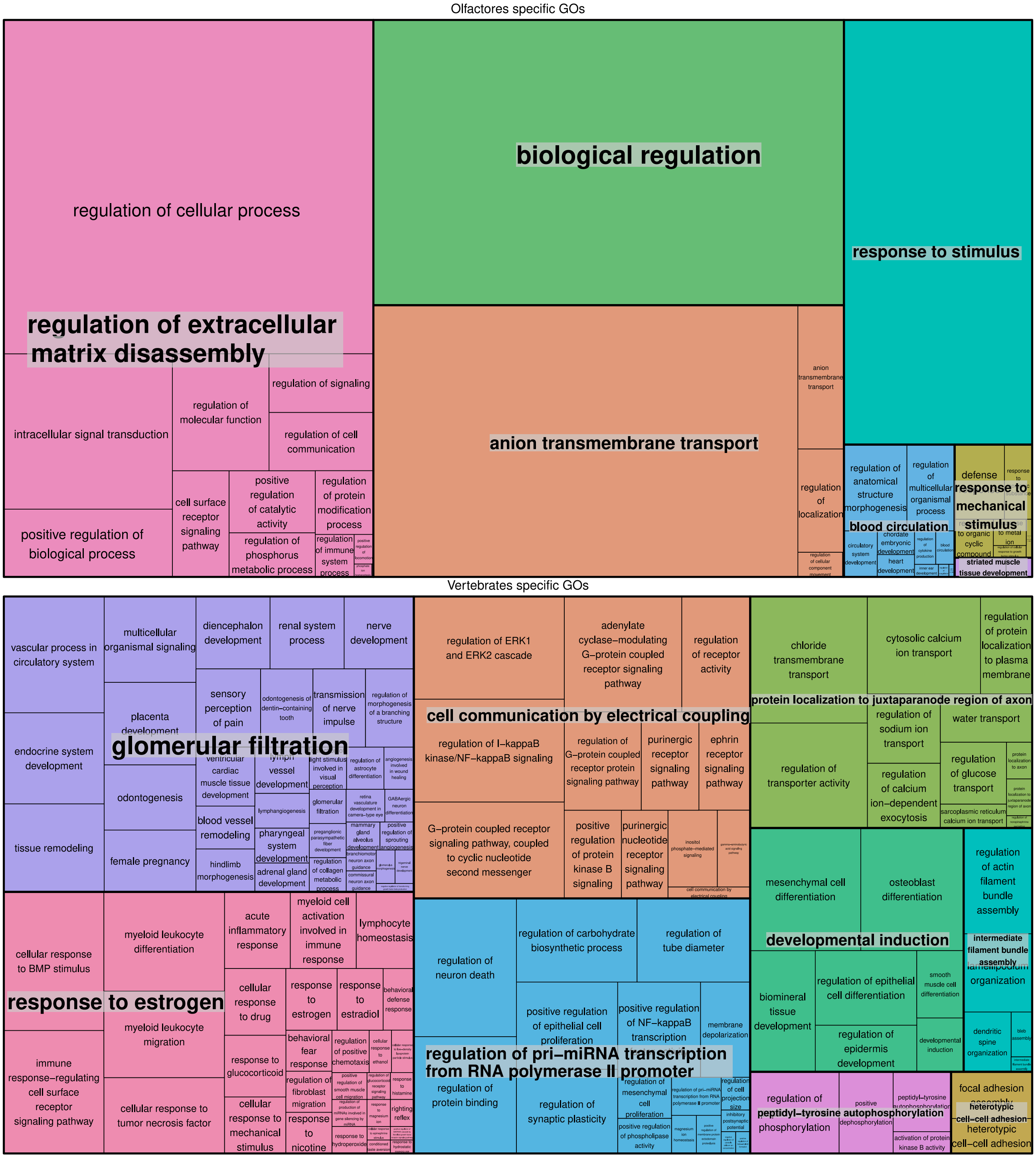

Figure S 30: TreeMap plots of the shared enriched GOs in Olfactores and Vertebrata. Area of the rectangles is defined based on the frequency of the ontology term over the complete GO list of the studied species group (i.e. Olfactores, Vertebrata).

12 Command line Methods

Listings

1 Genome alignments with the close species parameters as suggested by [?]. 35
2 Multiple genome alignments for mtDNA. 35
3 Modification of Covariance Model 35
4 Search CM on genome. 36
5 Orthology relationships. 36
6 GO simplification using GOslims. 36
7 GO enrichment on studied chordate species. 36
8 Protein homology searches with blastp. 36
9 Homology searches with tblastn. 36
10 Search of specific protein domains using hmmscan. 36
11 Structure validation by covariance models. 36
12 Phylogenetic analysis on skeletogenesis proteins using `ETE 3` Toolkit [?]. 37
13 Identification of orthologous proteins and fragmented proteins respect to *C. robusta* proteome. 37
14 ABySS 37
15 DBG2OLC 37
16 Wengan 37
17 wtdbg2 37

Genome Alignments

Genome pairwise alignments were obtained by `lastz` [?]:

```
lastz_32 <GENOME1>[multiple] <GENOME2> --rdotplot=<OUT_DOT-PLOT_FILE> \\
  --ambiguous=iupac --chain C=0 E=30 H=2000 \\
  K=2200 L=6000 M=50 O=400 Q=AlignMatrix/HoxD55.q T=2 Y=3400 \\
  --format=maf+ > <OUTFILE>
```

Listing 1: Genome alignments with the close species parameters as suggested by [?].

Multiple Genome Alignments

```
progressiveMauve --output=<OUT_FILE> --output-guide-tree=<OUT_TREE_FILE>
--backbone-output=<OUT_BACKBONE> <FASTA_SEQ>
progressiveMauve --apply-backbone=<OUT_FILE_GENERATED_LAST_STEP>
--output=<OUT_CLEAN> --backbone-output=<OUT_CLEAN_BACKNONE>
--hmm-p-go-homologous=0.001 --hmm-p-go-unrelated=0.000005
```

Listing 2: Multiple genome alignments for mtDNA.

Modification of Covariance Models

Rebuilding the new covariance model was possible using:

```
clustalo -i <multifasta file> --outfmt clu -o <output file>
RNAalifold --aln-stk=<fasta file> <align file>
cmbuild <Covariance Model> <STO file>
cmcalibrate --cpu=20 <CM>
```

Listing 3: Modification of Covariance Model

Then, generated CM was searched directly with cmsearch:

```
cmsearch --cpu 4 --tblout <TABULAR OUT> -o <OUT> <CM> <GENOME>
```

Listing 4: Search CM on genome.

Functional Annotation and Protein Enrichment

For the sequence searches, with the following parameters:

```
emapper.py --cpu 10 -i <FASTA FILE> --output <OUTFILE> --output_dir <OUT DIR>
-m diamond -d none --tax_scope auto --go_evidence non-electronic
--target_orthologs one2one --seed_ortholog_evalue 0.001 --seed_ortholog_score
60 --query-cover 20 --subject-cover 0 --override --temp_dir <TEMP DIR>
```

Listing 5: Orthology relationships.

Output file was processed with a Perl scripts to generate input files to obtain clusters of orthologs, based on a common orthology seed. At the same time, GO annotation was transferred from query seed to subject proteins. Based on this relationship, required files to calculate GO enrichment was obtained.

In order to show an overview of GO terms inside studied genomes, obtained GO terms were simplified using the generic GOslim (goslim_generic.obo, downloaded on 10.06.2019[7]) database with `goatools` [**?**] as follows:

```
map_to_slim.py --slim_out=all --association_file=<Association> go-basic.obo
goslim_generic.obo
```

Listing 6: GO simplification using GOslims.

Enrichment analysis using Fisher's exact test with Benjamini-Hochberg multiple test correction on p-values was calculated using `goatools` [**?**] based on protein counts, as follows:

```
find_enrichment.py --pval=0.05 --alpha=0.05 --no_propagate_counts
--method=fdr_bh --pval_field=fdr_bh --indent --outfile=<OUTFILE> <Subset>
<Population> <Association Mapped GOslim>
```

Listing 7: GO enrichment on studied chordate species.

In the designed experiment, `Subset` file is the collection of proteins on a specific specie. The background file, (`Population`) was conformed by all the proteins that belong from previously selected chordate species, including tunicates. The `Association` file is the reduced set, calculated with `map_to_slim.py`, between proteins and their assigned set of GO obtained with `eggNOG-Mapper`.

Homology searches

```
blastall -p blastp -d <PROTEOME> -i <QUERY> -W 2 -f 9 -F "m S" -M BLOSUM45 -e
100 -b 10000 -v 10000 -m 8
```

Listing 8: Protein homology searches with blastp.

```
blastall -p tblastn -d <TRANSCRIPTS> -i <QUERY> -f 999 -e 1e-5 -m 8 -o <OUT>
```

Listing 9: Homology searches with tblastn.

```
hmmscan -o <OUT_FILE> --tblout <OUT_FILE_TABULAR> --domtblout
<OUT_FILE_SPECIFIC> <HMM> <SEQ>
```

Listing 10: Search of specific protein domains using hmmscan.

```
cmsearch -g -Z <NT number (Mb)> --toponly --tblout <OUT_TABULAR> -o <OUT_FILE> <FASTA> <
    CM>
```

Listing 11: Structure validation by covariance models.

[7] `http://current.geneontology.org/ontology/subsets/goslim_generic.obo`

Phylogenetic analysis

```
ete3 build -a <target_seq> --cpu 16 -o <project_name> -w
clustalo_default-trimal01-prottest_default-phyml_default_bootstrap --noimg
```

Listing 12: Phylogenetic analysis on skeletogenesis proteins using `ETE 3` Toolkit [?].

D. vexillum proteome comparisons

```
proteinortho6.pl <Proteins_D_vexillum> <Proteins_C_robusta> -cpus=16 -project=<
    Project_Name> -p=blastp -e=1e-03
```

Listing 13: Identification of orthologous proteins and fragmented proteins respect to *C. robusta* proteome.

Alternative Assembly Strategies

```
abyss-pe name=<Output> k=96 B=40G H=4 kc=3 v=-v  np=40 lib="pe1" pe1="<Illumina
    Sequences 1 2>" <Output Folder>
```

Listing 14: ABySS

```
SparseAssembler LD 0 k 51 g 15 NodeCovTh 1 EdgeCovTh 0 GS 140000000 f <Illumina
    Sequences 1> f <Illumina Sequences 2>
DBG2OLC k 17 AdaptiveTh 0.0001 KmerCovTh 2 MinOverlap 20 RemoveChimera 1 Contigs ../
    Contigs.txt f <PacBio Sequences>
seqtk seq -a <PacBio Sequences> > ctg_pb.fasta
split_and_run_sparc.sh  backbone_raw.fasta DBG2OLC_Consensus_info.txt ctg_pb.fasta ./
    consensus_dir 2 >cns_log.txt
```

Listing 15: DBG2OLC

```
wengan.pl -x pacraw -a A -s  <Illumina Sequences 1, 2> -l <PacBio Sequences> -p asm2 -t
    40 -g 1500
```

Listing 16: Wengan

```
wtdbg2.pl -t 30 -x rs -g 1.5g -o wtdbg2 <PacBio Sequences>
```

Listing 17: wtdbg2

LazyB was run at standard parameters but with previous all-vs-all scrubbing of Oxford Nanopore reads via the script provided by the prototype implementation. We tested several custom modifications of our pipeline but found no worthwhile improvements and results are accordingly not reported here.
