# Supplementary figures and images for: The Genome of the “Sea Vomit” Didemnum vexillum
Source: Life (Basel). 2021 Dec 10;11(12):1377. doi: 10.3390/life11121377 (PMC8704543; doi:10.3390/life11121377)

# BUSCO Assessment Results

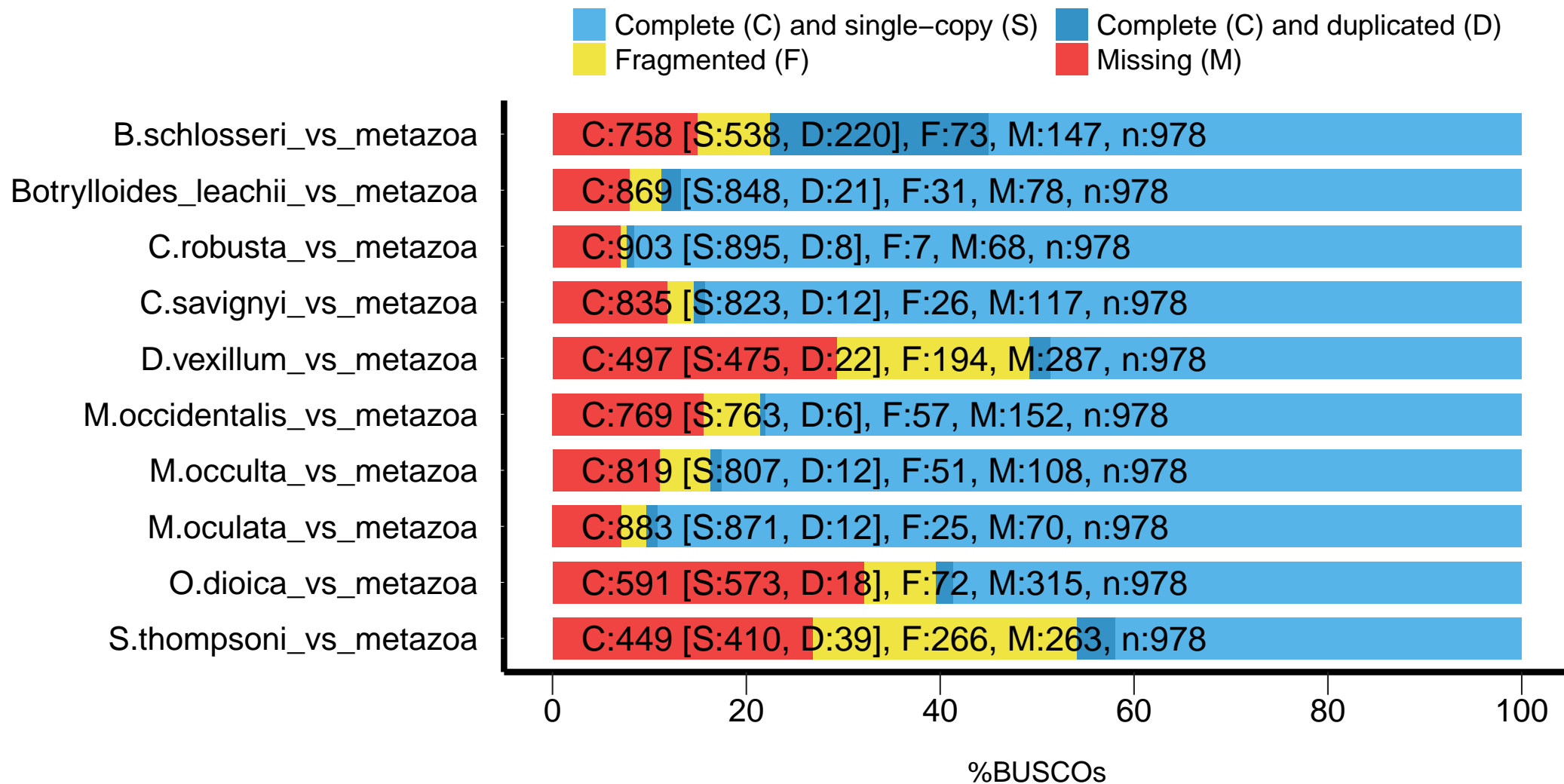

Supplement: Supplementary file 1 [file life-11-01377-s001.zip › Figures/busco_figure.pdf]

**A**

# Chaperone-mediated protein folding

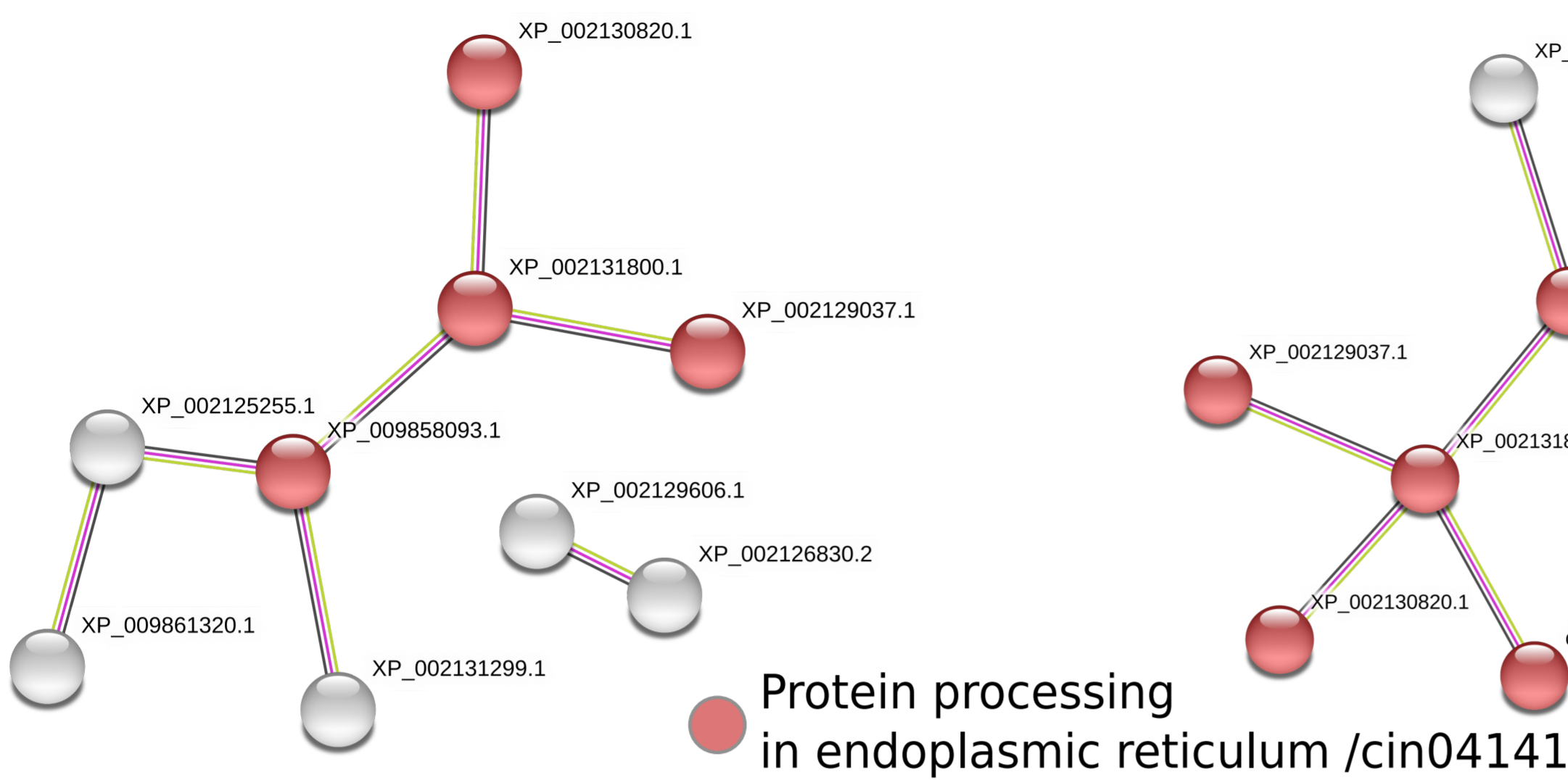**B**

# Protein folding

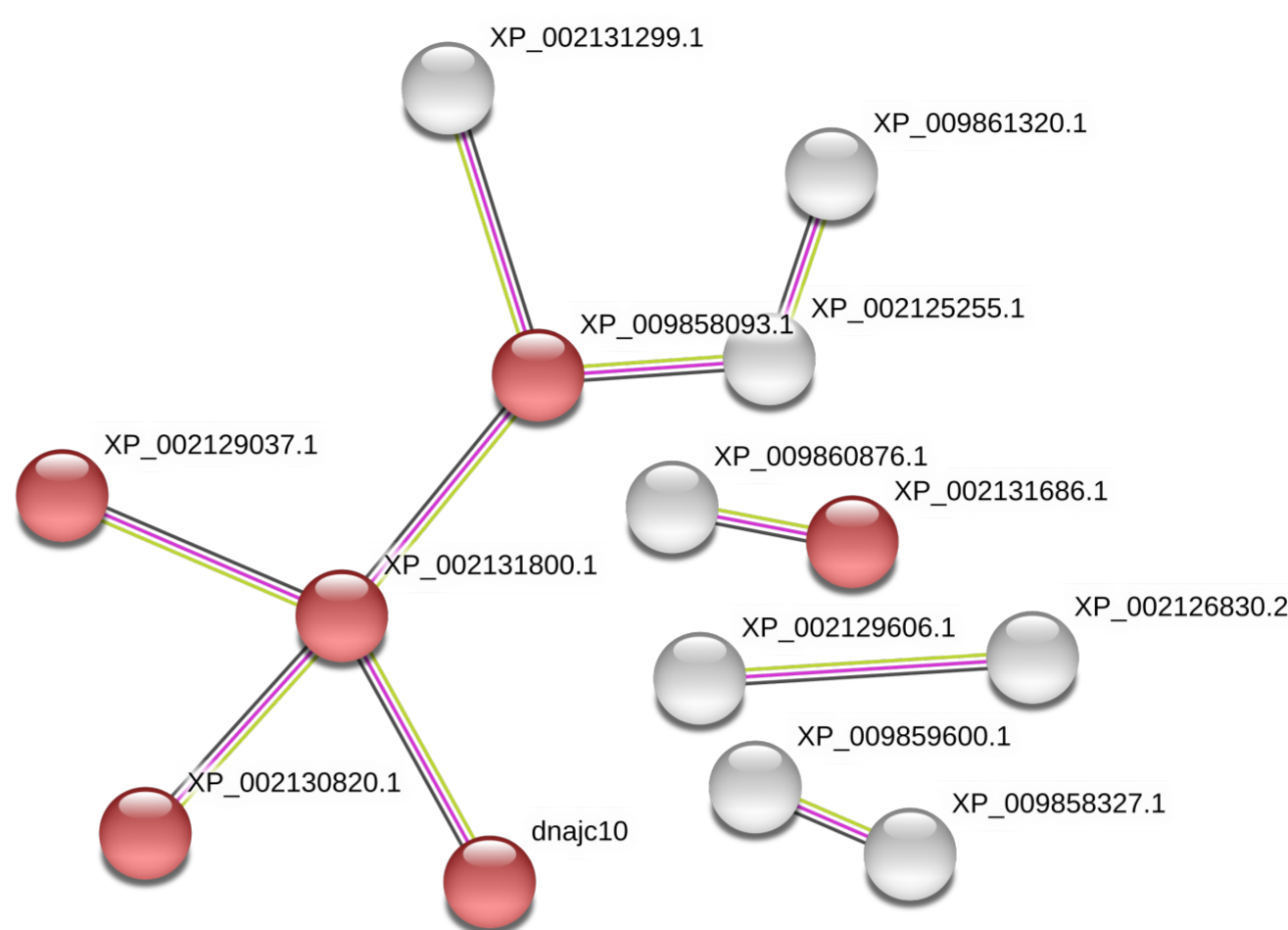**C**

# Secondary metabolism

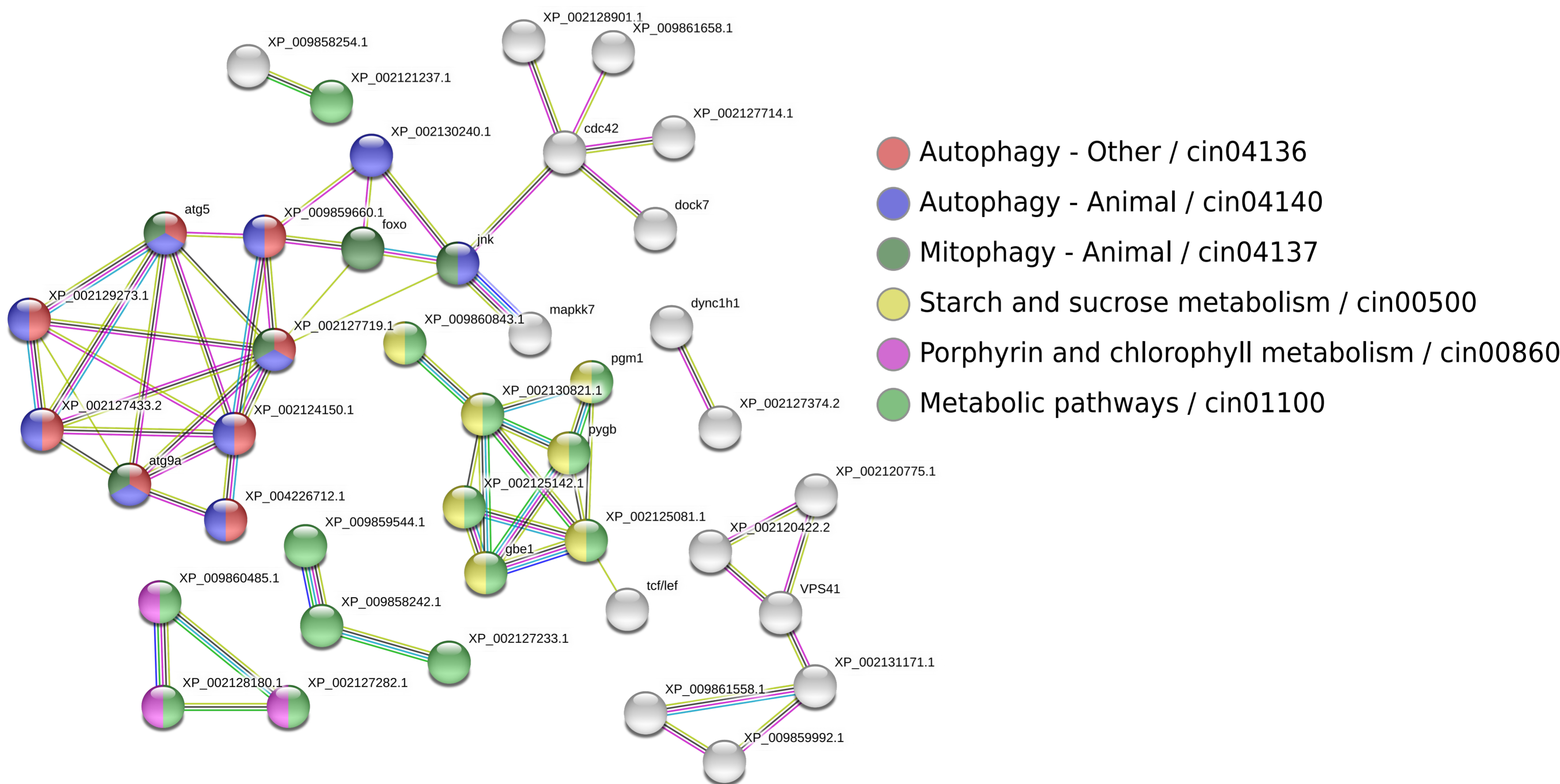

Supplement: Supplementary file 1 [file life-11-01377-s001.zip › Figures/clusters-GO-supplemental.pdf]

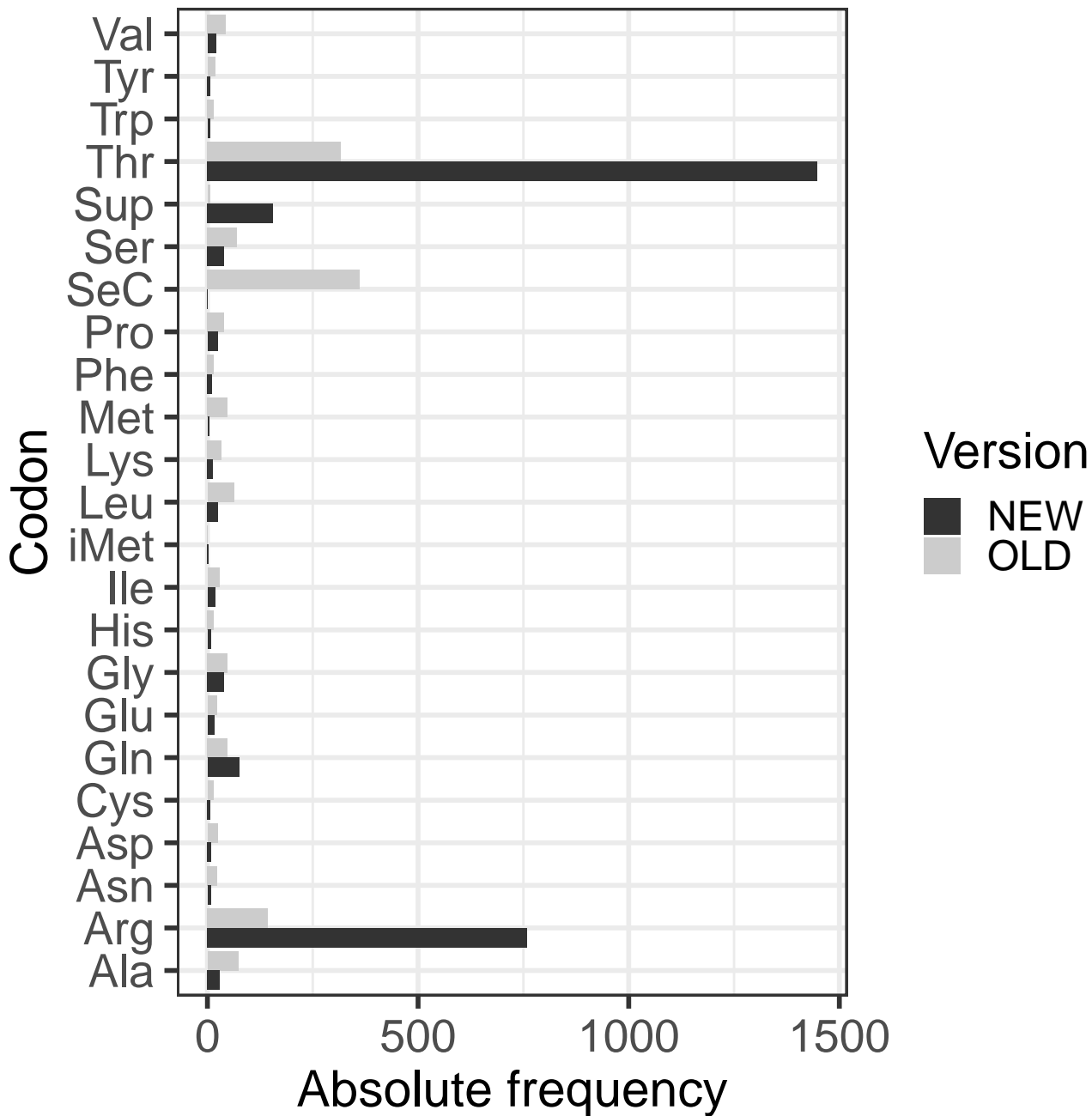

Supplement: Supplementary file 1 [file life-11-01377-s001.zip › Figures/comparison_freq_tRNAs.pdf]

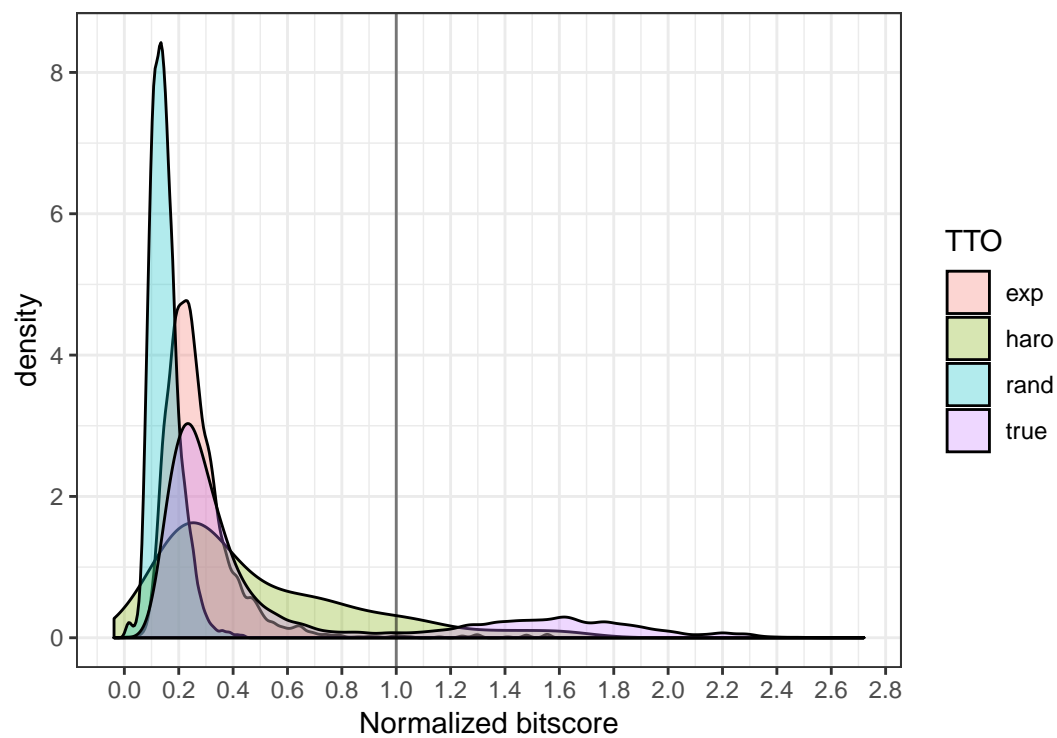

Supplement: Supplementary file 1 [file life-11-01377-s001.zip › Figures/complete_distribution_nbits.pdf]

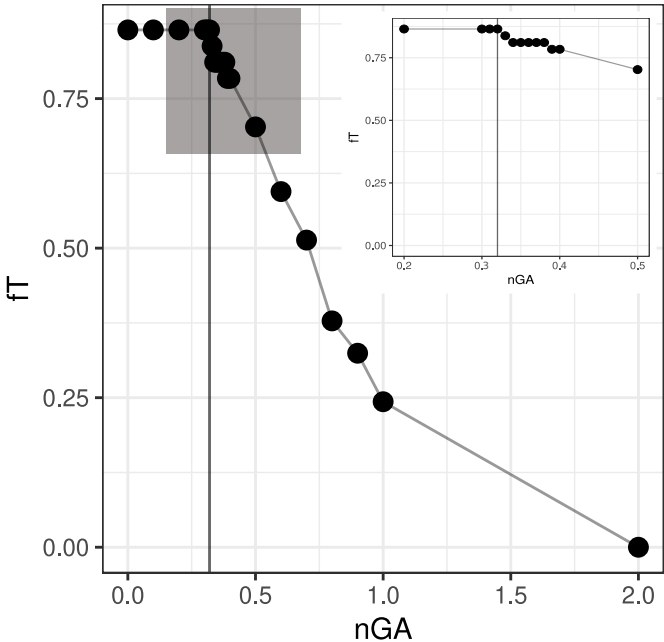

Supplement: Supplementary file 1 [file life-11-01377-s001.zip › Figures/complete_haro_nBitcore.pdf]

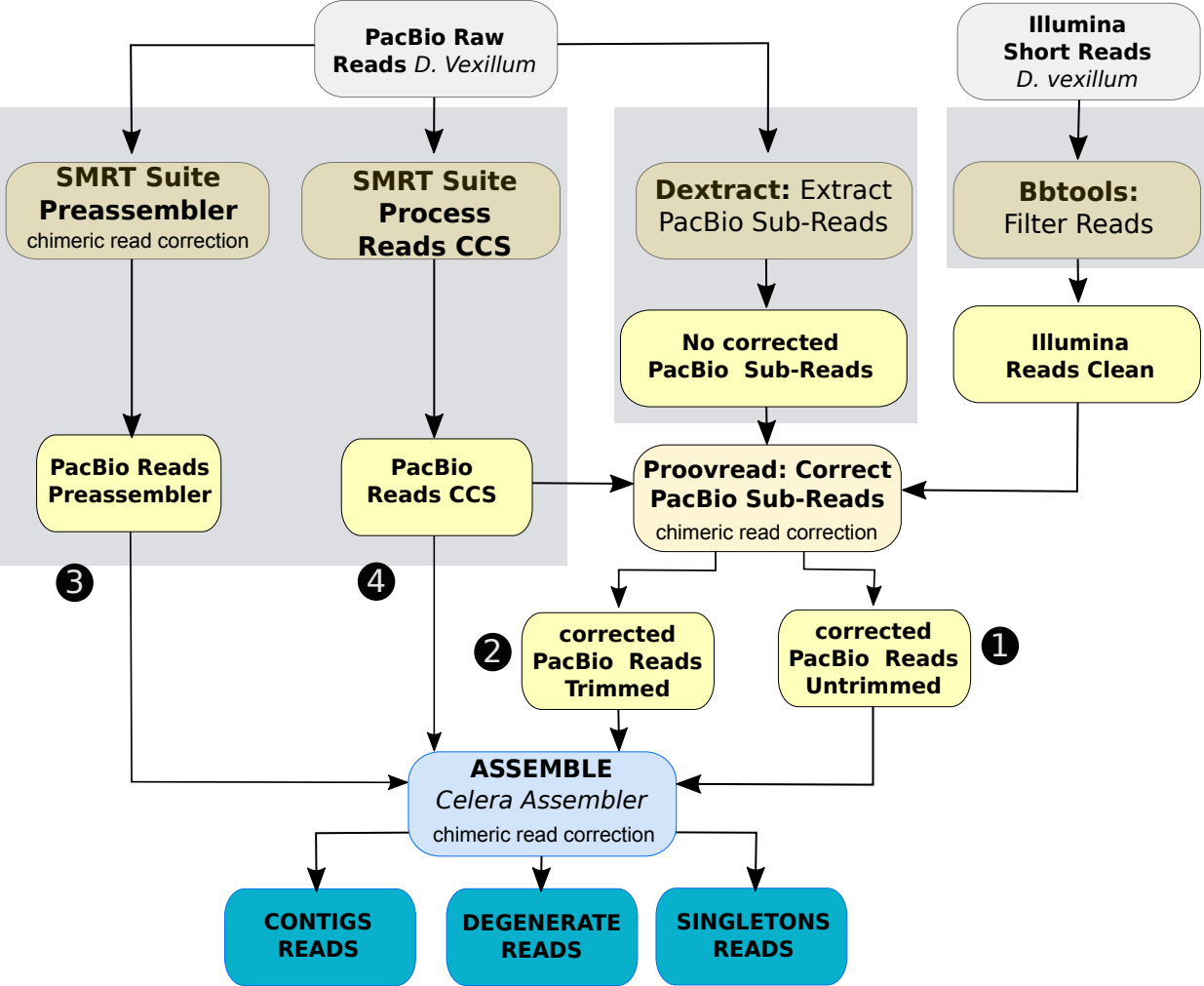

Supplement: Supplementary file 1 [file life-11-01377-s001.zip › Figures/Correct_Pacbio.pdf]

**C**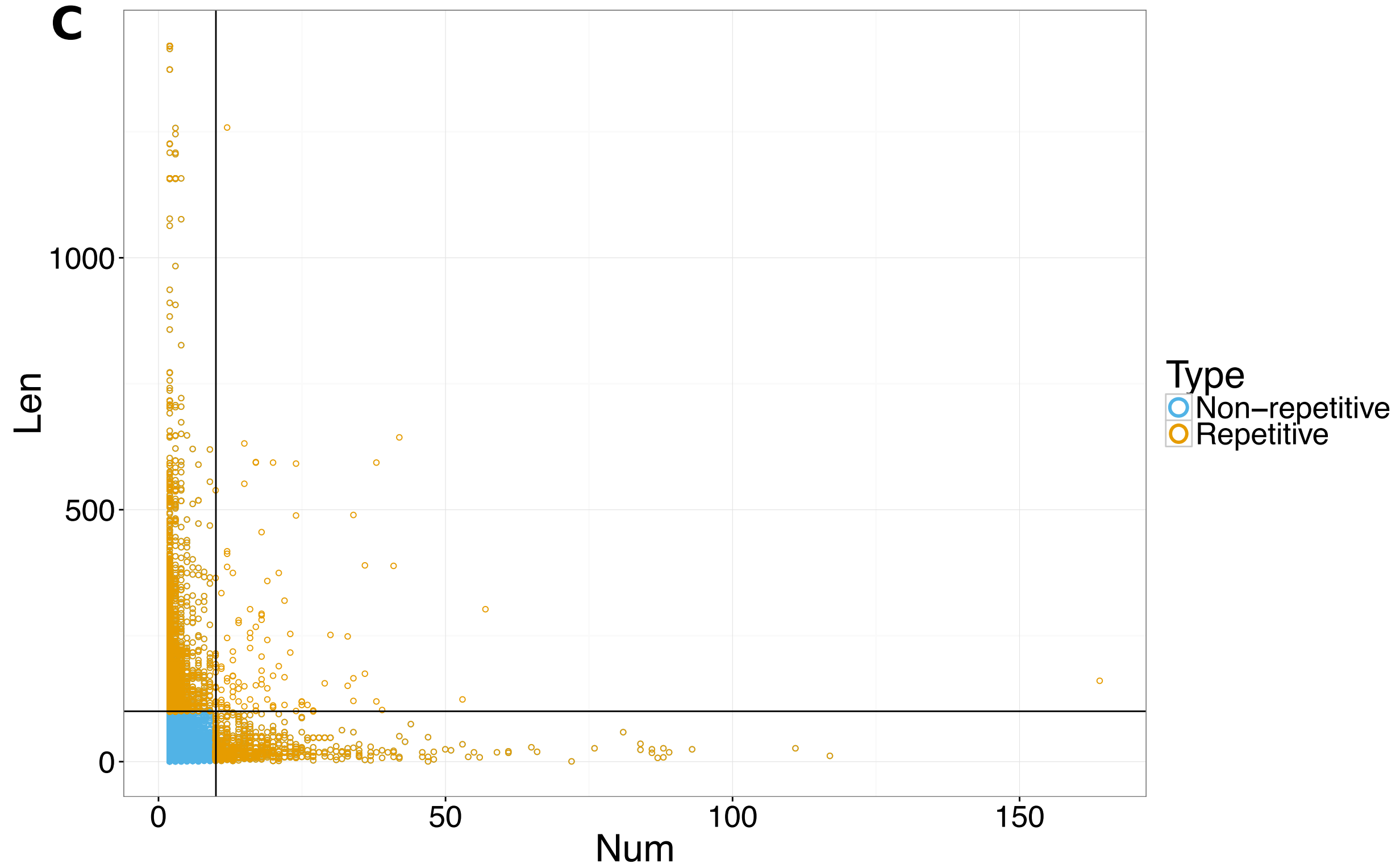

Supplement: Supplementary file 1 [file life-11-01377-s001.zip › Figures/density-threshold-value.pdf]

**B**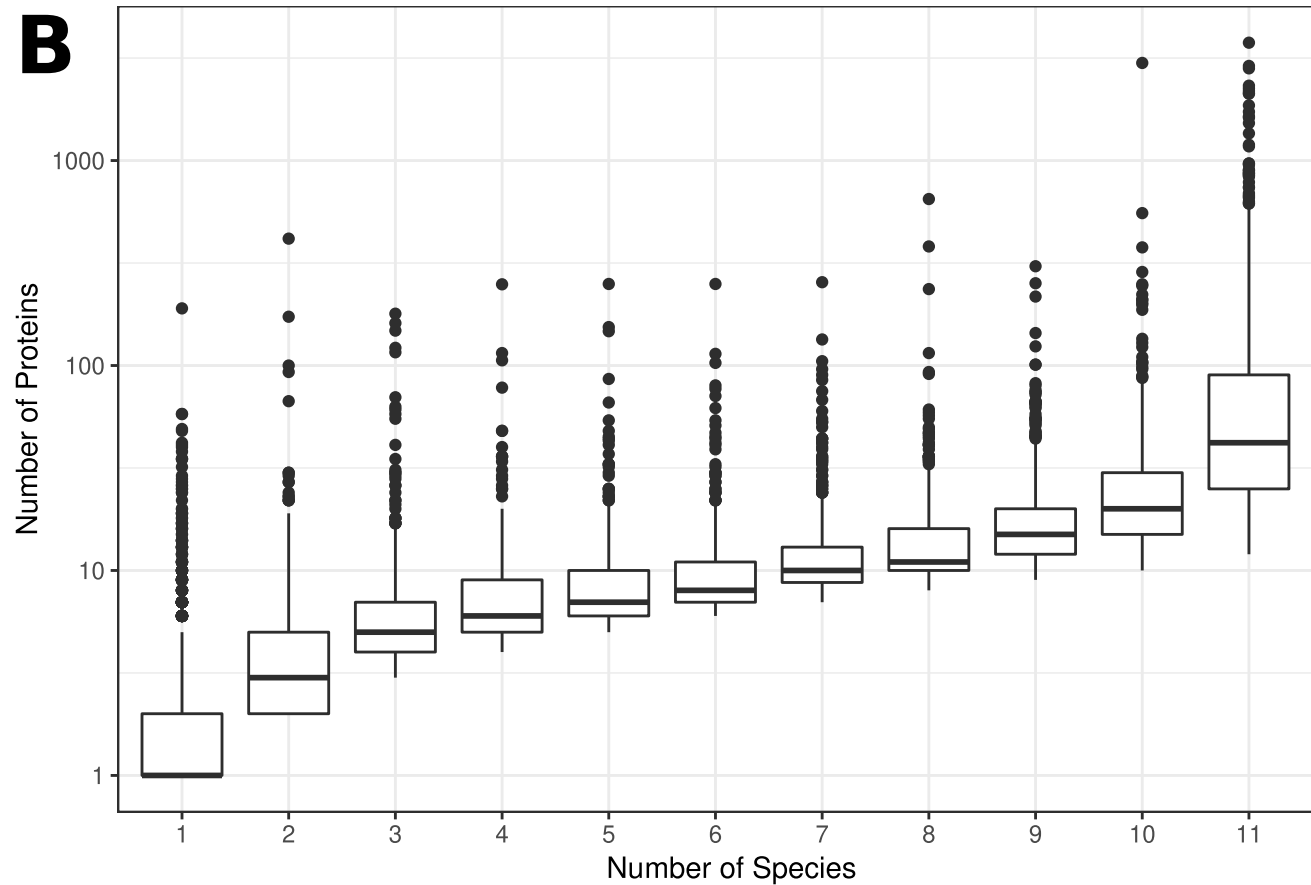

Supplement: Supplementary file 1 [file life-11-01377-s001.zip › Figures/distribution-proteins-clade-final.pdf]

**A**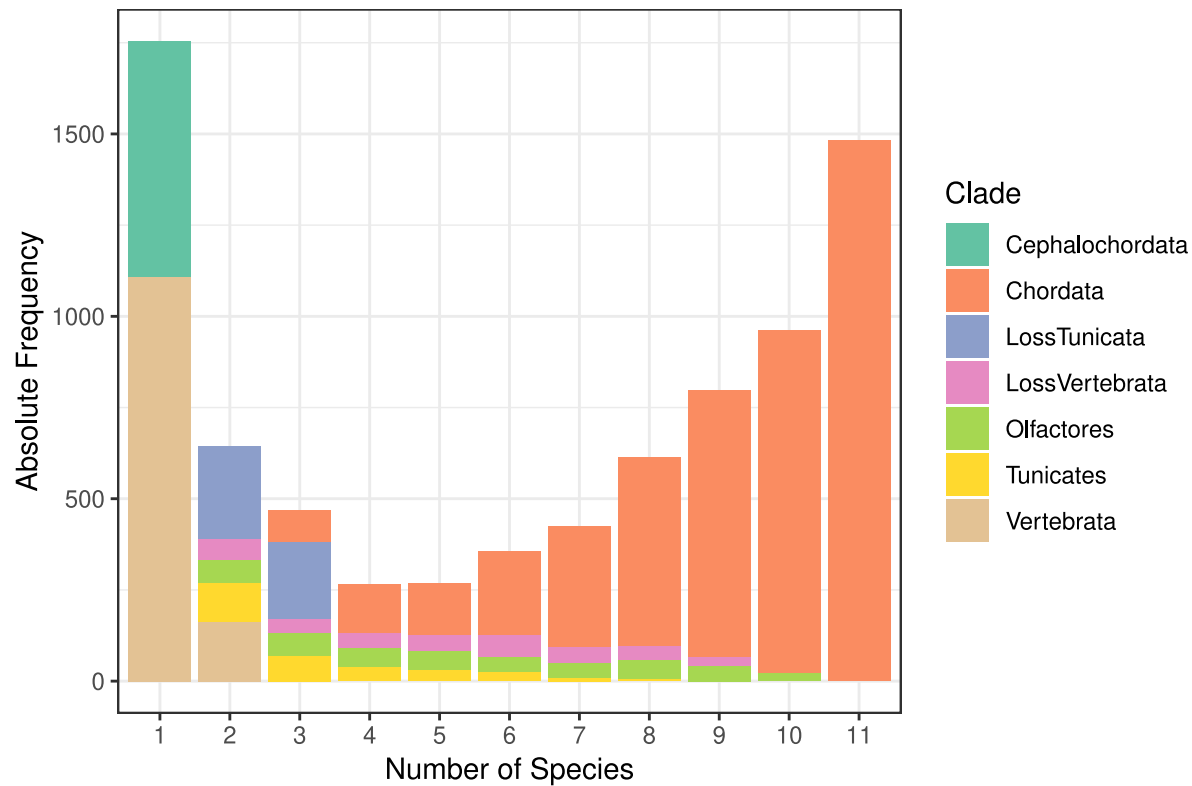

Supplement: Supplementary file 1 [file life-11-01377-s001.zip › Figures/distribution-species-clade-final.pdf]

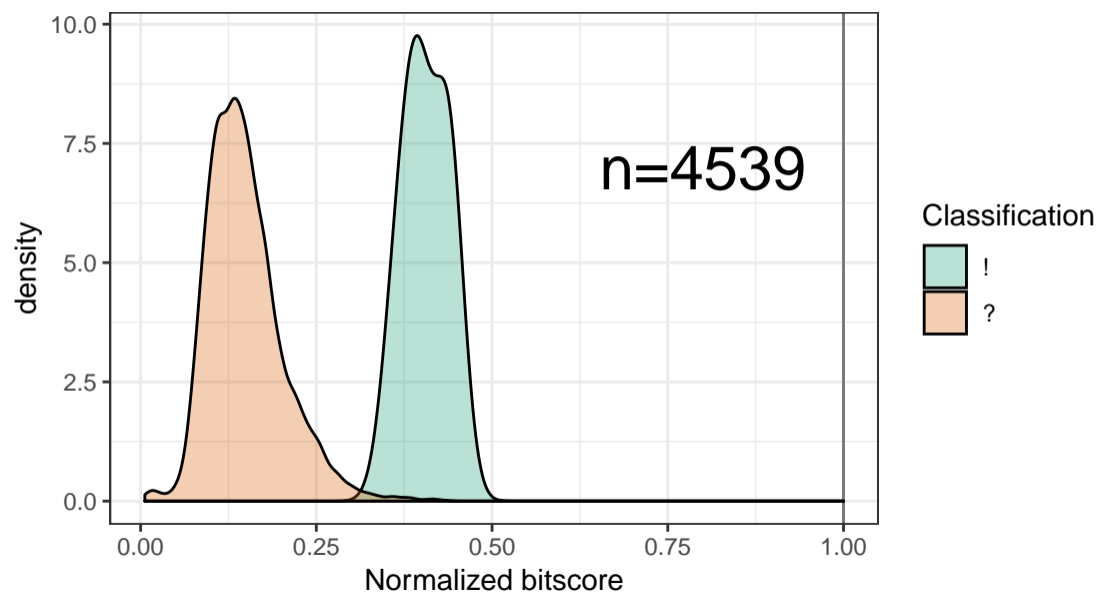

Supplement: Supplementary file 1 [file life-11-01377-s001.zip › Figures/distributions_negative.pdf]

**A**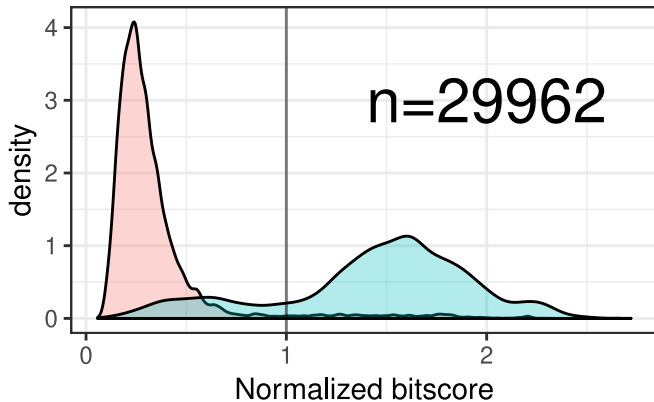**B**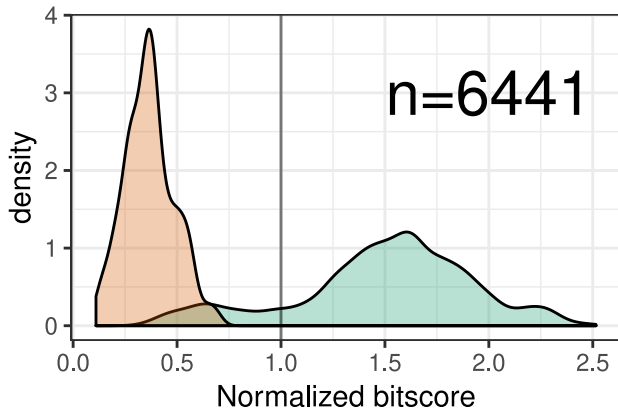

Supplement: Supplementary file 1 [file life-11-01377-s001.zip › Figures/distributions_true.pdf]

**B**

Absolute value

1000

10

Length

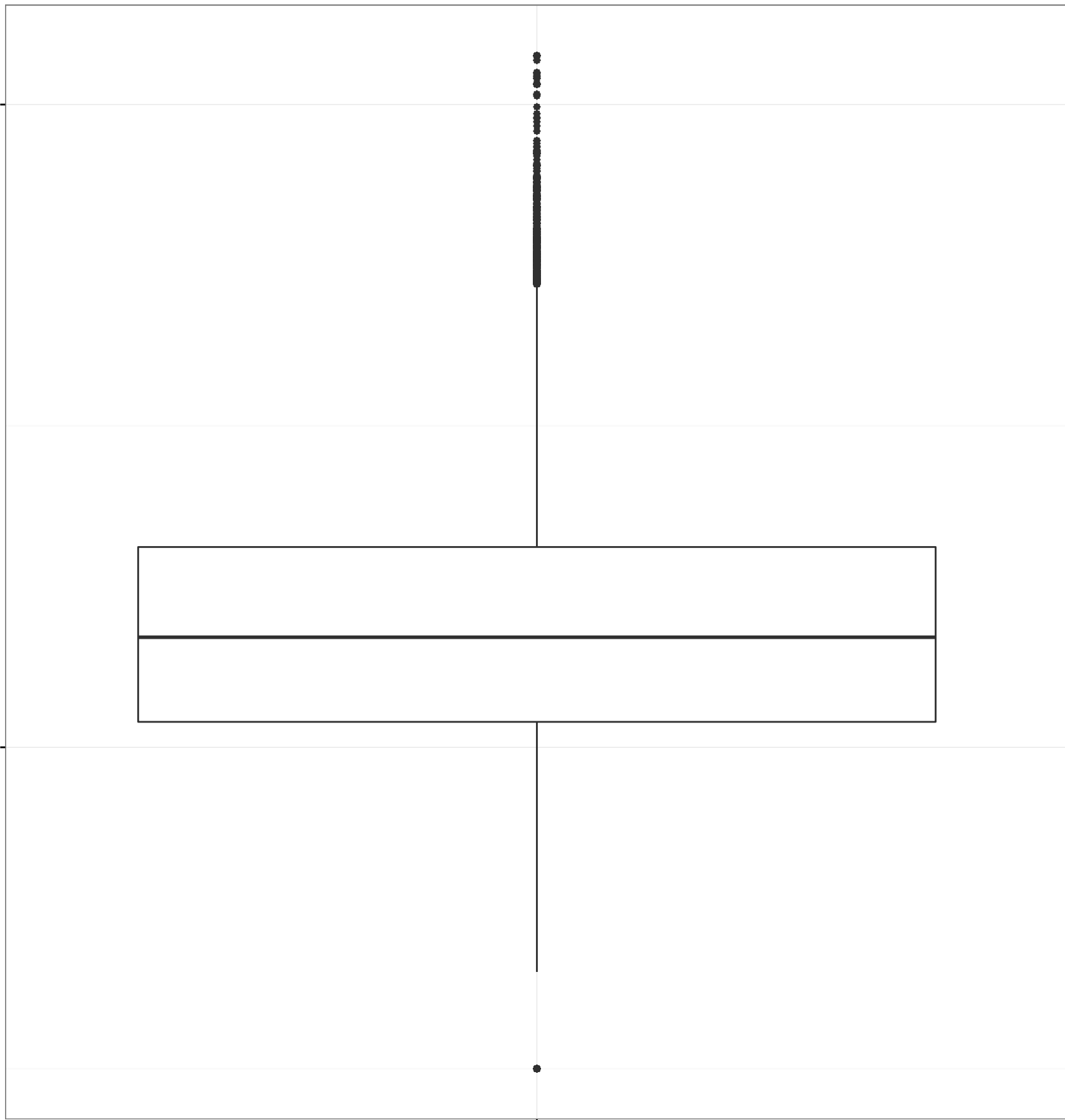

Supplement: Supplementary file 1 [file life-11-01377-s001.zip › Figures/distribution_length_contigs.pdf]

**A**

Absolute value

100

10

Repeated blocks

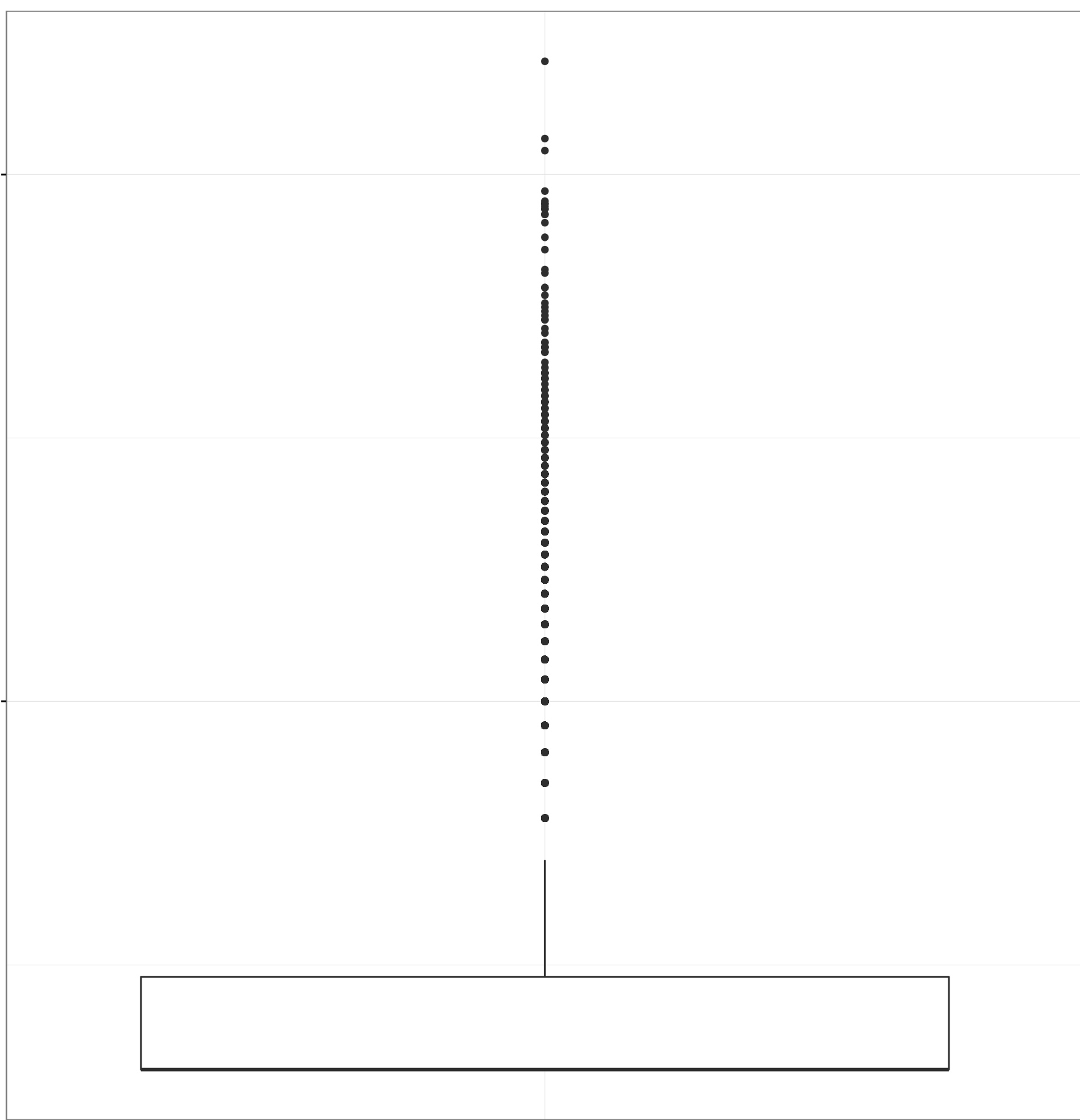

Supplement: Supplementary file 1 [file life-11-01377-s001.zip › Figures/distribution_repetitions_contigs.pdf]

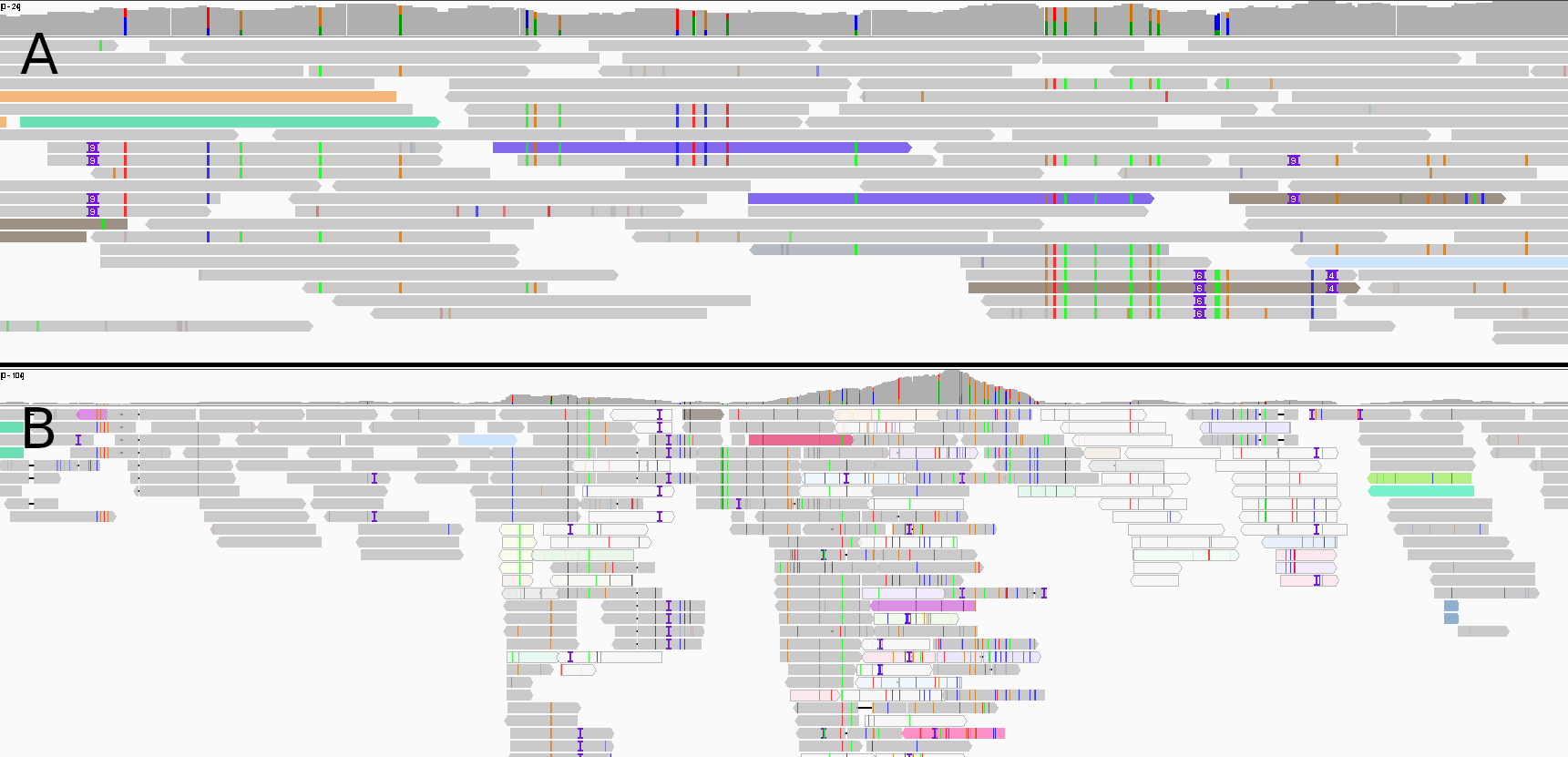

Supplement: Supplementary file 1 [file life-11-01377-s001.zip › Figures/dvex_alignment_example.png]

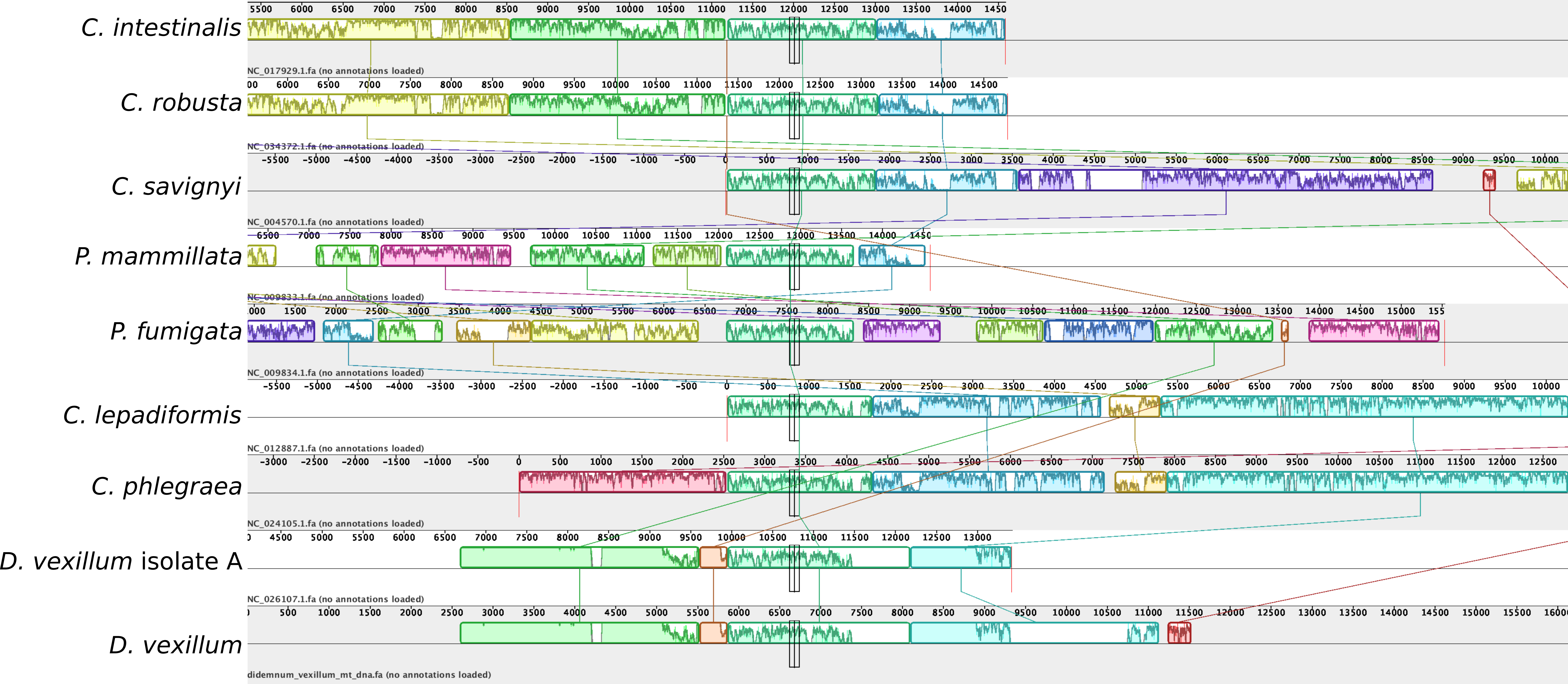

Supplement: Supplementary file 1 [file life-11-01377-s001.zip › Figures/finalAlignMTF.pdf]

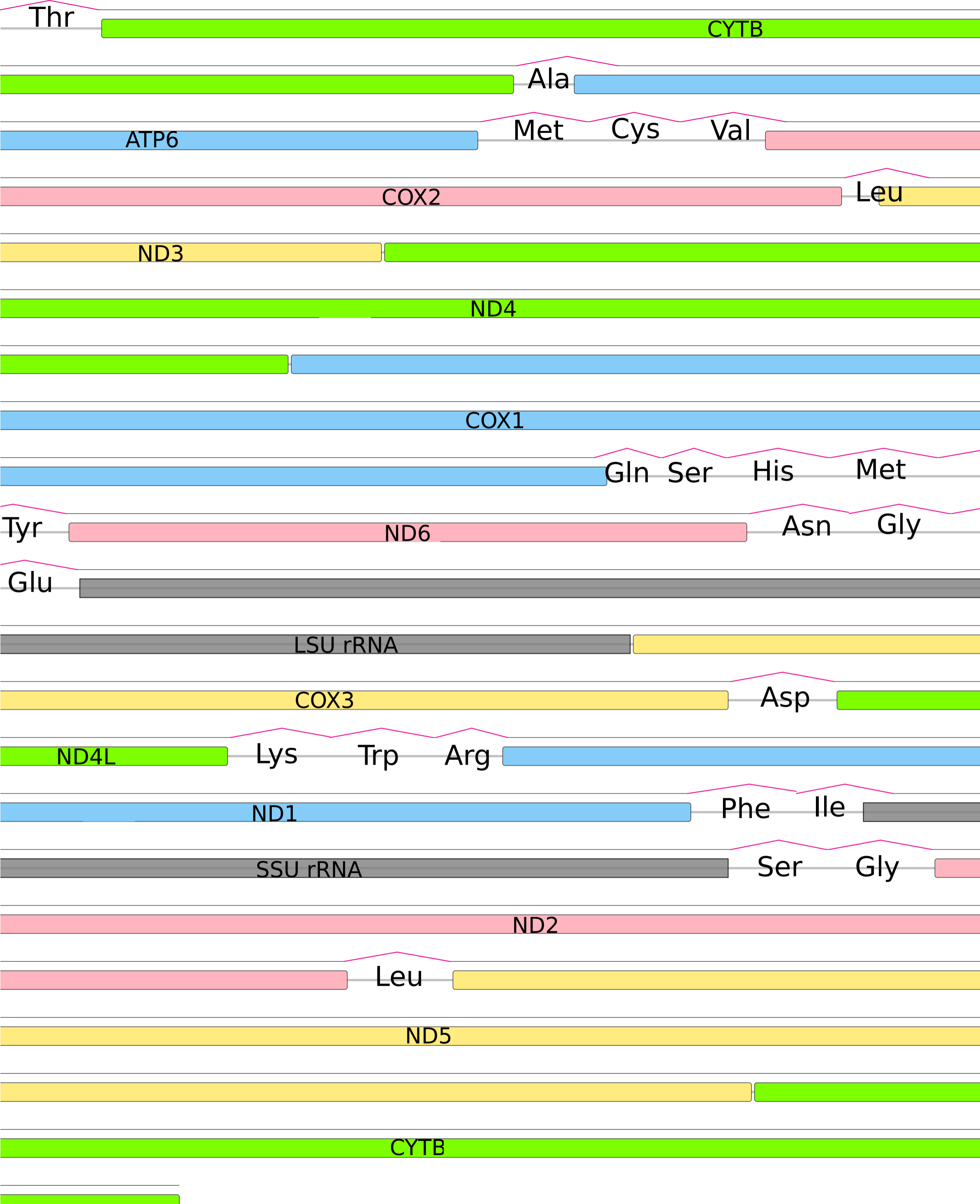

Supplement: Supplementary file 1 [file life-11-01377-s001.zip › Figures/finalMito.pdf]

scaffold1656-size16126

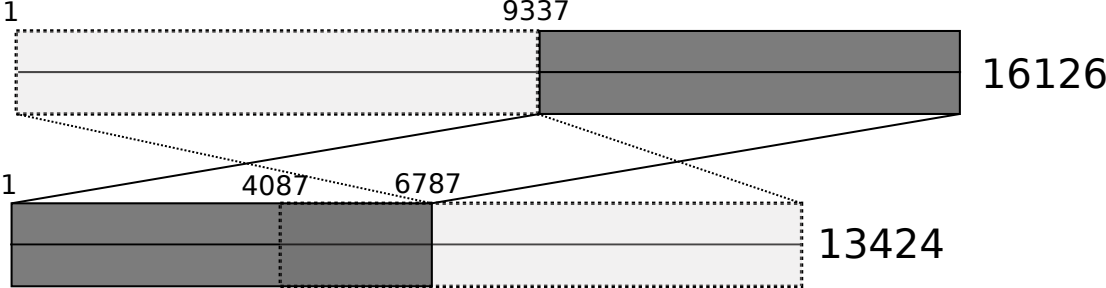

Supplement: Supplementary file 1 [file life-11-01377-s001.zip › Figures/genomeMtAlignmentFinalDvex.pdf]

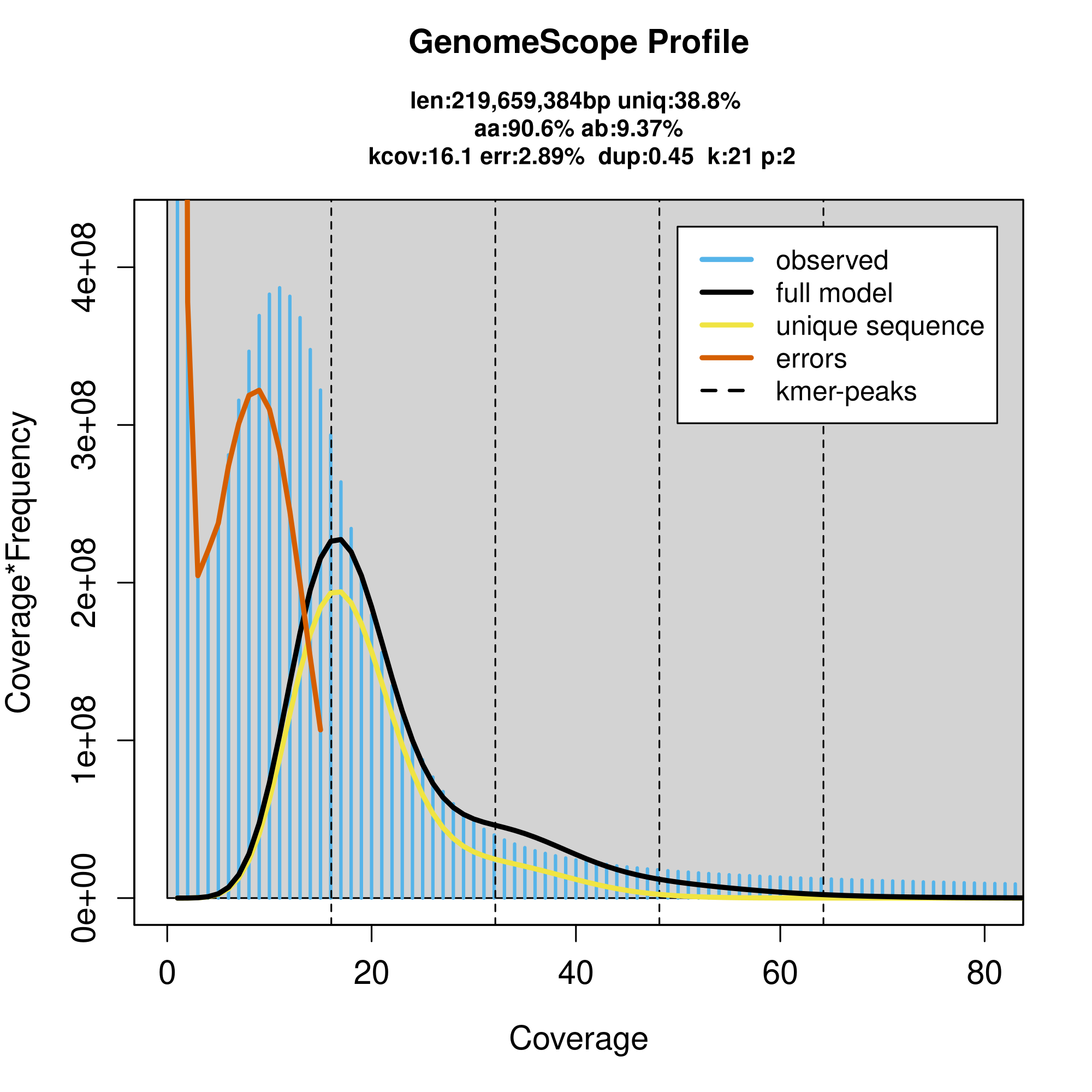

Supplement: Supplementary file 1 [file life-11-01377-s001.zip › Figures/genomescope_profile.png]

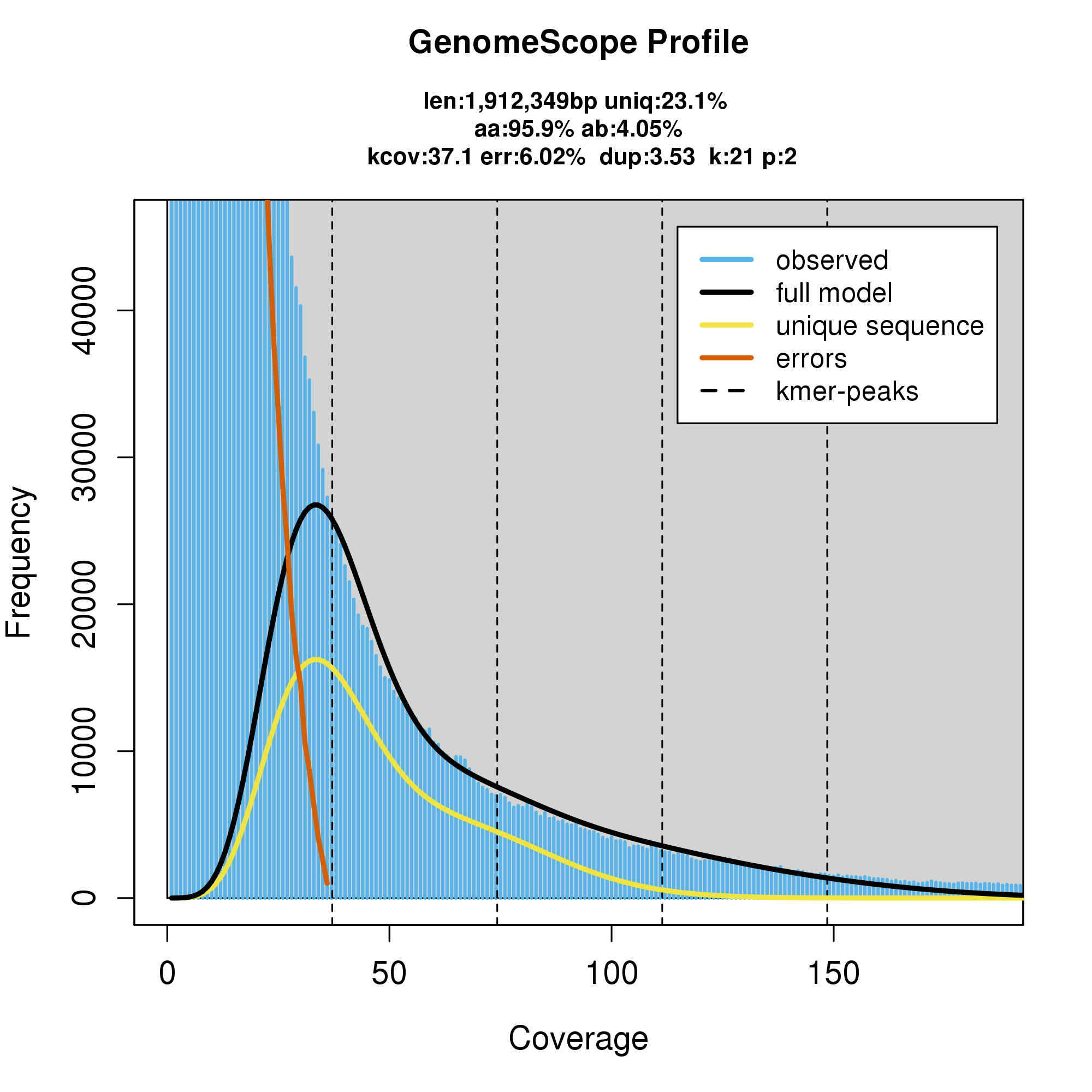

Supplement: Supplementary file 1 [file life-11-01377-s001.zip › Figures/genomescope_profile_old.png]

**A**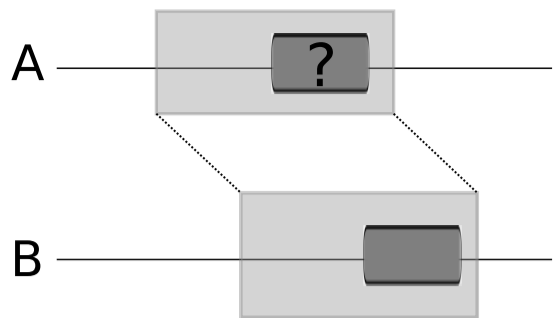**B**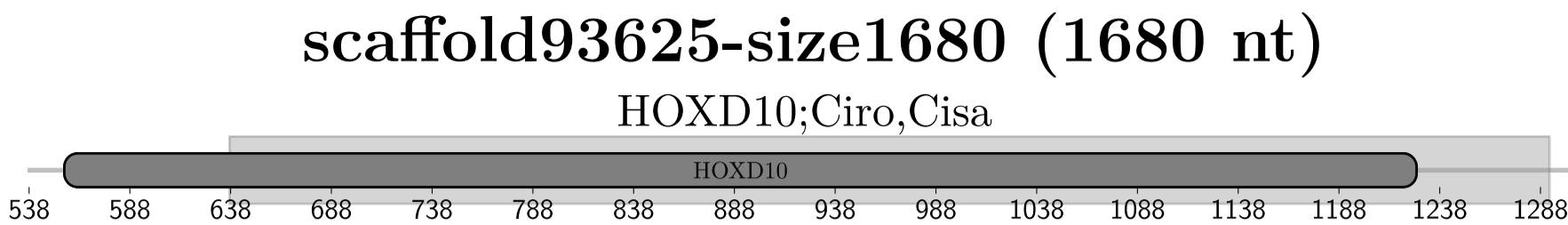**C**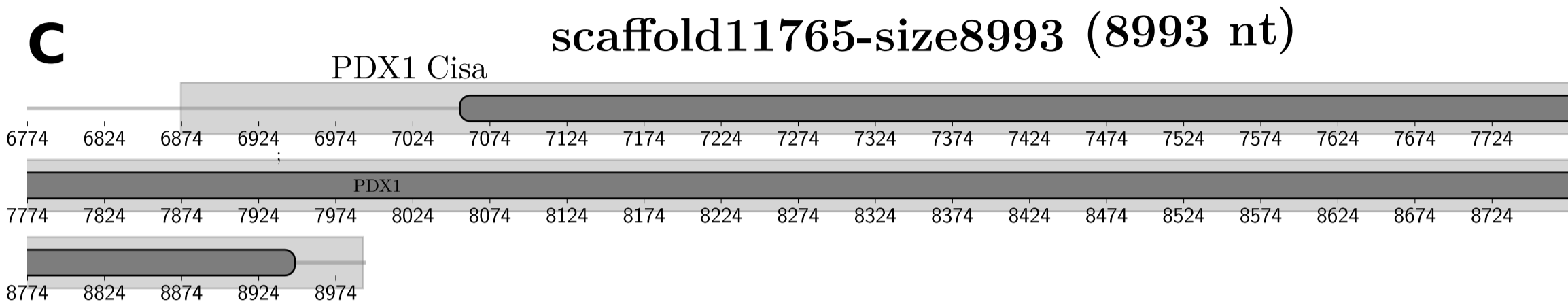**D**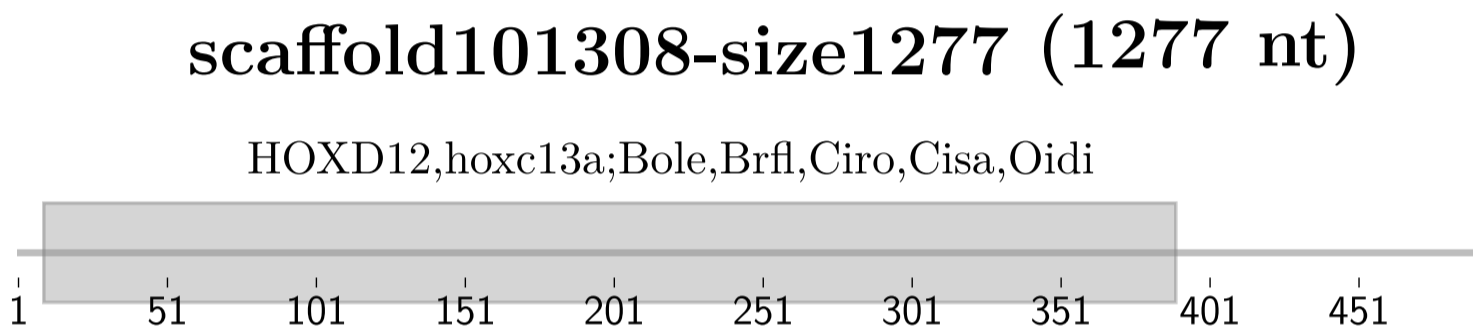**E**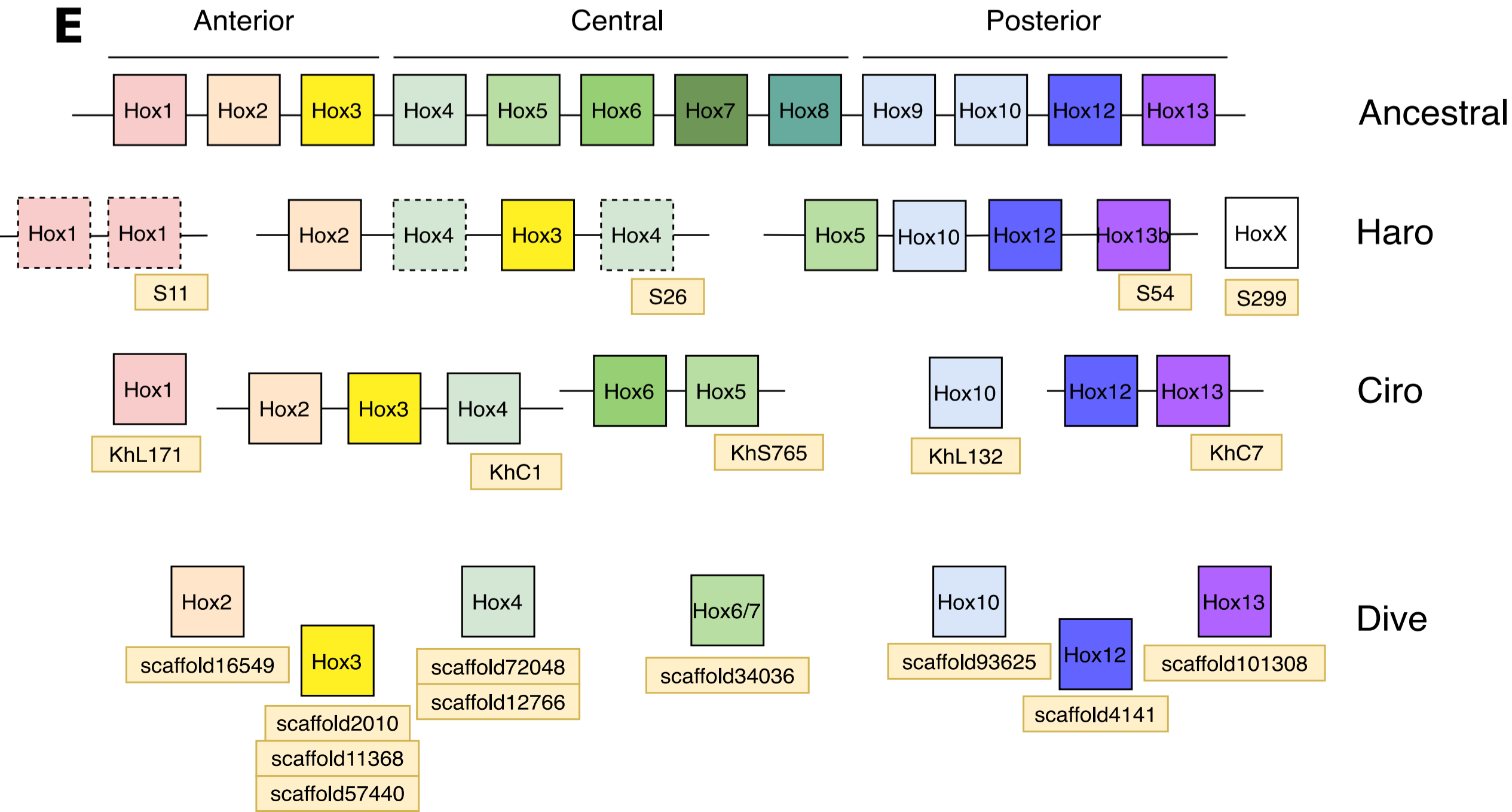

Supplement: Supplementary file 1 [file life-11-01377-s001.zip › Figures/homeobox-complete.pdf]

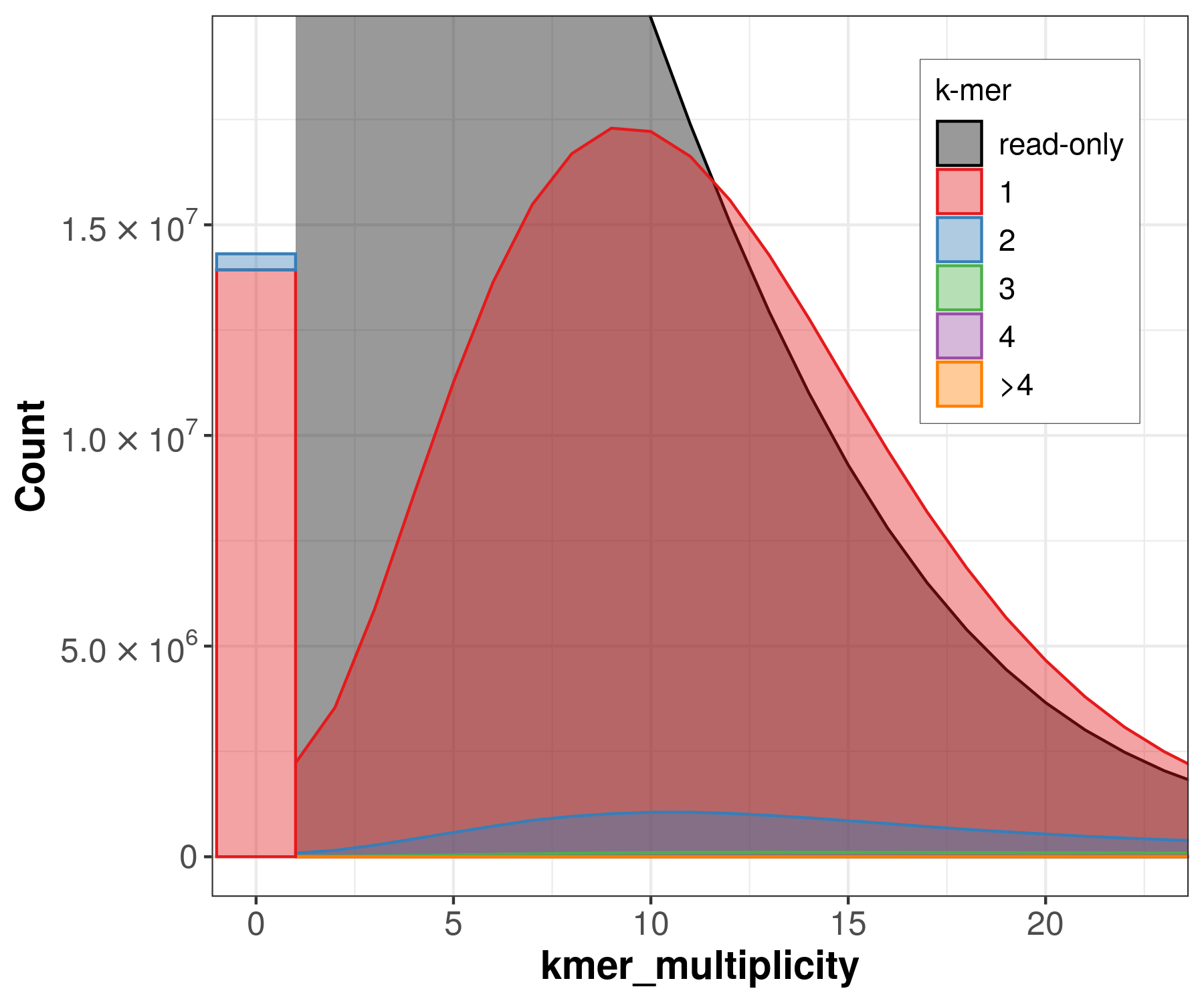

Supplement: Supplementary file 1 [file life-11-01377-s001.zip › Figures/merqury_profile.png]

Distribution

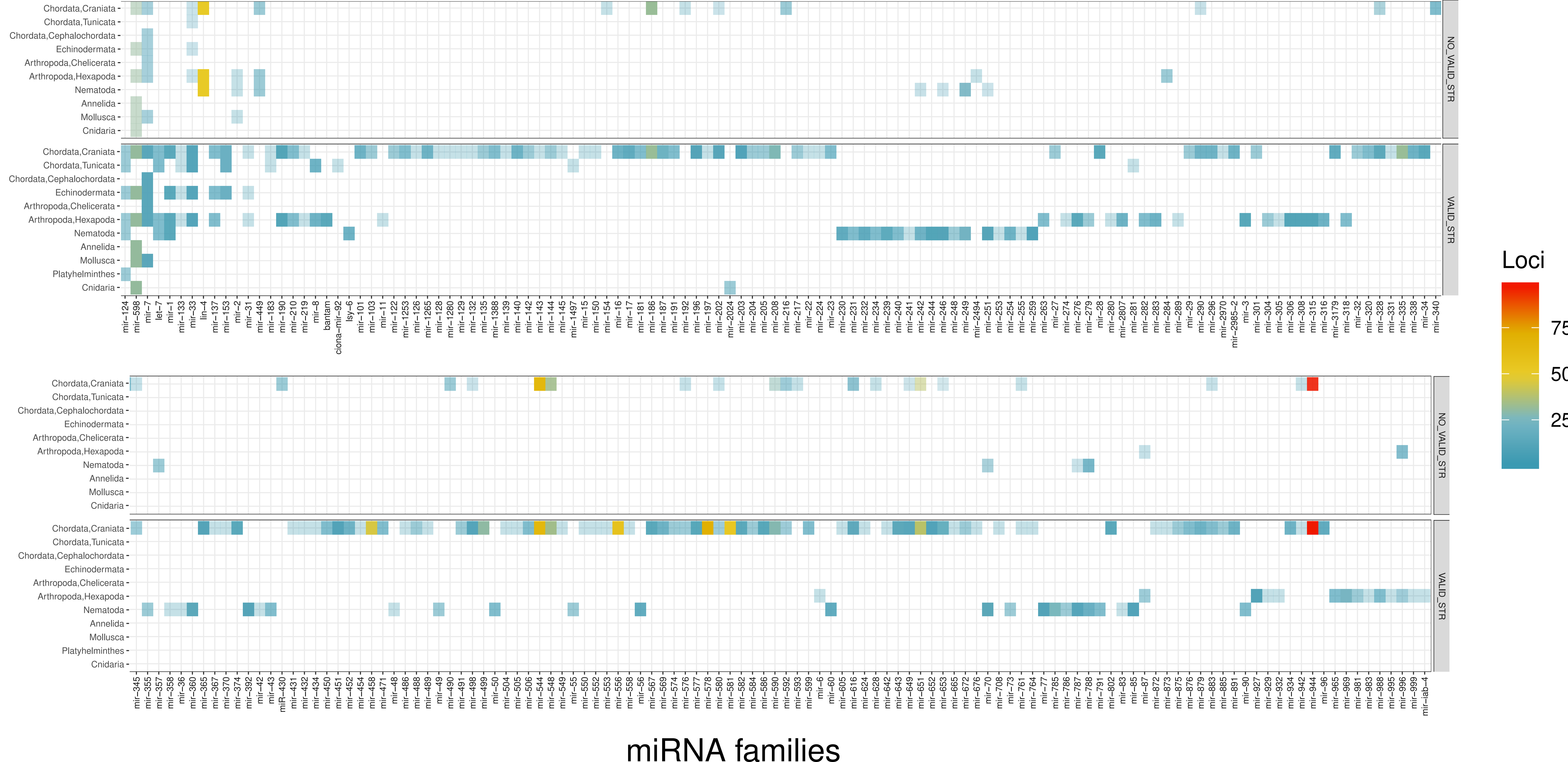

Supplement: Supplementary file 1 [file life-11-01377-s001.zip › Figures/miRNA-matrix-accepted-final.pdf]

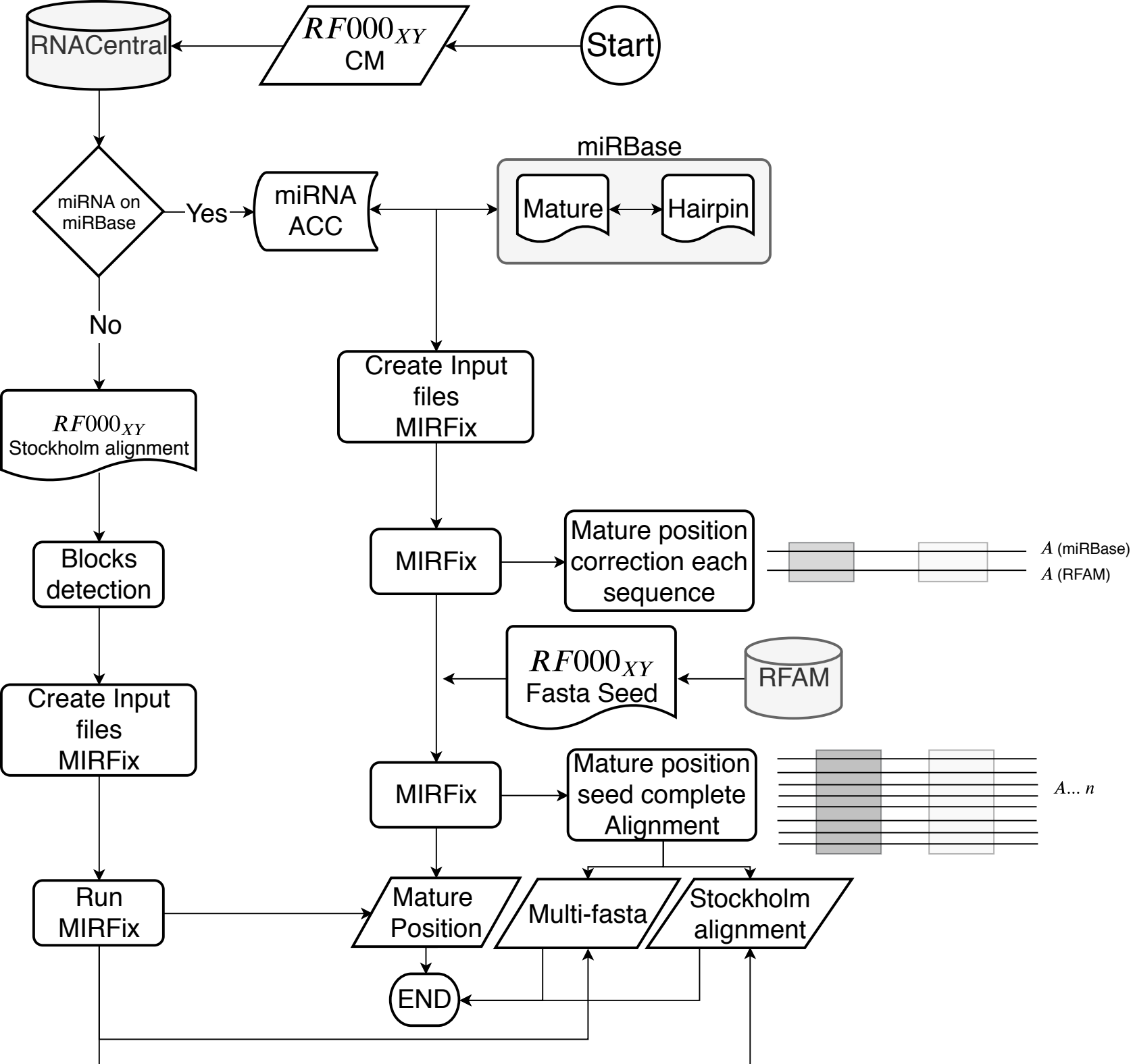

Supplement: Supplementary file 1 [file life-11-01377-s001.zip › Figures/mirnaturemirfix.pdf]

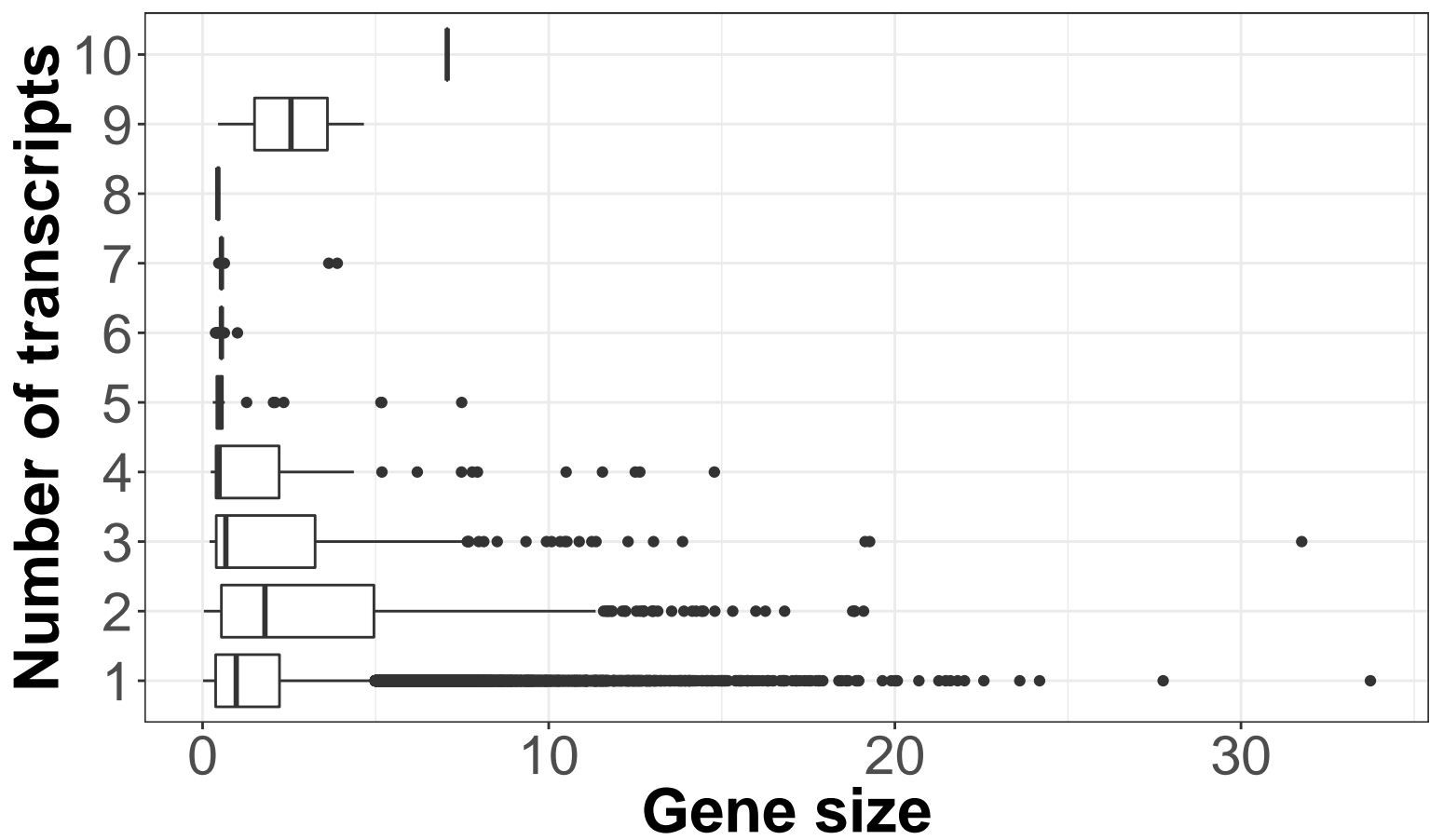

Supplement: Supplementary file 1 [file life-11-01377-s001.zip › Figures/numberTranscriptsSize.pdf]

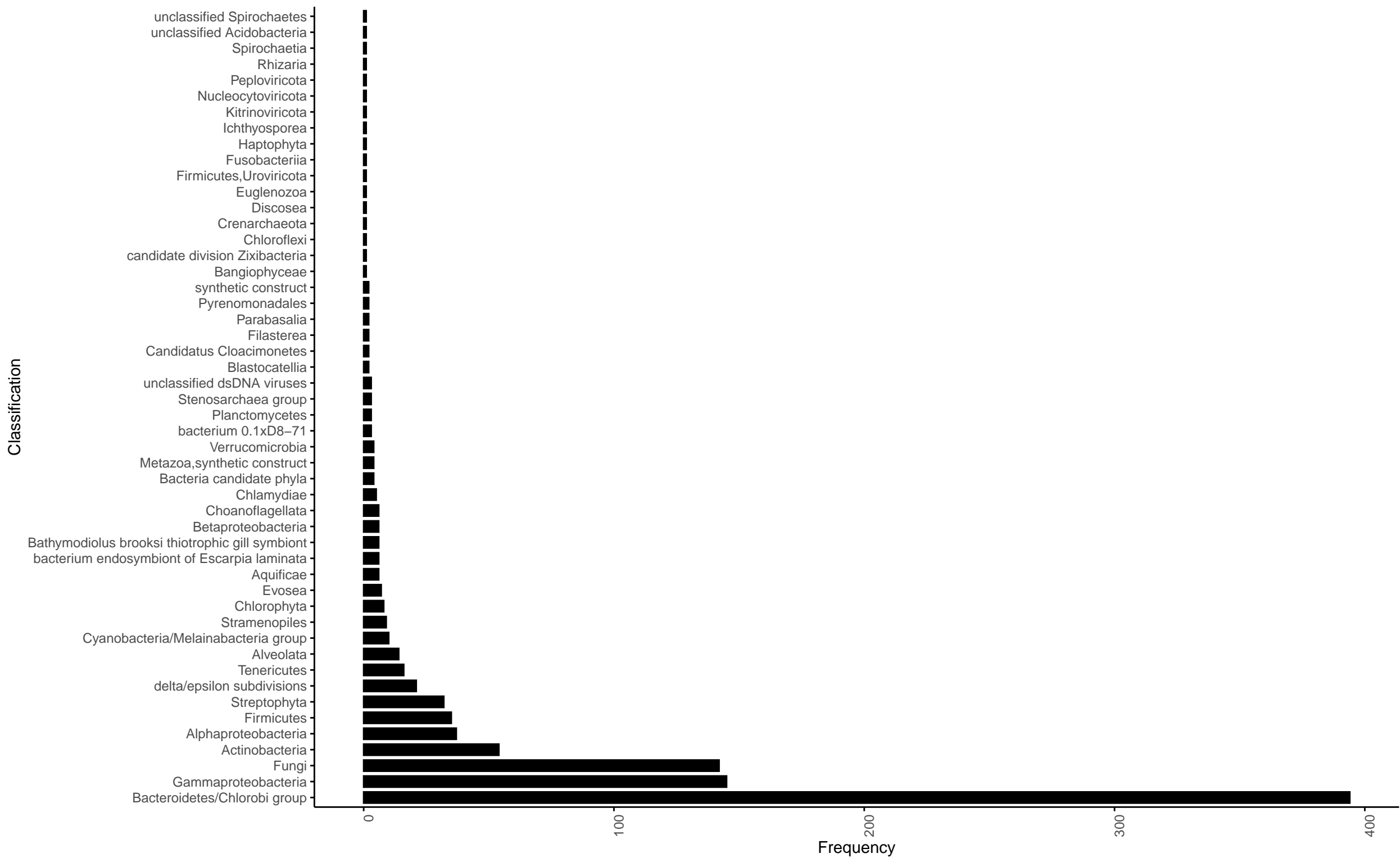

Supplement: Supplementary file 1 [file life-11-01377-s001.zip › Figures/other-groups.pdf]

A

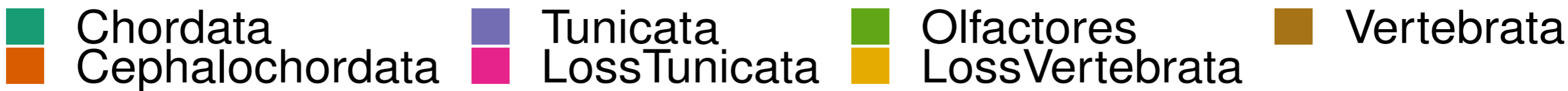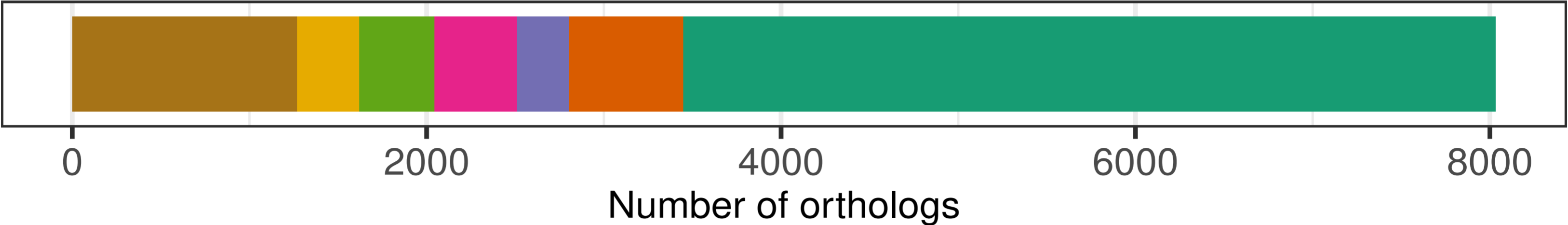

B

Didemnum vexillum specific enriched GO terms

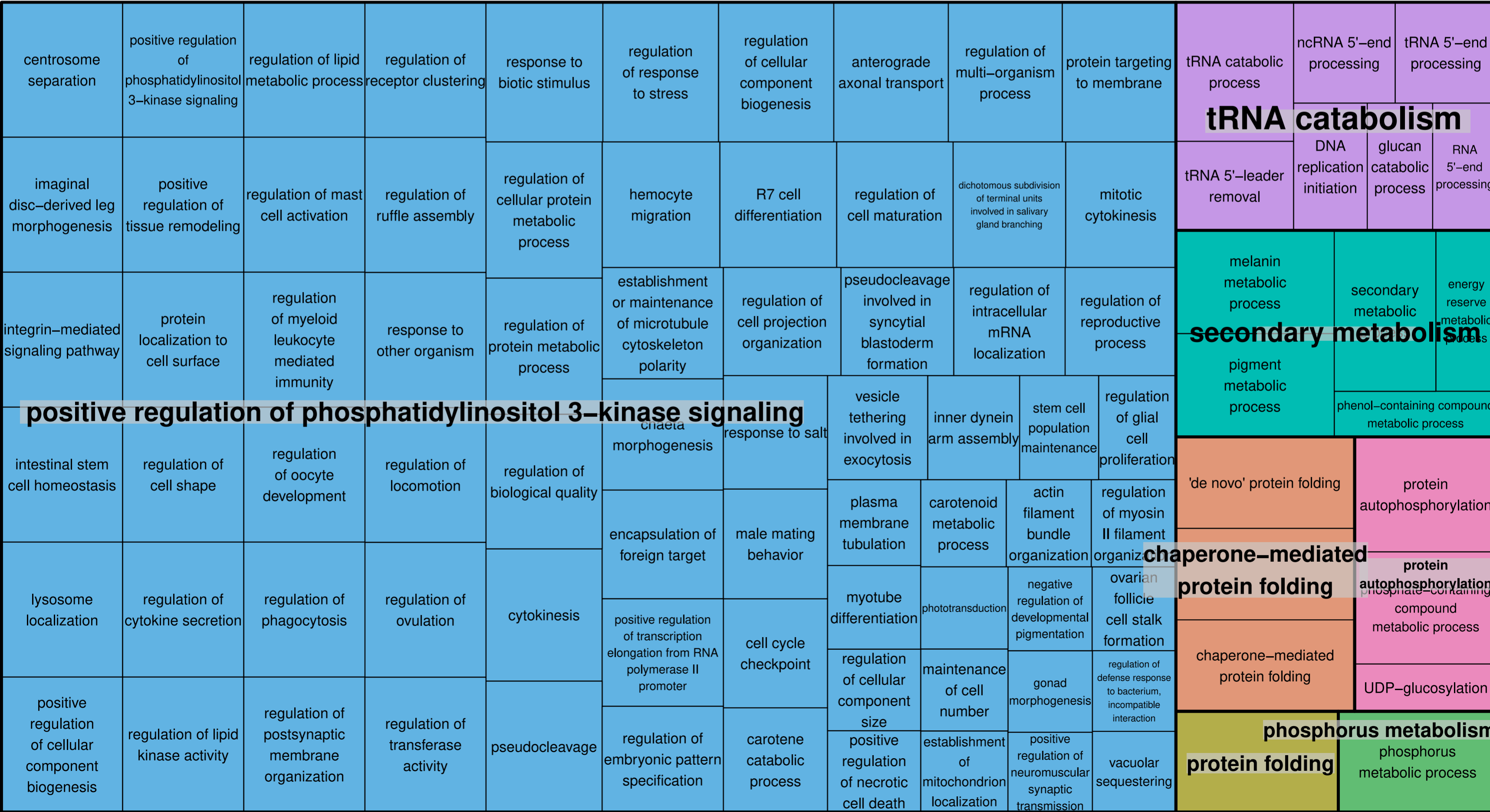

Supplement: Supplementary file 1 [file life-11-01377-s001.zip › Figures/protein-enrichment-GO-A.pdf]

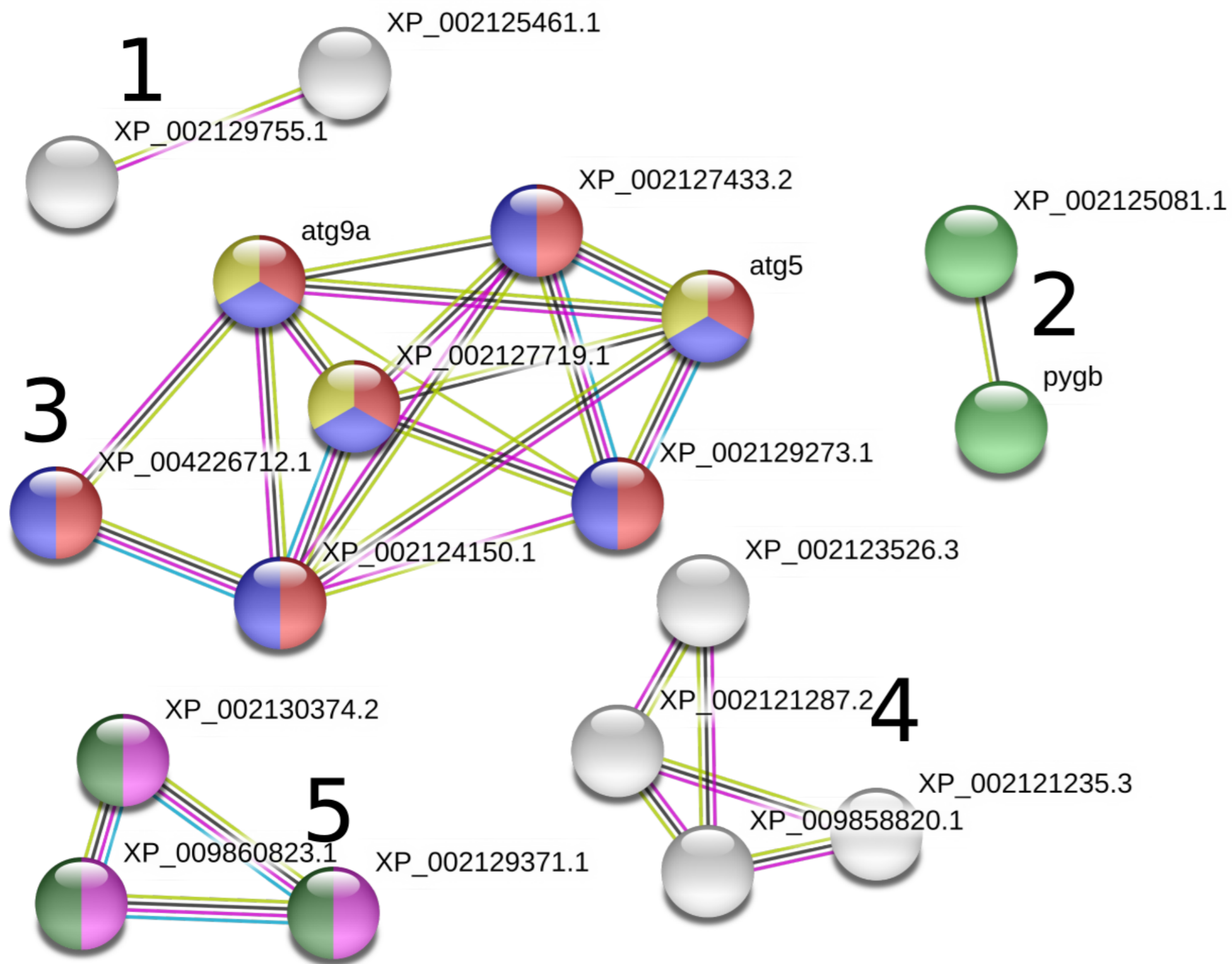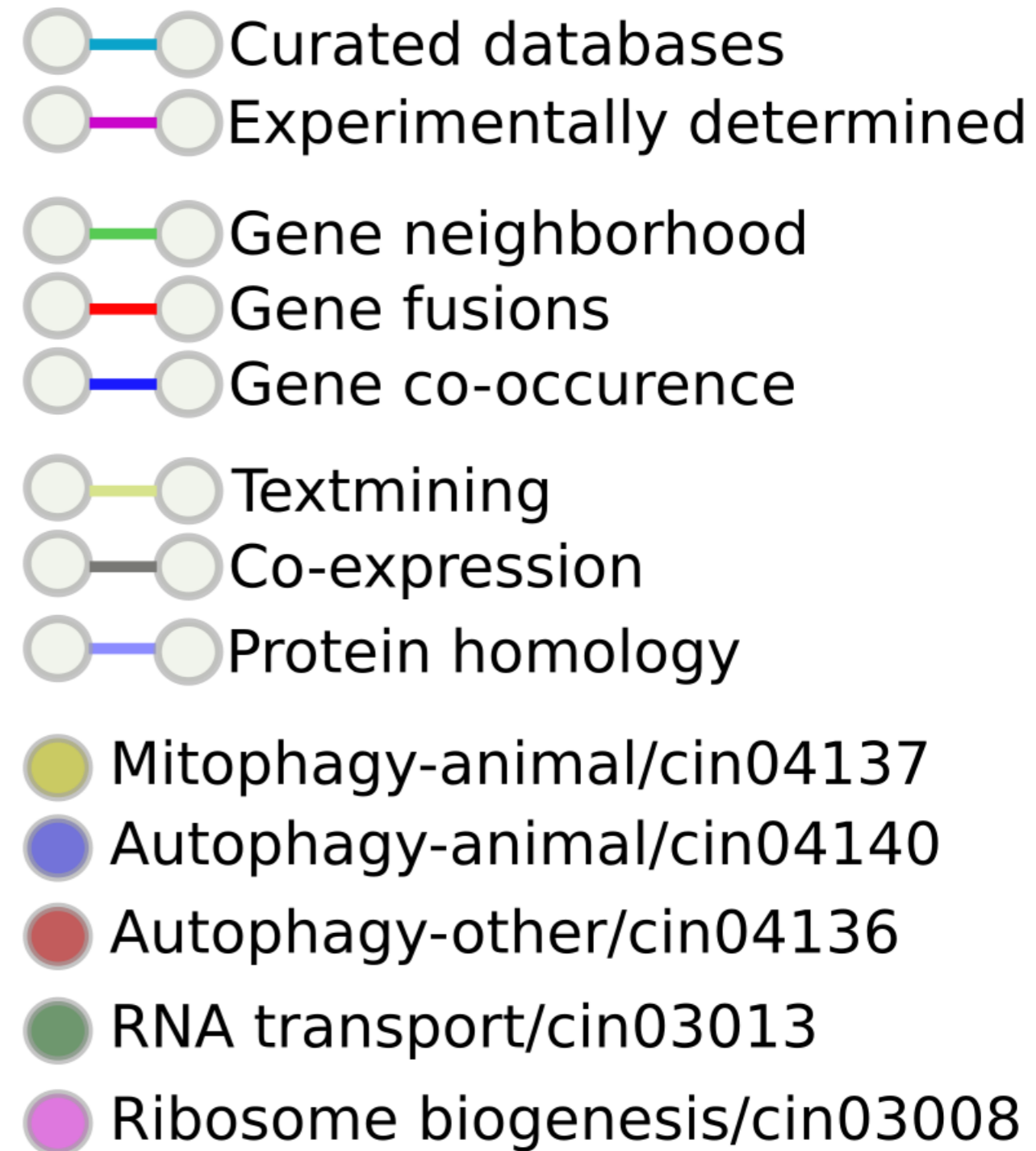

Supplement: Supplementary file 1 [file life-11-01377-s001.zip › Figures/protein-enrichment-GO-B.pdf]

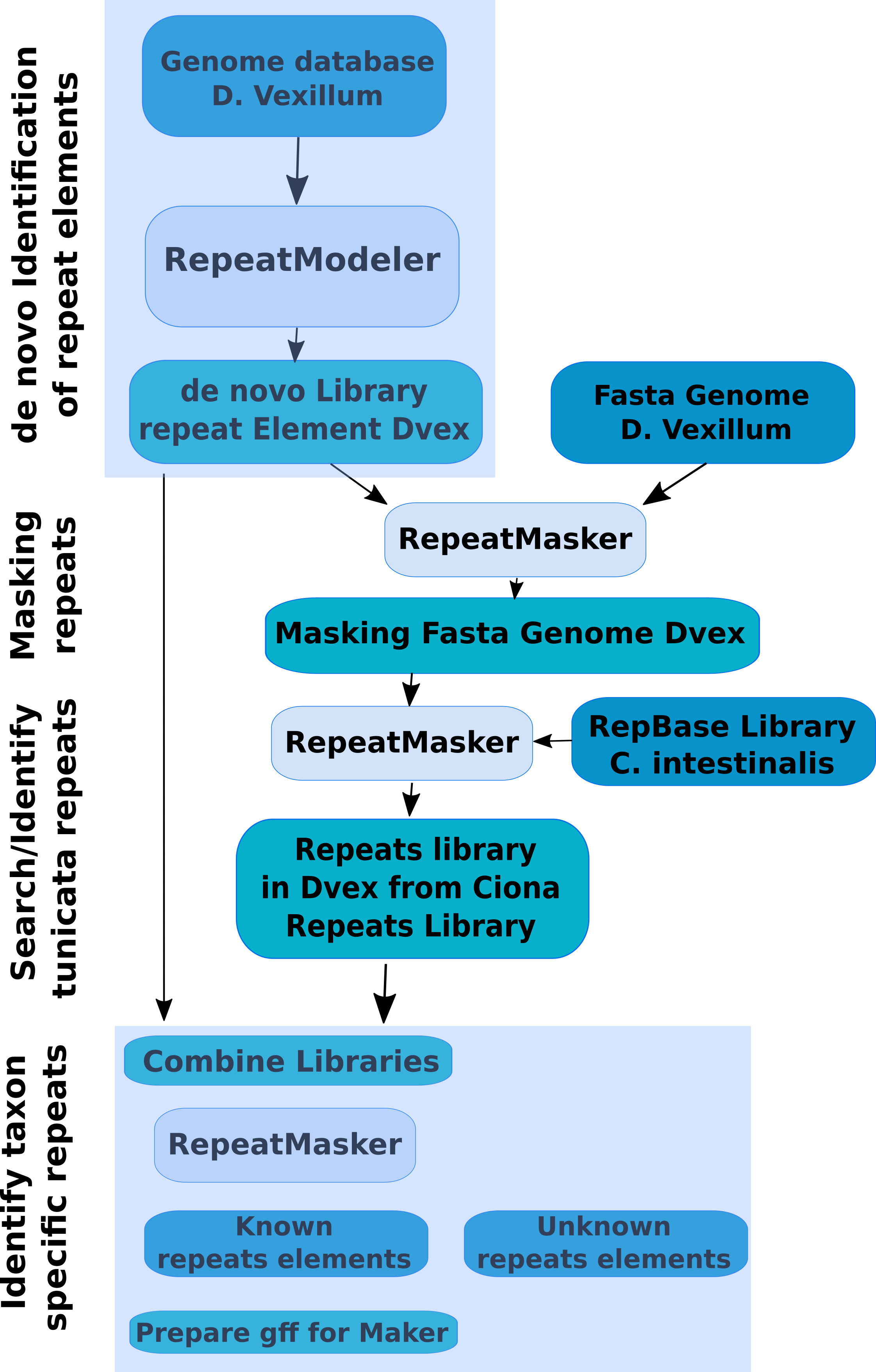

Supplement: Supplementary file 1 [file life-11-01377-s001.zip › Figures/repeats.png]

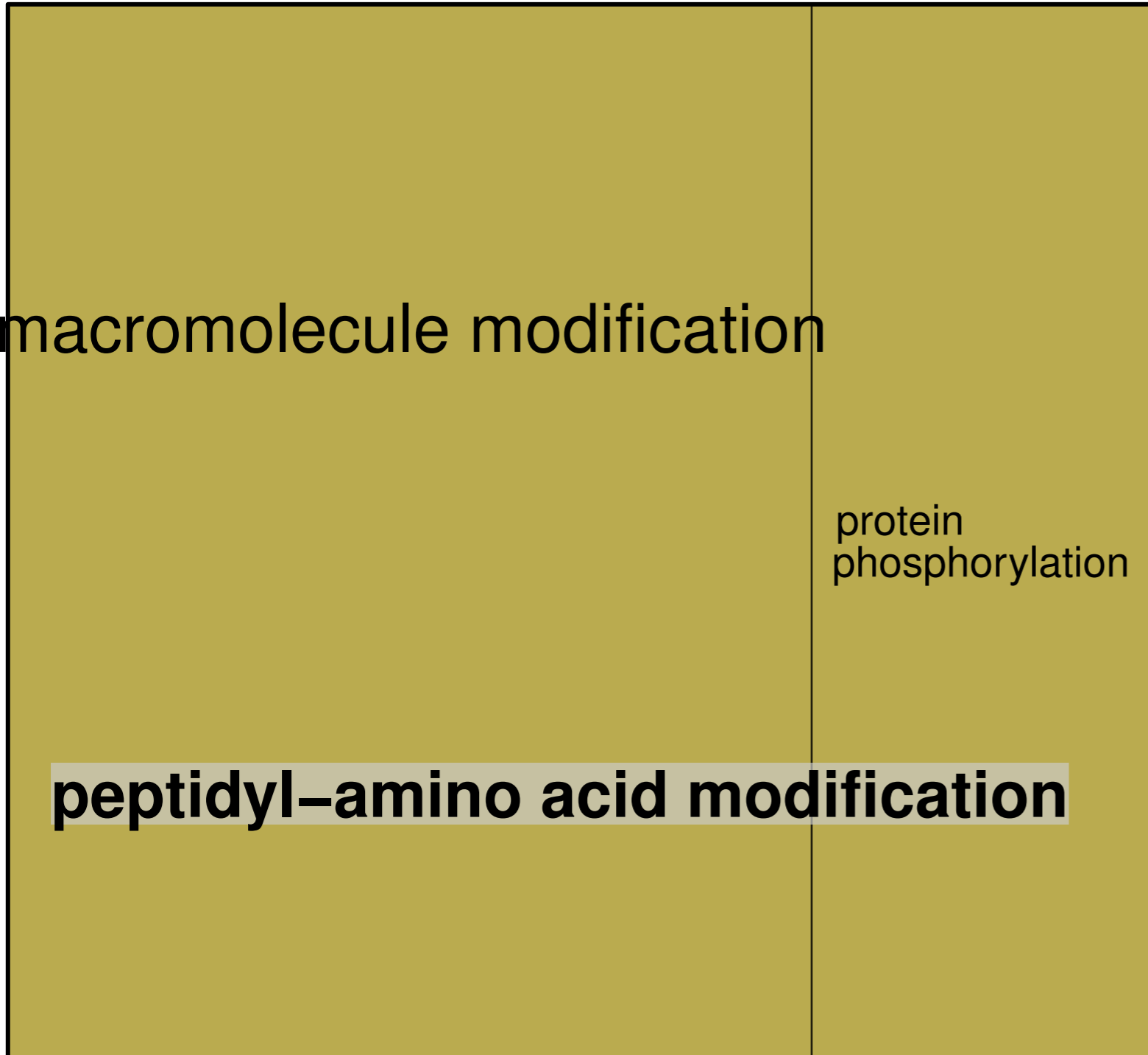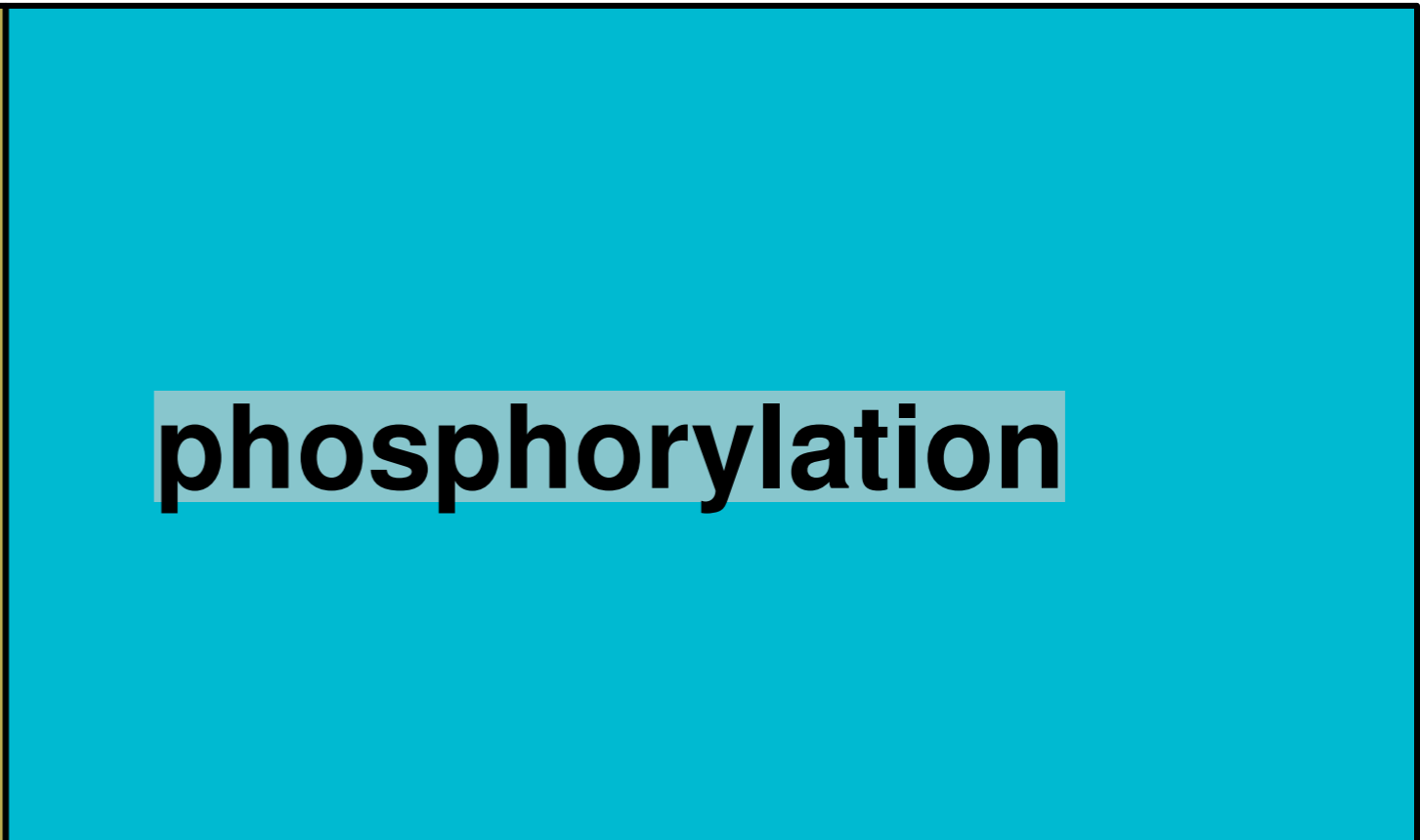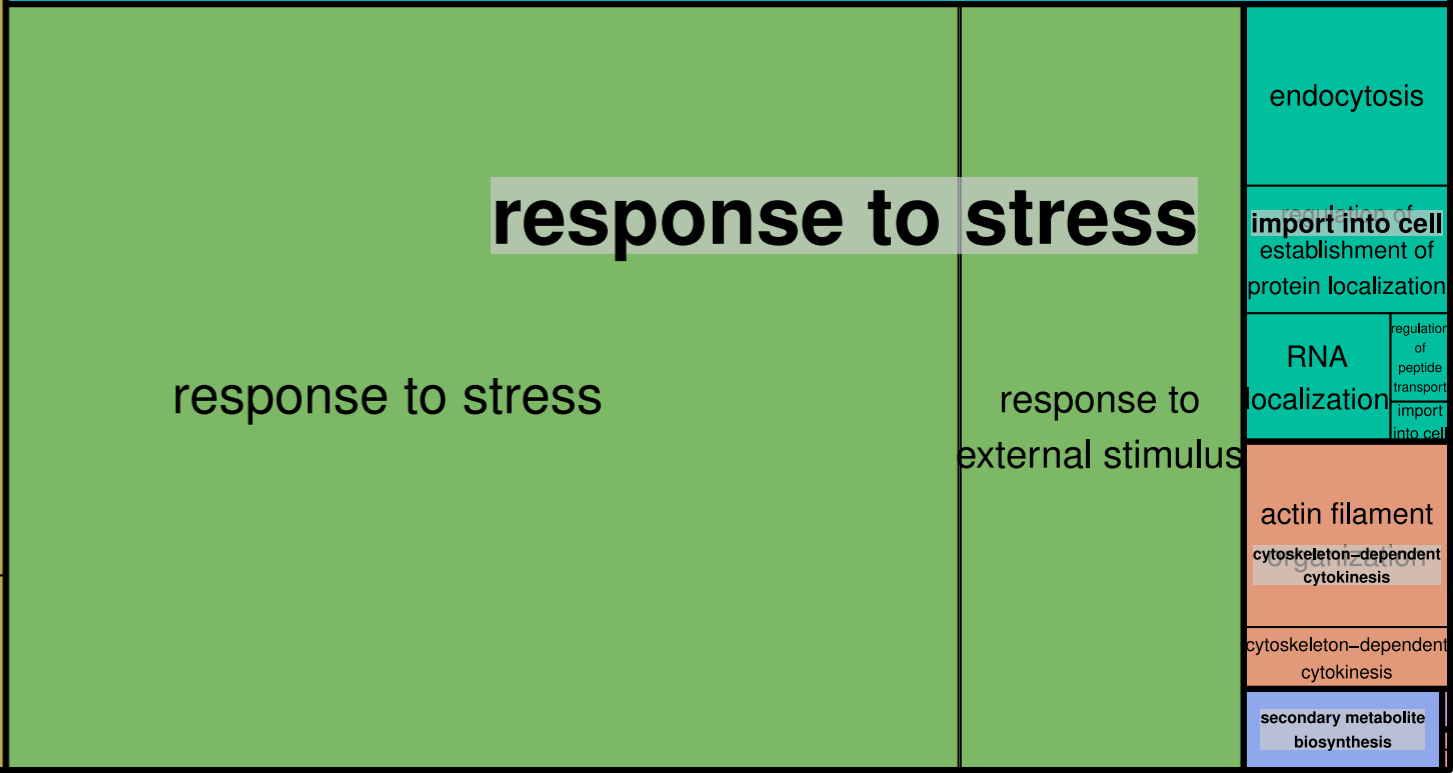

Supplement: Supplementary file 1 [file life-11-01377-s001.zip › Figures/revigo_treemap_colonial.pdf]

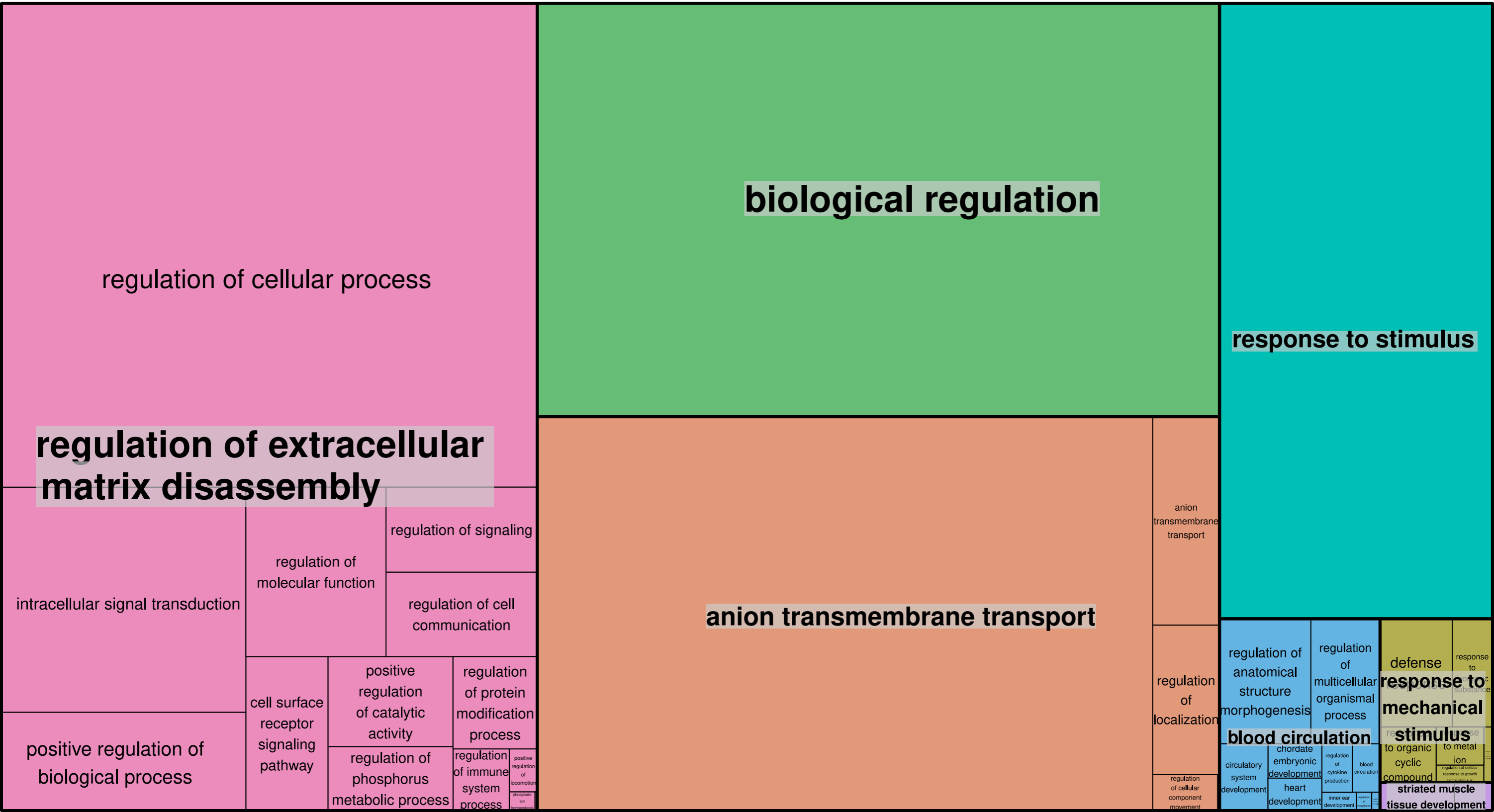

Supplement: Supplementary file 1 [file life-11-01377-s001.zip › Figures/revigo_treemap_olfactores.pdf]

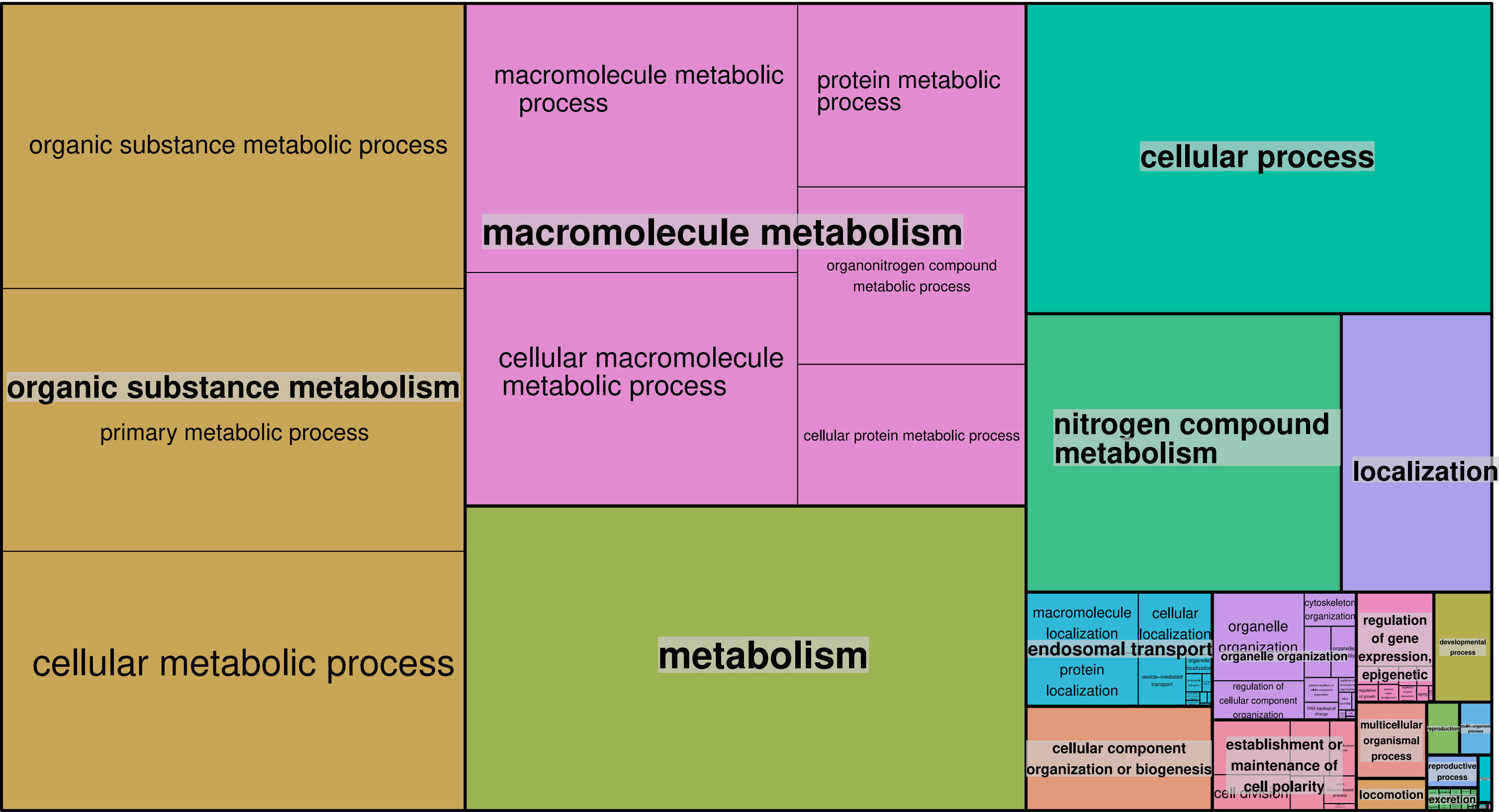

Supplement: Supplementary file 1 [file life-11-01377-s001.zip › Figures/revigo_treemap_tunicata.pdf]

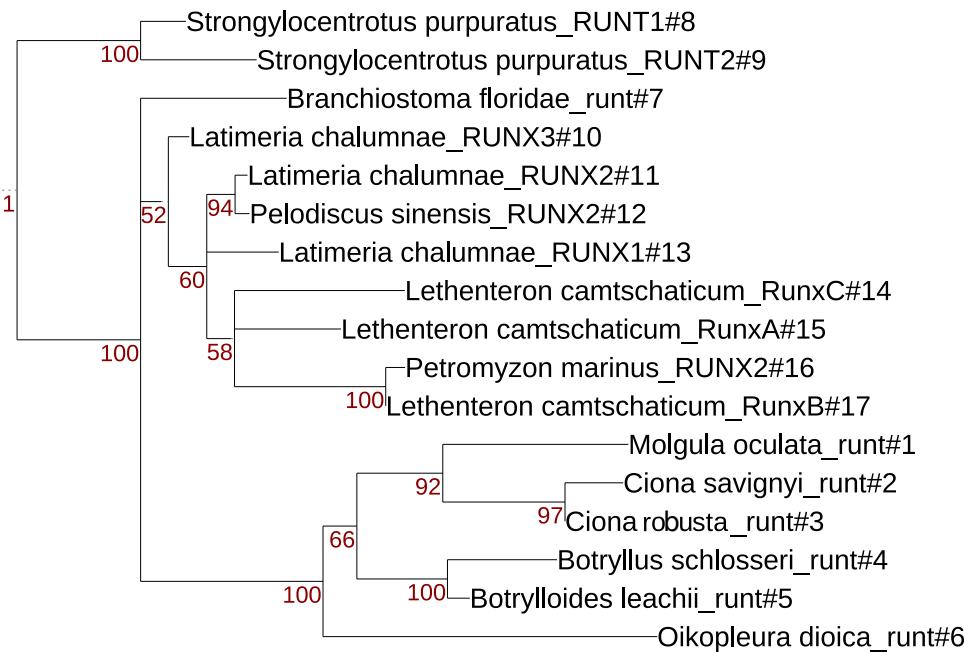

Supplement: Supplementary file 1 [file life-11-01377-s001.zip › Figures/RUNT.pdf]

A

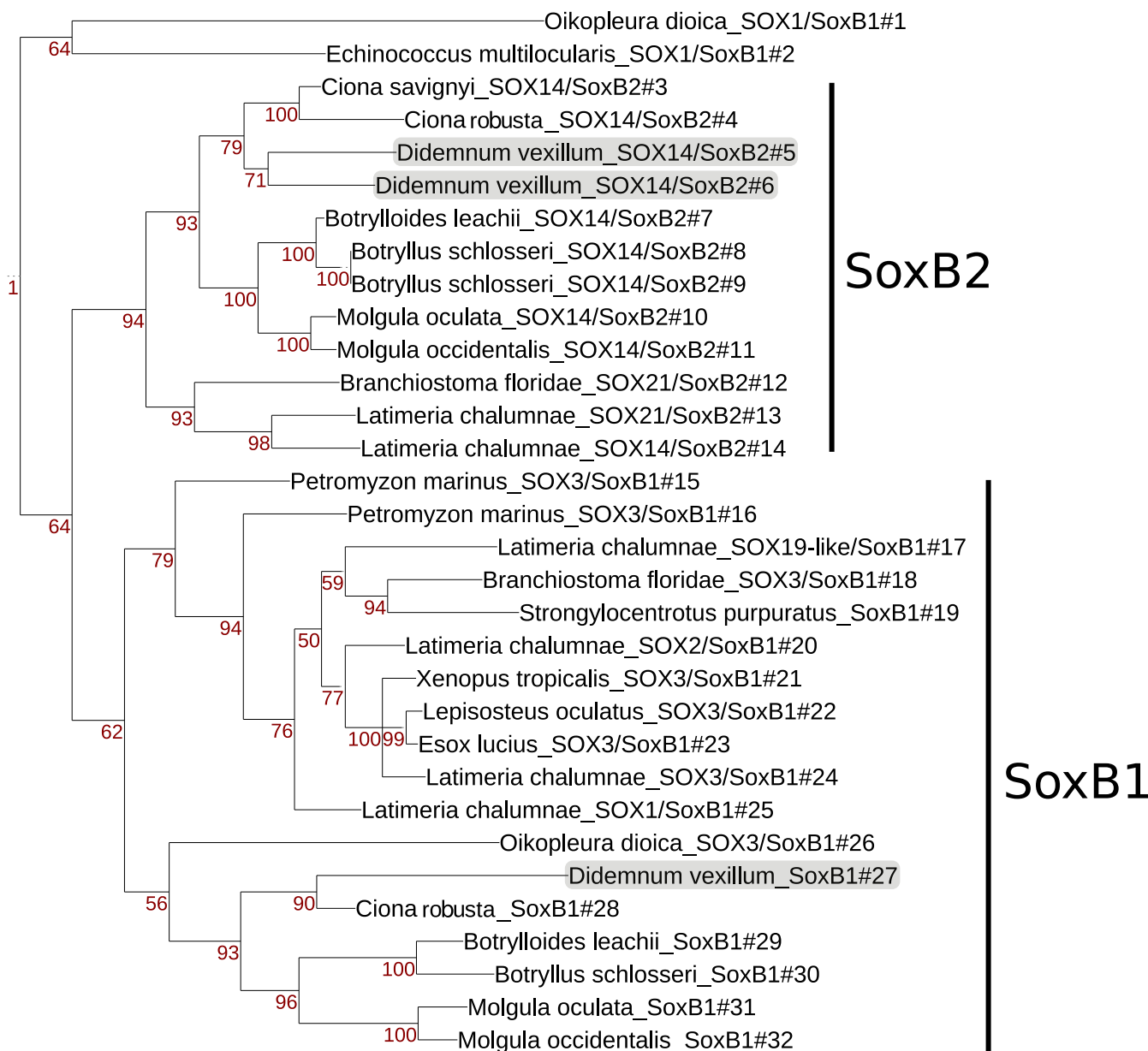

B

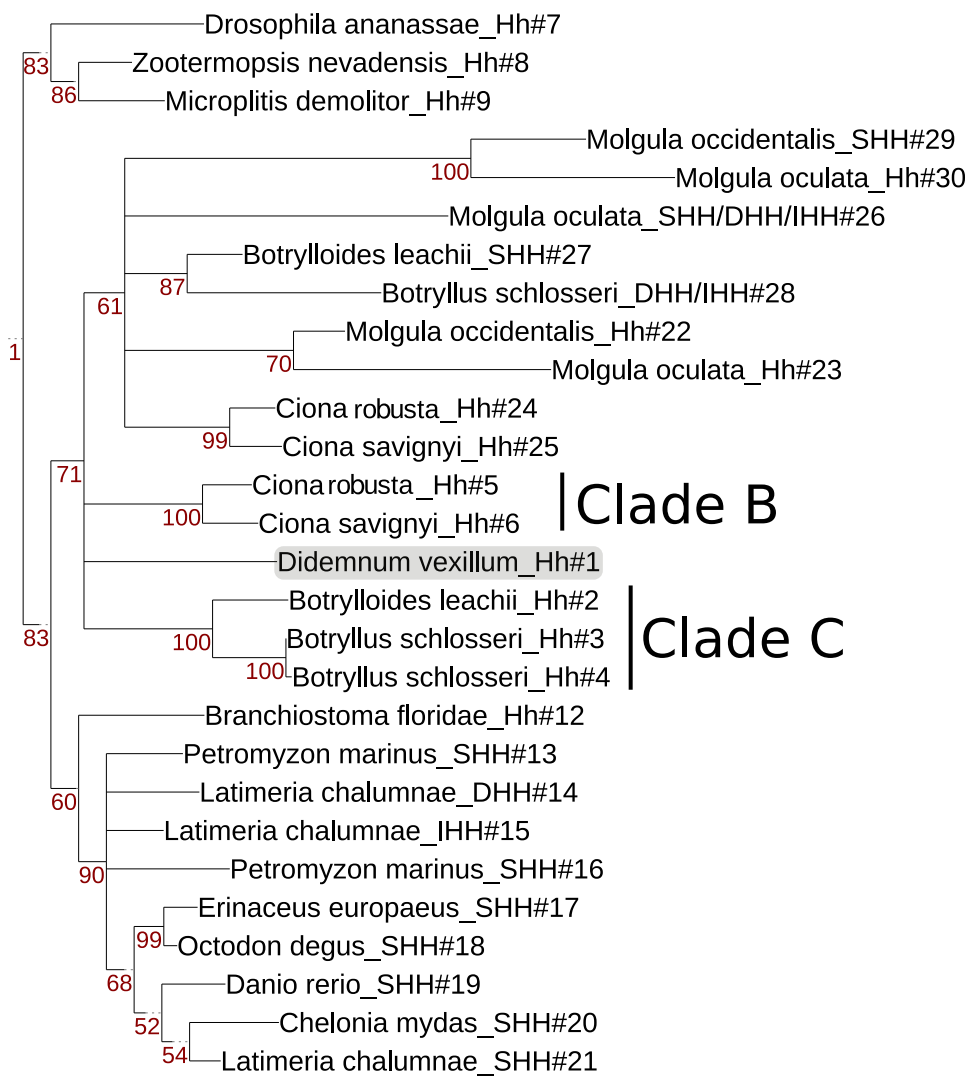

Supplement: Supplementary file 1 [file life-11-01377-s001.zip › Figures/skeletoProteins.pdf]

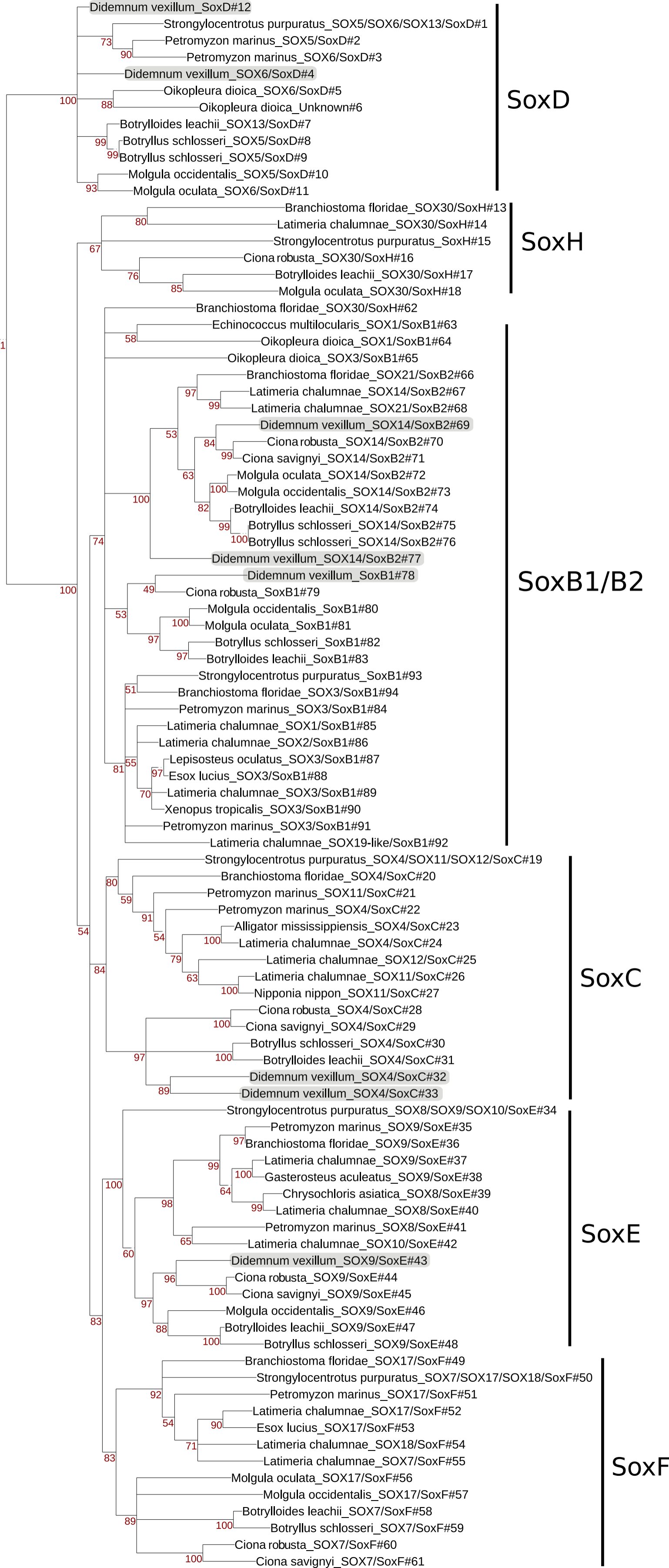

Supplement: Supplementary file 1 [file life-11-01377-s001.zip › Figures/SOX.pdf]

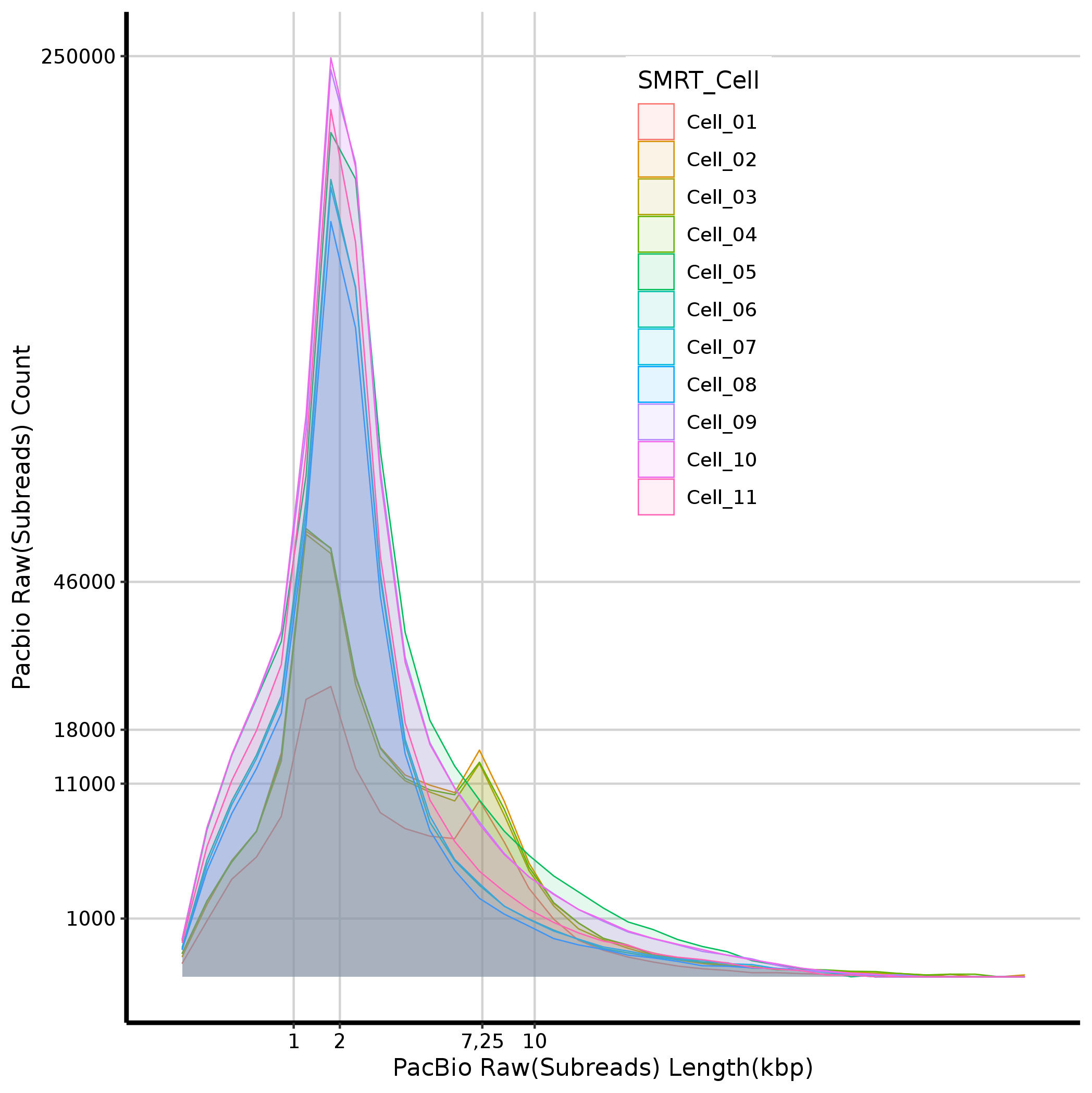

Supplement: Supplementary file 1 [file life-11-01377-s001.zip › Figures/Subreads_distribution.png]

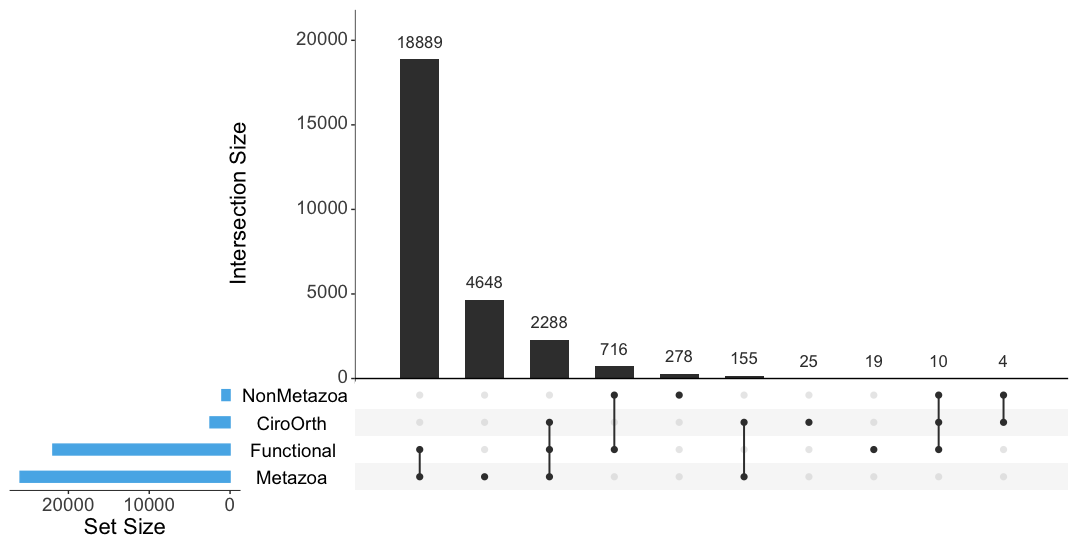

Supplement: Supplementary file 1 [file life-11-01377-s001.zip › Figures/withRelationDataset.png]
